# Adolescent Connectedness: A Scoping Review of Available Measures and Their Psychometric Properties

**DOI:** 10.3389/fpsyg.2022.856621

**Published:** 2022-05-18

**Authors:** Ezra K. Too, Esther Chongwo, Adam Mabrouk, Amina Abubakar

**Affiliations:** Institute for Human Development, Aga Khan University, Nairobi, Kenya

**Keywords:** connectedness, adolescents, measures, psychometrics, scoping review

## Abstract

**Introduction:**

Adolescent connectedness, a key component of positive youth development, is associated with various positive health outcomes. Several measures have been developed to assess this construct. However, no study has summarized data on the existing measures of adolescent connectedness. We conducted this scoping review to fill this gap. We specifically aimed to: (i) identify the existing measures of adolescent connectedness, (ii) determine the most frequently used measures among the identified measures, and (iii) summarize the psychometric properties of these measures with a keen interest in highlighting their cross-cultural utility and validity.

**Methods:**

We searched CINAHL, Embase, PsycInfo, PubMed, and Web of Science databases for relevant articles published since database inception to 7th February 2021. Our search structure contained the key words “Adolescents”, “Connectedness”, and “Measures”. We also searched Open Gray for potentially relevant gray literature.

**Results:**

We identified 335 measures from 960 eligible studies assessing various domains of adolescent connectedness, including school, family, community, peer, ethnic, racial, cultural, religious/spiritual, and self-connectedness. Most of the included studies (72.1%) were from North America and Europe. Most of the measures (*n* = 132, 39.4%) were measures of school connectedness among adolescents. Of the identified measures, 60 of them met our criteria of frequently used measures (i.e., the top five most used measures per domain of connectedness). These frequently used measures were used across 481 of the included studies with 400 of them reporting their psychometric properties. The reported reliability of these measures was adequate (Cronbach's alpha ≥ 0.70) in 89.8% of these studies. These measures also appeared to be valid in terms of their face, content, construct, criterion, convergent, discriminant, concurrent, predictive, measurement invariance, and cross-cultural validity.

**Conclusions:**

There exists a wide array of measures of adolescent connectedness. Sixty of these measures have been frequently used across studies and appear to be reliable and/or valid. However, this evidence is mostly from North America and Europe. This is a reflection of the limitation of this review where only studies published in English were considered. It might also reflect the paucity of research in other regions of the world. More research is needed for clearer insights.

## Introduction

In recent years, there has been an increasing interest in positive youth development [PYD] (Qi et al., 2020). PYD encompasses six key components termed the 6Cs, including connection, character, caring, confidence, competence, and contribution (Shek et al., 2019). In this work, we focus on connection, hereinafter referred to as connectedness. A sense of connection or connectedness is defined as a sense of belonging, feelings of mutual support, acceptance, safety, respect, engagement, and inclusion to certain contexts (Resnick et al., 1997; Unger et al., 2000; Aydin and Oztütüncü, 2001; Dornbusch et al., 2001). Adolescent connectedness can take place in various contexts including self, peer, family, school, and community (Jose Paul et al., 2012). This is dependent on an adolescent's social environment and the activities, people, and places that he/she interacts with in that environment (Abubakar et al., 2014).

Connectedness in adolescents is associated with various positive outcomes. For instance, research shows that school connectedness is linked to positive school adjustments, improved academic achievement and enhanced psychosocial status and general health (Bersamin et al., 2019). Among Norwegian adolescents, research showed that a sense of class belonging and support from teachers influenced students' psychological and mental wellbeing (Brandseth et al., 2019). In Kenya, a higher sense of belonging among Kenyan adolescents, both native and those of immigrant backgrounds, was associated with improved life satisfaction (Abubakar et al., 2014).

Adolescent connectedness has also been documented as an important protective factor against various negative outcomes among adolescents. Adolescents with higher connectedness are less likely to have suicidal ideations, have reduced sexual risk tendencies and substance use, and are less likely to have emotional and behavioral problems (Resnick et al., 1997; Joyce Hilary and Early Theresa, 2014; Langille et al., 2015). Among Kenyan adolescents, school, ethnic, and religious connectedness was shown to act as a buffer against poor mental health functioning (Abubakar et al., 2014). School disconnectedness has been identified as one of the major factors that impede academic achievement and optimal health outcomes among in-school adolescents (Monahan et al., 2010; Niehaus et al., 2012).

The positive impacts of adolescent connectedness are long-lasting and may go way beyond the adolescence stage into adulthood. Evidence from the United States show that school and family connectedness in adolescence conferred protection against multiple health risks later in life such as sexual risk behaviors, violence perpetration and victimization, substance abuse, emotional distress, and suicidal ideation (Steiner et al., 2019). Additionally, in New Zealand, family connectedness has been documented to mitigate against overall decline in wellbeing over time (Stuart and Jose Paul, 2014). Similarly, in a study from Australia, adolescents who were connected to their peers were less likely to have mental health issues such as social anxiety in adulthood (Rapee et al., 2020). In a longitudinal study among adolescents in New Zealand, global connectedness (peer, family, school, and neighborhood connectedness) was associated with overall psychological wellbeing over time (Jose Paul et al., 2012).

Because of the positive impacts of connectedness, it has become a target for programmes and interventions aimed at promoting health and reducing health risk behaviors. For instance, findings from a systematic review that aimed to identify programmes that increased school connectedness showed that there were a number of such interventions that had been incorporated into school programmes to reduce behaviors such as absenteeism and to promote school performance (Chapman et al., 2013). Such interventions have the potential to impact on the overall wellbeing of adolescents. For instance, an intervention to increase social connectedness in street-involved youth reduced hopelessness and despair (McCay et al., 2011). Given the overwhelming evidence on the potential benefits to connectedness there is a need to invest in programmes aimed at enhancing adolescent connectedness.

To adequately assess adolescent connectedness and evaluate the effectiveness of such interventions, there is a need for culturally appropriate measures that are psychometrically sound across contexts. Over the years, several measures have been developed to evaluate different domains of connectedness. These include, among others, the Psychological Sense of School Membership Scale [PSSM] (Goodenow, 1993), the Hemingway's Measure of Adolescent Connectedness [HMAC] (Karcher, 2001), the Multigroup Ethnic Identity Measure [MEIM] (Phinney, 1992), and the Social Connectedness Scale (Lee and Robbins, 1995). These measures were developed to either measure specific domains of adolescent connectedness or multiple domains concurrently.

Despite the existence of several measures of adolescent connectedness, to the best of our knowledge, no study has synthesized data on existing measures, including their psychometric properties. Furthermore, there is a dearth of information regarding the most frequently used measures of connectedness among adolescents and their psychometric properties, including, their utility and validity across cultural contexts. There is a need for summarizing the existing measures of adolescent connectedness and their psychometric robustness for research, program implementation and programme evaluation purposes. To fill these gaps, we conducted a scoping review to identify existing measures of adolescent connectedness and their psychometric properties. The aim of this review was to:

- Identify the measures of adolescent connectedness currently in use.
- Identify the most frequently used measures among the identified measures.
- Determine the psychometric properties of the most frequently used measures of connectedness currently in use among adolescents, including their cross-cultural utility and validity.

## Methods

### Identification of Relevant Studies

#### Search Strategy

We conducted a search in five electronic databases (CINAHL, Embase, PsycInfo, PubMed, and Web of Science) for articles published from database inception to 1st March 2022 (when the last search was conducted). Our search structure contained the key words “Adolescents”, “Connectedness”, and “Measures” combined by the Boolean operator AND. Respective synonyms for these key words were combined using the OR Boolean operator (See Supplementary Material 1 for the search strategy). We restricted the search to only peer reviewed articles. Finally, we searched the Open Gray Database for any potential articles meeting our eligibility criteria.

#### Eligibility Criteria

Table 1 shows the eligibility criteria for this review.

**Table 1:** Study selection criteria.

**Criterion**	**Inclusion**	**Exclusion**
Geographical location	Global	None
Population	Adolescents aged 10–19 years^*^^#^	Age outside 10–19 years
Language	English^$^	Non-English
Evidence sources	Empirical studies	Non-empirical studies such as commentaries, case reports, reviews

* *Where the grade level was reported instead of age, we included those studies with the reported age grades falling into the 10 to 19-year range based on the available respective country statistics*.

# *For the studies that combined adolescents with children or adults (i.e., the reported age range extending beyond the 10-19 range), we included those studies with the reported mean or median age falling within our target age range*.

$ *We only included studies published in English because this was the only language that the reviewers could understand*.

*Where there were multiple studies from a similar project, we included the more comprehensive study*.

### Selection of Studies

The identified articles were retrieved and uploaded to EppiReviewer Web software (https://eppi.ioe.ac.uk/EPPIReviewer-Web/home) for data management. Three authors (EC, AM, and ET) equally distributed the articles among themselves and independently screened the articles by title, abstract and full text. The three reviewers were in consultation at every stage to address arising issues and resolve disagreements. Where necessary, a senior member of the team (AA) was involved.

### Data Charting

Data charting was conducted in the EppiReviewer Web software. We extracted the following information from each of the included studies: (I) Study characteristics (Name of the first author, year of publication, country of study, study design, sample size, adolescent population involved, age of participants, and gender proportions), (ii) Domains of connectedness assessed (self, peer, family, school, or community), and (iii) Characteristics of the measures used (the measure of adolescent connectedness used and the reported psychometric properties [in this review, we only included the reported reliability and/or validity estimates based on data from that particular study, but not those cited from previous studies)].

### Data Analysis

We used descriptive statistics (frequencies and percentages) to summarize the number of studies assessing each domain of connectedness. We summed the total number of measures of adolescent connectedness as reported in the included studies. We then distributed these measures to the respective domains of connectedness that they assessed and computed their frequency of use. In this study, we defined the most frequently used measure as the top five most used measures per domain. Psychometric information (the reported reliability and/or type of validity) were extracted from the eligible studies and summarized per measure. The cross-cultural utility of the measures was implied when a measure was used across countries. We categorized the countries in which the included studies were conducted into their respective continents and summarized their distribution.

## Results

### Results of Database Search

**Figure 1:**
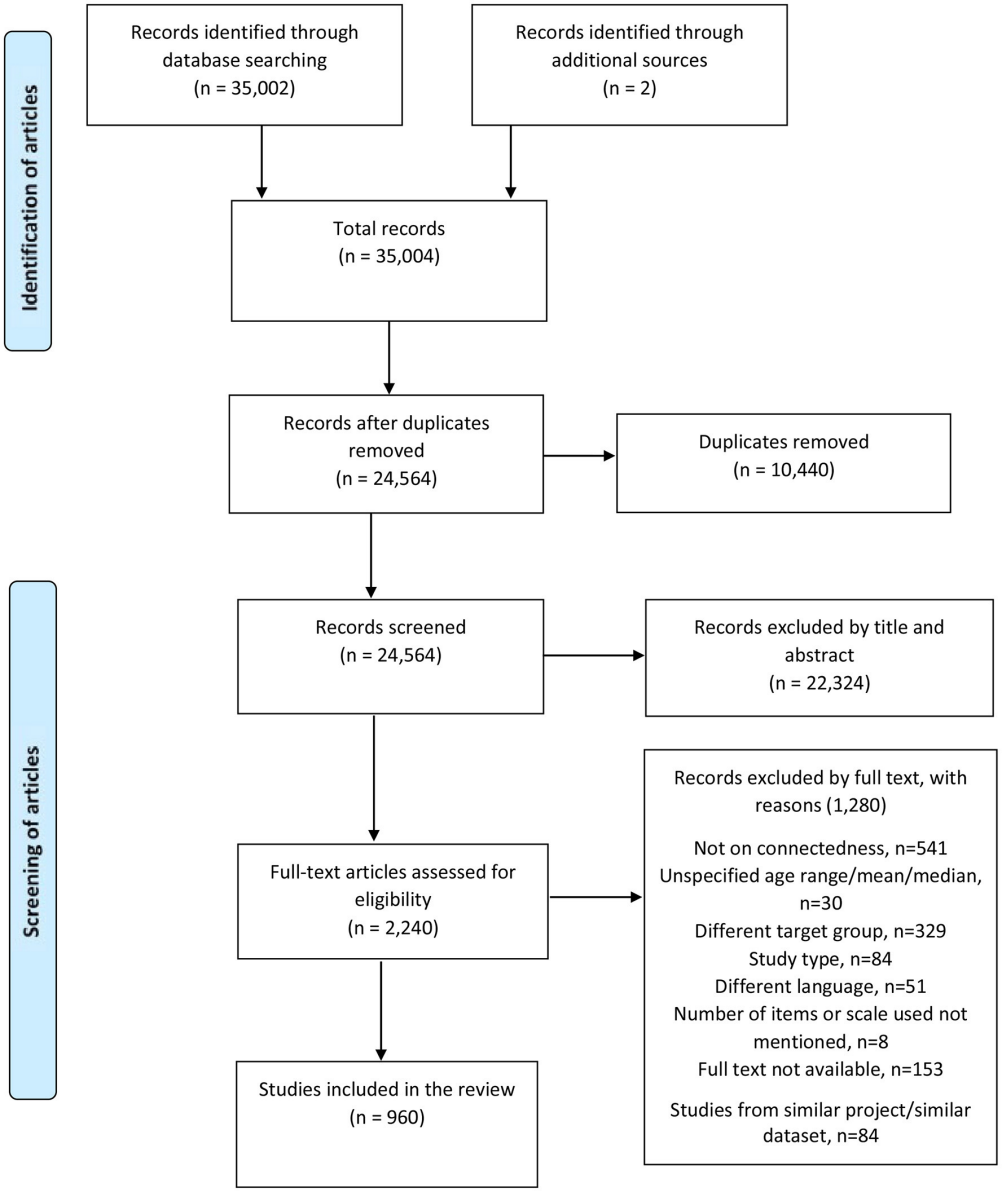
Flowchart for the scoping review process.

We identified a total of 35,002 articles from the electronic database search. After removing duplicates and screening articles by eligibility criteria, we included 960 studies in the review. Figure 1 shows the study selection flowchart for the scoping review process.

### Characteristics of Included Studies

Supplementary Material 2 shows in detail the characteristics of the included studies. In summary, the majority of the included studies (53.0%, *n* = 509) were conducted in North America. The remaining studies were distributed across Europe (19.1%, *n* = 183), Asia (11.5%, *n* = 110), Oceania (9.4%, *n* = 90), Africa (1.9%, *n* = 18), and South America (1.6%, *n* = 15). A few (3.6%, *n* = 35) of the included articles were multi-country studies. The included studies were published in the period from 1990 to 2021 with the majority (80.5%, *n* = 773) being published from 2010 onwards.

The majority of the included studies exclusively assessed specific domains of adolescent connectedness including school connectedness (45.4%, *n* = 436 studies), family connectedness (12.4%, *n* = 119 studies), community connectedness (2.7%, *n* = 26 studies), peer connectedness (5.5%, *n* = 53 studies), self-connectedness (0.3%, *n* = 3 studies), ethnic identity/connectedness (3.1%, *n* = 30 studies), racial identity/connectedness (0.2%, *n* = 2 studies), cultural connectedness (0.6%, *n* = 6 studies), and religious/spiritual connectedness (0.4%, *n* = 4 studies). In 281 (29.3%) of the remaining studies, two or more of these domains were assessed concurrently.

From the included studies, a total of 335 individual measures of adolescent connectedness were identified (see Supplementary Material 3 for the full list of these measures). About a fifth (20.4%, *n* = 196) of the included studies did not use any of the existing or newly developed measures. Instead, study-specific items (either adapted from previous studies or newly created for that study) were used to assess adolescent connectedness. Of the included studies, 756 studies reported an aspect of reliability and/or validity of these measures or items. Of these studies, 77 studies were development and/or validation studies of the various measures of adolescent connectedness. The reported psychometrics were mostly reliability (internal consistency, test-retest, or interrater reliability) except in 158 studies that additionally reported validity (Supplementary Material 2).

### Measures of School Connectedness Among Adolescents and Their Psychometric Properties

We identified a total of 132 individual measures which were used to assess school connectedness among adolescents (Supplementary Material 4A presents the list of these measures including their frequency of use). Among these measures, the PSSM (Goodenow, 1993), the School Connectedness Scale [SCS] (McNeely et al., 2002), the Basic Psychological Needs Satisfaction Scale [BPNS] (Ryan and Deci, 2000), the School Belongingness Scale (Arslan and Duru, 2017), the School Belonging Scale (Anderman, 2002), and the Need for Relatedness Scale (Richer and Vallerand, 1998) were the most frequently used measures. These top five measures were used across 236 of the included studies, with 202 of them reporting an aspect of reliability or validity. We have summarized the psychometric properties of these measures in Table 2. Detailed psychometric properties of all the measures of school connectedness are in Supplementary Material 2.

**Table 2:** Psychometric properties of the most frequently used measures of adolescent school connectedness.

**Measure used**	**Number of studies**	**Reliability**	**Validity**						
		**Internal consistency**	**Construct validity**	**Convergent validity**	**Discriminant validity**	**Concurrent validity**	**Predictive validity**	**Content validity**	**Measurement invariance**
Psychological Sense of School Membership Scale (PSSM)	109	-Reliability reported in 96 studies [94] studies reported (Cronbach's alpha (range = 0.55–0.97); two studies reported McDonald's Omega (range = 0.82–0.96)] -85 (90.4%) studies reported Cronbach's alpha ≥ 0.70	Established in 11 studies	Established in three studies	Established in three studies	Established in one study	Established in one study	Established in one study	-Measurement invariance across age, gender, time points and poverty quantile groupings established in two studies -Measurement invariance and functional equivalence across countries established in one study -Measurement invariance across cultural groups established in one study
School Connectedness Scale (SCS)	84	-Reliability reported in 71 studies [70 studies reported Cronbach's alpha (range = 0.62–0.89); one study reported McDonald's Omega (0.81)] -66 (94.3%) studies reported Cronbach's alpha > 0.70	Established in six studies	Established in two studies	NR	NR	NR	NR	NR
Basic Psychological Needs Satisfaction Scale (BPNS)	21	-Cronbach's alpha reported in 16 studies (range = 0.62–0.88) -14 (87.5%) studies reported Cronbach's alpha > 0.70	Established in three studies	Established in one study	Established in one study	NR	NR	NR	Measurement invariance across age groups established in one study
School Belonging Scale	8	-Cronbach's alpha reported in all the eight studies (range = 0.74–0.92)	Established in two studies	Established in one study	Established in two studies	NR	NR	Established in one study	NR
School Belongingness Scale	7	-Cronbach's alpha reported in six studies (range = 0.61–0.87) -Five (83.3%) studies reported Cronbach's alpha > 0.70	Established in three studies	Established in one study	NR	NR	NR	NR	Measurement invariance across gender established in one study
Need for Relatedness Scale	7	-Cronbach's alpha reported in four studies (range = 0.85–0.95)	NR	NR	NR	NR	NR	NR	NR

*NR, not reported*.

The reported internal consistency of these measures across studies was above the recommended acceptable threshold of 0.70 except for some subscales or time points in measures used in 16 studies (Poteat and Espelage Dorothy, 2007; Prelow Hazel et al., 2007; Sun et al., 2013; Lam Un et al., 2015; Bolland Kathleen et al., 2016; Espelage Dorothy et al., 2016; Gnambs and Hanfstingl, 2016; Rose Chad et al., 2016; Thumann Barbara et al., 2016; Cowden Richard et al., 2018; Tomek et al., 2018; Ingram Katherine et al., 2019; Asanjarani and Arslan, 2020; Cardeli et al., 2020; Espelage et al., 2020; Ellinger et al., 2022). In three studies (Acosta et al., 2019; Wagle et al., 2021; Booker et al., 2022), McDonald's Omega was reported for the PSSM and the SCS and was found to be good (0.81–0.96).

The types of validity that were assessed in these frequently used measures included measurement invariance across time points, sex, age, grade, and poverty quantile groupings, construct, content, convergent, discriminant, predictive, concurrent, face, content, and cross-cultural validity (Supplementary Material 2). Cross-cultural validity was assessed in only two studies (Abubakar et al., 2016; Hussain Shadab et al., 2018) that established the measurement invariance of the PSSM across countries or cultural groups (see Supplementary Material 2 for fit indices).

All these measures were used across different countries and generally appear to be reliable and valid (where reported) outside of their countries of origin except the PSSM in two studies (Lam Un et al., 2015; Cowden Richard et al., 2018), the SCS in two studies (Sun et al., 2013; Thumann Barbara et al., 2016), and the School Belongingness Scale in one study (Asanjarani and Arslan, 2020). These studies reported poor reliabilities (Cronbach's alpha <0.70) of these measures when used outside of their countries of origin (Supplementary Material 2).

### Measures of Family Connectedness Among Adolescents and Their Psychometric Properties

Seventy measures of adolescent family connectedness were used across 122 of the included studies (See Supplementary Material 4B for the full list of these measures including their frequency of use). Among these, the Parent-Family Connectedness Scale (Resnick et al., 1997), the Parental Bonding Instrument (Parker et al., 1979), the Multigenerational Interconnectedness Scale [MIS] (Gavazzi and Sabatelli, 1987), the Family Adaptability and Cohesion Evaluation Scale [FACES-II] (Olson et al., 1982), and the Social Connectedness Scale (Lee and Robbins, 1995) were the most frequently used measures. Forty-nine of the included studies used these five measures and their psychometric properties were reported in 40 studies and are summarized in Table 3. Detailed psychometric properties of all the measures of family connectedness are in Supplementary Material 2.

**Table 3:** Psychometric properties of the most frequently used measures of adolescent family connectedness.

**Measure used**	**Number of studies**	**Reliability**	**Validity**		
		**Internal consistency**	**Construct validity**	**Convergent validity**	**Divergent validity**
Parent-Family Connectedness Scale	24	-Cronbach's alpha reported in 19 studies (range = 0.73–0.97)	Established in one study	NR	NR
Multigenerational Interconnectedness Scale (MIS)	6	-Cronbach's alpha reported in all the six studies (range = 0.60–0.98) -Five (83.3%) studies reported Cronbach's alpha > 0.70	Established in three studies	Established in two studies	Established in one study
Family Adaptability and Cohesion Evaluation Scale (FACES-II)	7	-Cronbach's alpha reported in all the seven studies (range = 0.78–0.93)	NR	NR	NR
Parental Bonding Instrument	6	-Cronbach's alpha reported in three studies (range = 0.36–0.93) -Two (66.7%) studies reported Cronbach's alpha > 0.70	NR	NR	NR
Social Connectedness Scale	6	-Cronbach's alpha reported in five studies (range = 0.38–0.90) -Four (80.0%) studies reported Cronbach's alpha > 0.70	NR	NR	NR

*NR, not reported*.

Across studies, the reported reliability was acceptable (Cronbach's alpha ≥ 0.70) except in only three studies (Dwairy and Menshar, 2006; Carlo et al., 2011; Law et al., 2013) that respectively reported a Cronbach's alpha of < 0.70 for the Social Connectedness Scale, the MIS, and the Parental Bonding Instrument. Of the five measures, validity was reported only for the Parent-Family Connectedness Scale and the MIS in only five studies (Dwairy, 2003; Dwairy and Menshar, 2006; Dwairy et al., 2006; Dwairy and Dor, 2009; Mueller Christian et al., 2011). The only reported and established types of validity in these studies were construct, convergent, and divergent validity.

All the measures except the Social Connectedness Scale were used across multiple countries. Where the psychometric properties were reported, these measures still remained reliable and/or valid for use outside of their countries of origin in all the studies except in only two studies (Dwairy and Menshar, 2006; Law et al., 2013) where the MIS and the Parental Bonding Instrument had poor reliability.

### Measures of Community Connectedness Among Adolescents and Their Psychometric Properties

In total, 40 measures of adolescent community connectedness were used (see Supplementary Material 4C for the full list of these measures including their frequency of use). Of these, the Sense of Belonging Instrument—Psychological Subscale [SOBI-P] (Hagerty and Patusky, 1995), the Community Connectedness Scale (Fletcher and Shaw, 2000), the Sense of Community Index (Chavis et al., 1986), the Sense of Community Scale for Adolescents [SOC-A] (Cicognani et al., 2008), the Neighborhood Youth Inventory [NYI] (Chipuer et al., 1999), the Neighborhood Intergenerational Closure Scale (Sampson et al., 1999), and the Collective Efficacy Scale [CES] (Sampson et al., 1997) were the most frequently used measures used in 19 of the included studies. Table 4 presents a summary of the psychometric properties of these measures. Detailed psychometric properties of all the measures of community connectedness are in Supplementary Material 2.

**Table 4:** Psychometric properties of the most frequently used measures of adolescent community connectedness.

**Measure used**	**Number of studies**	**Reliability**	**Validity**
		**Internal consistency**	**Construct validity**
The Sense of Belonging Instrument – Psychological Subscale (SOBI-P)	5	-Cronbach's alpha reported in all the five studies (range = 0.86–0.95)	NR
Community Connectedness Scale	3	-Cronbach's alpha reported in all the three studies (range = 0.70–0.75)	NR
Sense of Community Index	2	-Cronbach's alpha reported in both studies (0.71 and 0.74)	NR
Sense of Community Scale for Adolescents (SOC-A)	2	-Cronbach's alpha reported in both studies (0.82 and 0.93)	Established in one study
Neighborhood Youth Inventory (NYI)	3	-Cronbach's alpha reported in two studies (range = 0.44–0.93 across subscales) -One of the studies reported Cronbach's alpha <0.70 for some subscales	NR
Neighborhood Intergenerational Closure Scale	2	-Cronbach's alpha reported in both studies (0.78 in each study)	
Collective Efficacy Scale (CES)	2	-Cronbach's alpha reported in both studies (0.72 and 0.76)	NR

*NR, not reported*.

In summary, the psychometric properties of these measures were reported across 18 studies. All the 18 studies reported reliability (internal consistency) with only one of these studies (Albanesi et al., 2007) additionally reporting the validity of the SOC-A. The reported Cronbach alpha was above 0.70 in all the studies except in one study (Chipuer et al., 2003) that reported lower Cronbach alphas for some subscales in the NYI. Only construct validity was reported for the SOC-A where confirmatory factor analysis derived a five-factor solution that accounted for 53.8% of the total variance. Among these measures, only the SOBI-P and the Sense of Community Index were used in more than one country and showed good reliability across the countries in which they were used (Cronbach's alpha ranging from 0.71 to 0.95).

### Measures of Ethnic Identity/Connectedness Among Adolescents and Their Psychometric Properties

Only five measures were used across 48 of the included studies to assess ethnic identity in adolescents. These measures include the MEIM (Phinney, 1992), the Ethnic Identity Scale (Umaña-Taylor et al., 2004), the Ethnic and Moral Identity Scale [EMIS] (Aldridge Jill et al., 2016), the Multidimensional Inventory of Black Identity–Teen [MIBI-T] (Scottham et al., 2008), and the Psychological Acculturation Scale (Tropp et al., 1999). The psychometric properties of these measures were reported in 40 of these studies with all these studies reporting their reliability and five of them additionally reporting their validity (Table 5).

**Table 5:** Psychometric properties of measures of adolescent ethnic identity/connectedness.

**Measure used**	**Number of studies**	**Reliability**	**Validity**	
		**Internal consistency**	**Construct validity**	**Measurement invariance**
Multigroup Ethnic Identity Measure (MEIM)	40	-Cronbach's alpha reported in 34 studies (range = 0.63–0.96) -One study additionally reported McDonald's Omega (0.89) -28 (82.4%) studies reported Cronbach's alpha > 0.70	Established in five studies	-Longitudinal measurement invariance established in one study -Measurement invariance across cultural groups established in two studies
Ethnic Identity Scale	3	-Cronbach's alpha reported in two studies (range = 0.84–0.89)	NR	NR
Ethnic and Moral Identity Scale (EMIS)	2	-Cronbach's alpha reported in only one study (0.91 and 0.94 for the subscales)	Established in one study	NR
Multidimensional Inventory of Black Identity–Teen (MIBI-T)	2	-Cronbach's alpha reported in the two studies (0.67 and 0.87) -One of these studies reported Cronbach's alpha < 0.70	NR	NR
Psychological Acculturation Scale	1	-Cronbach's alpha of 0.89–0.91 across groups	NR	NR

*NR, not reported*.

The reported internal consistency was adequate (Cronbach's alpha ≥ 0.70) in all but seven of these studies (Phinney, 1992; Wallace Scyatta and Fisher Celia, 2007; Wills et al., 2007; Dandy et al., 2008; Kiang and Fuligni, 2009; Galliher et al., 2011; Tabbah et al., 2016). One study (Caqueo-Urizar et al., 2021) additionally reported McDonald's Omega of the MEIM (0.89). Validity was only reported for the MEIM and the EMIS. The types of validity assessed for these two measures included longitudinal measurement invariance, construct, and cross-cultural validity. The cross-cultural validity was established for the MEIM in two studies (Spencer et al., 2000; Caqueo-Urizar et al., 2021) where Multigroup equivalence across different cultural groups was established.

Among these measures, only the MEIM was used across countries (Table 5). Among the studies that used this measure and reported its psychometric properties, only six studies (Phinney, 1992; Wallace Scyatta and Fisher Celia, 2007; Dandy et al., 2008; Kiang and Fuligni, 2009; Galliher et al., 2011; Tabbah et al., 2016) reported poor reliability (Cronbach's alpha <0.70). From these studies, only one of them (Dandy et al., 2008) was conducted outside the measure's country of origin (the United States).

### Measures of Racial Identity/Connectedness Among Adolescents and Their Psychometric Properties

Only three measures were used across three of the included studies (DeCarlo, 2005; Sellers Robert et al., 2006; Mandara et al., 2009) to assess racial identity among adolescents. The measures include the Racial Identity Attitude Scale (King et al., 2015), the MIBI-T (Scottham et al., 2008), and the MEIM (Phinney, 1992) (Supplementary Material 2). Of these measures, the only reported psychometric properties was reliability (internal consistency) which was reported for the MIBI-T and the MEIM. The reported internal consistency of the MEIM was good (Cronbach's alpha of 0.80 and 0.73 across groups). The reported Cronbach's alpha was below 0.70 in some subscales of the MIBI-T.

### Measures of Cultural Connectedness Among Adolescents and Their Psychometric Properties

Nine measures, including the MEIM (Phinney, 1992), the Cultural Connectedness Scale [CCS] (Snowshoe et al., 2015), the Cultural Connectivity Scale–California [CCS-CA] (King et al., 2019), the Alaska Native Cultural Identification [ANCI] (Wolsko et al., 2009), the Hawaiian Culture Scale (Hishinuma et al., 2000), the Navajo Cultural Identity Measure (Rieckmann Traci et al., 2004), the Residence Culture Identity Measure [RCIM] (Weber et al., 2015), the Acculturation, Habits, and Interests Multicultural Scale for Adolescents [AHIMSA] (Unger et al., 2002), and a new 6-item Cultural Connectedness Measure adapted from the MEIM (Hilario Carla et al., 2014), were used across 12 of the included studies to assesses adolescents' cultural connectedness. Of these measures, only the MEIM, the RCIM and the CCS were each used in two studies. The rest of the measures were each used in only one study (Supplementary Material 2).

Of the 12 studies that used these measures, eight of them reported the internal consistency of some of these measures with three studies (Rieckmann Traci et al., 2004; Snowshoe et al., 2015, 2017) additionally reporting their validity. One study (King et al., 2019) only reported validity, reporting the face validity of the CCS-CA. The reported internal consistency was adequate (Cronbach's alpha ≥ 0.70) in all but one study (Mastrotheodoros et al., 2021) that reported poor reliability for the MEIM in some time points. The reported and established types of validity in the three studies include construct, convergent, content, and face validity.

### Measures of Religious/Spiritual Connectedness Among Adolescents and Their Psychometric Properties

Only seven measures were used across eight of the included studies to measure religious/spiritual connectedness among adolescents. These measures include the Multi-Religion Identity Measure [MRIM] (Abu-Rayya et al., 2009), the Religious/Spiritual Connectedness Scale (Resnick et al., 1993), the Spiritual Connectedness Scale (Holder et al., 2000), the Religious Collective Self-Esteem Scale [RCSES] (Oulali et al., 2019), the Brief Multidimensional Measure of Religiousness/Spirituality [BMMRS] (Group., 1999), the Personal Experience Inventory (Winters et al., 1989), and a newly developed 6-item Scale (Abubakar et al., 2014). Among these measures, only the MRIM was used across two studies. The remaining measures were each used only in one study (Supplementary Material 2).

The psychometric properties of these measures were reported across six studies (Abu-Rayya et al., 2009; Abubakar et al., 2014; Abu-Rayya Maram et al., 2016; Wright Anna et al., 2018; Oulali et al., 2019; Rose et al., 2019). The six studies all reported Cronbach alpha's of above 0.70 except in one study (Oulali et al., 2019) that reported lower Cronbach's alphas for some subscales in the RCSES. Only one study (Oulali et al., 2019) additionally reported the test-retest reliability of the RCSES (0.36–0.58). Three studies (Abu-Rayya et al., 2009; Abubakar et al., 2014; Oulali et al., 2019) additionally reported the validity of the MRIM, the RCSES, and the new 6-item scale. The reported and established types of validity in these studies include measurement invariance across religious groups, construct, convergent, discriminant, predictive, and incremental validity.

### Measures of Peer Connectedness Among Adolescents and Their Psychometric Properties

Supplementary Material 4D presents the measures of adolescent peer connectedness including their frequency of use. In summary, 54 individual measures were used to assess peer connectedness. Of these measures, the HMAC (Karcher, 2001), the BPNS (Ryan and Deci, 2000), the Need for Relatedness Scale (Richer and Vallerand, 1998), the Peer Connectedness Measure from the Add Health Study (Sieving et al., 2001), the Basic Psychological Needs in Exercise Scale (Vlachopoulos and Michailidou, 2006), the Peer Motivational Climate in Youth Sport Questionnaire [peerMCYSQ] (Ntoumanis and Vazou, 2005), and the Relational Provision Loneliness Questionnaire [RPLQ] (Hayden-Thomson, 1989) were the most frequently used measures. Of the 19 studies that used these measures, 18 studies reported their reliability with three studies (Quested et al., 2010; López et al., 2012; Girelli et al., 2019) additionally reporting the validity of the BPNS, the Need for Relatedness Scale, and the peer MCYSQ (Table 6).

**Table 6:** Psychometric properties of the most frequently used measures of adolescent peer connectedness.

**Measure used**	**Number of studies**	**Reliability**	**Validity**			
		**Internal consistency**	**Construct validity**	**Content validity**	**Criterion validity**	**Measurement invariance**
Hemingway's Measure of Adolescent Connectedness (HMAC)	6	-Cronbach's alpha reported in all the six studies (range = 0.72–0.84)	NR	NR	NR	NR
Basic Psychological Needs Satisfaction Scale (BPNS)	5	-Cronbach's alpha reported in all the five studies (range = 0.74–0.85)	Established in one study	Established in one study	Established in one study	Longitudinal measurement invariance established in one study
Need for Relatedness Scale	6	-Cronbach's alpha reported in four studies (range = 0.87–0.90)	Established in three studies	NR	NR	NR
Peer Connectedness Measure from the Add Health Study	2	-Cronbach's alpha reported in both studies (range = 0.89–0.90)	NR	NR	NR	NR
Basic Psychological Needs in Exercise Scale	3	-Cronbach's alpha reported in all the three studies (0.62–0.92) -Two (66.7%) studies reported Cronbach's alpha > 0.70	NR	NR	NR	NR
Peer Motivational Climate in Youth Sport Questionnaire (peerMCYSQ)	2	-Cronbach's alpha reported in both studies (0.81 and 0.84)	Established in both studies	Established in one study	NR	NR
Relational Provision Loneliness Questionnaire (RPLQ)	2	-Cronbach's alpha reported in both studies (0.81 and 0.89)	NR	NR	NR	NR

*NR, not reported*.

The reported Cronbach's alphas were above 0.70 except in one study (Fin et al., 2019) that reported a Cronbach alpha of 0.62–0.92 for the Basic Psychological Needs in Exercise Scale. Test-retest reliability was additionally assessed only for the peer MCYSQ in only one study (Ntoumanis and Vazou, 2005) and was found to be adequate (0.77). The reported types of validity in the four studies that reported validity were measurement invariance across gender, criterion, content, and construct validity. All the types of validity were adequately established (see Supplementary Material 2). Detailed psychometric properties of all the measures of community connectedness are in Supplementary Material 2.

Although no study assessed the cross-cultural validity of any of these measures, the reported reliability and/or validity in the measures that were used across countries (the BPNS, the Need for Relatedness Scale, and the Basic Psychological Needs in Exercise Scale, the peer MCYSQ), and the RPLQ were all adequate except in only one study (Fin et al., 2019).

### Measures of Self-Connectedness Among Adolescents and Their Psychometric Properties

Only six measures were used in six studies (Moller Arlen et al., 2010; Piotrowski, 2013; Mann Michael et al., 2015; Opperman et al., 2015; Snowshoe et al., 2017; McCue et al., 2019) to assess self-connectedness in adolescents. The six measures were individually used once in each study and include the Identity subscale of the Adolescent Personality Style inventory (Lounsbury et al., 2005), the UCLA Loneliness Scale (Russell et al., 1978), the Dimensions of Identity Development Scale (Luyckx et al., 2008), the BPNS (Ryan and Deci, 2000), the HMAC (Karcher, 2001) and a new measure adapted from previous studies (McCue et al., 2019). Of these measures, internal consistency was reported for five of the measures except for the BPNS. The reported internal consistency was adequate (Cronbach's alpha ≥ 0.70) except for the new measure (McCue et al., 2019) and some subscales in the Dimensions of Identity Development Scale (Piotrowski, 2013). No study reported the validity of any of these measures.

### General Measures of Adolescent Connectedness and Their Psychometric Properties

In 154 studies, two or more domains of connectedness were assessed concurrently using one measure (Supplementary Material 2). In total, 63 such measures were used across the 154 studies (see Supplementary Material 4E for a list of these measures including their frequency of use). Of these measures, the Social Connectedness Scale (Lee and Robbins, 1995), the HMAC (Karcher, 2001), the EPOCH measure of adolescent wellbeing (Kern Margaret et al., 2016), the BPNS (Ryan and Deci, 2000), and the INQ (Van Orden et al., 2012) were the most frequently used measures. Eighty-one of the included studies used these five measures with 61 of them providing their psychometric properties. We have summarized their psychometric properties in Table 7. Detailed psychometric properties of all the general measures of adolescent connectedness are in Supplementary Material 2.

**Table 7:** Psychometric properties of the most frequently used general measures of adolescent connectedness.

**Measure used**	**Number of studies**	**Reliability**	**Validity**						
		**Internal consistency**	**Construct validity**	**Convergent validity**	**Concurrent validity**	**Discriminant validity**	**Face validity**	**Predictive validity**	**Measurement invariance**
Social Connectedness Scale	37	-Cronbach's alpha reported in 24 studies (range = 0.69–0.96) -23 (95.8%) studies reported Cronbach's alpha > 0.70	NR	NR	NR	NR	NR	NR	NR
Hemingway's Measure of Adolescent Connectedness (HMAC)	16	-Cronbach's alpha reported in 12 studies (range = 0.63–0.96) -Nine (75.0%) studies reported Cronbach's alpha > 0.70	Established in three studies	Established in one study	Established in one study	Established in one study	NR	NR	-Measurement invariance across gender established in one study -Measurement invariance across ethnic groups established in one study
Basic Psychological Needs Satisfaction Scale (BPNS)	15	-Reliability reported in 14 studies Cronbach's alpha reported in 13 studies (range = 0.62–0.90; one study reported composite reliability (0.81)] -Eleven (84.6%) studies reported Cronbach's alpha > 0.70	Established in two studies	Established in two studies	NR	Established in two studies	NR	NR	Measurement invariance across age groups established in one study
Interpersonal Needs Questionnaire (INQ)	7	-Cronbach's alpha reported in six studies (range = 0.79–0.94)	Established in two studies	Established in two studies	NR	NR	NR	NR	NR
Engagement, Perseverance, Optimism, Connectedness, and Happiness (EPOCH) Measure of Adolescent Wellbeing	6	-Reliability reported in five studies three studies reported Cronbach's alpha (range = 0.65–0.89); one study reported McDonald's Omega (0.94); one study reported composite reliability (0.83) -One study additionally reported McDonald's Omega (0.75) -Two (66.7%) studies reported Cronbach's alpha > 0.70	Established in four studies	Established in two studies	NR	NR	Established in one study	Established in one study	-Measurement invariance across gender established in two studies -Measurement invariance across age groups established in one study -Measurement invariance across countries established in two studies

*NR, not reported*.

Of the 61 studies reporting the psychometric properties of these measures, the reported internal consistency of these measures was adequate (Cronbach's alpha ≥ 0.70) across studies except in seven studies (Rew et al., 2001; McWhirter Benedict and McWhirter Ellen, 2011; Yuen and Yau, 2015; Kern Margaret et al., 2016; Sakan, 2020; Vandenkerckhove et al., 2021; Yuen and Datu, 2021). McDonald's Omega was reported for the EPOCH in two studies (Kern Margaret et al., 2019; Maurer et al., 2021) and was found to be adequate (0.75–0.94). In two studies (Holzer et al., 2021; Ingoglia et al., 2021), composite reliability was reported for the EPOCH and the BPNS, respectively. The reported reliabilities in these studies were 0.83 and 0.81, respectively. Test-retest reliability was additionally reported in only one study that used the EPOCH (Kern Margaret et al., 2016). The reported reliability in this study ranged from 0.36 to 0.55.

Eleven of these studies (Karcher and Sass, 2010; McWhirter Benedict and McWhirter Ellen, 2011; Kern Margaret et al., 2016, 2019; Podlogar et al., 2017; Roeder Kathryn and Cole David, 2019; Sakan, 2020; Holzer et al., 2021; Ingoglia et al., 2021; Maurer et al., 2021; Yuen and Datu, 2021) additionally reported the validity of some of these measures. The reported and established types of validity for these measures were measurement invariance across gender and age groups, construct, convergent, concurrent, predictive, discriminant, and cross-cultural validity. In assessing cross-cultural validity, only three of these studies (Karcher and Sass, 2010; Kern Margaret et al., 2019; Holzer et al., 2021) assessed and established the measurement invariance across cultures for the HMAC and the EPOCH (see Table 7 and Supplementary Material 2).

All these measures were used across different countries. Overall, these measures remained reliable and valid (where reported) outside of the countries of origin except the HMAC in three studies (McWhirter Benedict and McWhirter Ellen, 2011; Yuen and Yau, 2015; Yuen and Datu, 2021) and the BPNS in two studies (Sakan, 2020; Vandenkerckhove et al., 2021) that reported poor reliabilities. In two studies (Rew et al., 2001; Kern Margaret et al., 2016) that respectively reported poor reliabilities for the EPOCH and the Social Connectedness Scale, these studies were conducted in the countries in which these measures were developed (Supplementary Material 2).

## Discussion

We conducted this review to identify the existing measures of adolescent connectedness, the most frequently used measures among the identified measures, and to summarize the available evidence on the psychometric properties of the identified most frequently used measures. We identified 335 measures used to assess different domains of adolescent connectedness from 960 eligible studies. Most of the included studies (80.5%) were published from 2010 onwards. This re-affirms the growing interest in PYD over the recent years (Qi et al., 2020).

Most of the included studies (72.1%) were majorly from North America and Europe. However, since we only included studies that were published in English, this precludes any conclusions on the distribution of the included studies as well as the dearth of measures of adolescent connectedness in other regions of the world that do not commonly use English.

Most of the identified measures (39.4%, *n* = 132) were used to assess school connectedness in adolescents. This is consistent with evidence from this review that show that there has been more focus on adolescent school connectedness compared to the other domains of adolescent connectedness since most of the included studies assessed school connectedness. It is possible that there has been more focus on adolescent school connectedness because adolescents spend most of their time at school or engaging in school activities (Morton et al., 2016; Zhao et al., 2021). On the other hand, there were scanty measures of ethnic, racial, cultural, religious/spiritual, and self-connectedness. This highlights a need for more development and/or adaptation of measures of these constructs. The lack of emphasis on some of these domains may also reflect the cultural context in which studies of connectedness have so far been carried out. While many countries within LMICs fall under what has been described as “collectivistic cultures” and are highly spiritual, most of the countries in the HICs groups are the opposite (Cohen et al., 2016). Consequently, it may be anticipated that there will be less emphasis on aspects such as religious/ spiritual connectedness and community connectedness since these domains play a less prominent role in their lives.

From the identified measures, we found a total of 60 measures that were frequently used across the included studies to assess the various domains of adolescent connectedness (see subtopics in the results section). These measures were used in more than half of the included studies (50.1%, *n* = 481). The preferred usage of these measures may be attributed to the evidence from this review that show the existence of more reliability and/or validity data of these measures compared to other measures. In this review, of the 756 studies that assessed any aspect of connectedness and reported reliability and/or validity, more than half of the reports (52.9%, *n* = 400) were for these frequently used measures.

Most of the included studies that used the most frequently used measures of adolescent connectedness provided their psychometric properties. Of the 481 included studies that used these measures, 83.2% (*n* = 400) of them reported an aspect of reliability and/or validity of these measures. From this review, these most frequently used measures appear to be reliable. Of the 400 studies that reported the psychometric properties of these measures, the reported internal consistency ranged from good to excellent (Cronbach's alpha ≥ 0.70) (Cicchetti, 1994) in 89.8% (*n* = 359) of the studies. However, most of the included studies used Cronbach's alpha instead of McDonald's omega to report internal consistency. This is despite the fact that Cronbach's alpha underestimates the true reliability unless tau-equivalence is achieved for the items (Deng and Chan, 2016). Future studies should strive to assess reliability more using McDonald's omega. Notably, very few studies (*n* = 3) (Ntoumanis and Vazou, 2005; Kern Margaret et al., 2016; Oulali et al., 2019) reported the test-rest reliability of these measures. Future research should strive to also assess the test-retest reliability of these measures to ascertain their temporal stability. This is especially important if one wants to use these measures in pre-post studies.

These measures also appear to be valid in terms of their measurement invariance, construct, convergent, discriminant, concurrent, face, predictive, content, and criterion validity. Importantly, some of these measures–specifically–the PSSM, the HMAC, the MEIM, and the EPOCH measure of adolescent wellbeing appear to be valid for use across cultures and countries. Seven of the included studies (Spencer et al., 2000; Karcher and Sass, 2010; Abubakar et al., 2016; Hussain Shadab et al., 2018; Kern Margaret et al., 2019; Caqueo-Urizar et al., 2021; Holzer et al., 2021) assessed and demonstrated the cross-cultural validity of these measures. Although limited to only a few studies, this is a great first step in ascertaining that these measures are valid for use in different contexts. A step forward would be more cross-cultural validation of these measures.

Most of these measures have also been used across several countries. In most cases, these measures retained adequate psychometric properties even when used outside of their countries of origin. While this provides a cause for optimism, caution must be exercised when interpreting this finding as this might be limited to the Western settings given the low number of the included studies from regions such as South America and Africa. Furthermore, the non-western studies might have initiated their studies with an etic assumption. Based on this, we have two recommendations. First, every study using any of these measures should try as much as possible to ascertain their psychometric properties. This is especially important given that it is erroneous to conclude that measures are reliable and/or valid in a given setting based on reliability and/or validity evidence from previous studies, a phenomenon called induction of reliability and/or validity (Sánchez-Meca et al., 2011; Merino-Soto and Angulo-Ramos, 2021). This is because tool performance may vary from one study population to another. Second, we need more studies that start from an emic perspective by looking at the cultural adequacy of these constructs.

In our review, we also observed that most of the measures carried out very limited cultural adaptations. Our observations indicated that authors largely translated and back translated the measures without looking out for other important aspects in ensuring cultural adequacy of the measures (van de Vijver and Tanzer, 2004; Abubakar and Van De Vijver, 2017). The lack of detailed evaluation of the extent to which we avoid construct, item and methods bias limits our ability to fully recommend some of the measures for use across different contexts. Further intensive cross-cultural validation of both constructs and items are recommended.

In conclusion, the frequently used measures of adolescent connectedness identified in this review appear to be reliable and valid. However, the reliability and validity evidence of these measures originate from North America and Europe. This reflects a limitation of our review where we only considered studies published in English. It might also be a reflection of the paucity of measures of adolescent connectedness in other regions. Future studies, perhaps a review of this nature without language limitation, are required for clearer insights on whether this observation is due to language bias or the lack of research in other regions of the world.

### Limitations of the Review

This review had a limitation worth highlighting. Our eligibility criteria were for studies that have been published in the English language. Therefore, it is possible that we may have left out important work published in other languages.

## Conclusions

Several measures assessing adolescent connectedness exist. These measures assess various domains of adolescent connectedness. Among these measures, 60 of them met our criteria for the most frequently used measures. These frequently used measures appear to be reliable and valid measures. However, the evidence of reliability and validity mostly originate from the North American and European contexts. This reflects a limitation of this review which only considered studies published in English. It might also reflect a paucity of research in other regions of the world. Further research is needed for clearer insights.

## Data Availability Statement

The original contributions presented in the study are included in the article/Supplementary Material, further inquiries can be directed to the corresponding author.

## Author Contributions

AA conceptualized the study. ET, EC, AM, and AA developed and refined the search strategy. ET, EC, and AM conducted the database search and screened and extracted data from all included articles. ET wrote the first draft of the manuscript. EC, AM, and AA all critically reviewed the first draft and subsequent revisions of the manuscripts. All authors contributed to the article and approved the initial submitted version. Due to the passing of AM, authors ET, EC, and AA read and approved the final submitted version of the manuscript.

## Funding

This research was funded by the Templeton World Charity Foundation award grant (Grant ID: TWCF0506) to AA. The funders had no role in the study's design, in the collection, analyses, or interpretation of data, in the writing of the manuscript, or in the decision to publish the results.

## Conflict of Interest

The authors declare that the research was conducted in the absence of any commercial or financial relationships that could be construed as a potential conflict of interest.

## Publisher's Note

All claims expressed in this article are solely those of the authors and do not necessarily represent those of their affiliated organizations, or those of the publisher, the editors and the reviewers. Any product that may be evaluated in this article, or claim that may be made by its manufacturer, is not guaranteed or endorsed by the publisher.
