# Supplementary material for: Adolescent Connectedness: A Scoping Review of Available Measures and Their Psychometric Properties
Source: Front Psychol. 2022 May 18;13:856621. doi: 10.3389/fpsyg.2022.856621 (PMC9159472; doi:10.3389/fpsyg.2022.856621)
Supplement: Supplementary file 1 [file Data_Sheet_1.zip › Supplementary File 1-Search Strategy.docx]

**Supplementary File 1: Search Strategy**

| **Key word** | **Synonyms** |
| --- | --- |
| Connectedness | Social connect* OR interconnectedness OR relatedness OR interrelatedness OR social relation* OR interrelationship OR kinship* OR social association* OR social bond* OR belonging |
| **AND** |  |
| Adolescen* | Child* OR young people OR teen* OR youth* OR child OR children OR childhood |
| **AND** |  |
| Measure* | Tool* OR test* OR scale OR assessment OR questionnaire OR interview |
