# Supplementary material for: Adolescent Connectedness: A Scoping Review of Available Measures and Their Psychometric Properties
Source: Front Psychol. 2022 May 18;13:856621. doi: 10.3389/fpsyg.2022.856621 (PMC9159472; doi:10.3389/fpsyg.2022.856621)
Supplement: Supplementary file 1 [file Data_Sheet_1.zip › Supplementary File 2-Characteristices of Included Studies.docx]

**Supplementary File 2: Characteristics of the Included Studies**

| **Study characteristics** | | | | | **Type of connectedness/ relatedness** | **Measures used** | **Psychometric properties** | | | |
| --- | --- | --- | --- | --- | --- | --- | --- | --- | --- | --- |
| **Author (year)** | **Country** | **Study design** | **Population involved** | **Sample size**  **Age in years (range, mean (sd))**  **Sex** |  |  | **Reliability** | | **Validity** | |
|  |  |  |  |  |  |  | **Internal consistency** | **Test-retest /inter & intra-rater reliability** | **Validity assessed** | **Validity indices** |
| Arora (2018) | USA | Cohort | Children of Immigrants | 755  17.84 (0.80)  M & F | School connectedness | 6 items | NR | NR | NR | NR |
| Abdollahi (2018) | Iran | Cross-sectional | Students | 520  15-21 (17.2 (2.3))  M&F | School connectedness | PSSM | 0.92 | NR | Convergent validity  Discriminant validity  Construct validity  Cross-cultural adaptation | Average variance extracted of 0.73  Square root of average variance extracted did not exceed the correlations of study variables  Satisfactory model fit: SRMR of 0.069  Scale forward translated to Iranian and back translated to English. |
| Abu-Rayya (2006) | Israel | Cross-sectional | Mixed-ethnic Arab-European adolescents | 127  13-18  M & F | Ethnic identity | MEIM | 0.75 – 0.88 | NR | Cross-cultural adaptation | Scale forward translated to Arabic and back translated to English |
| Abubakar (2014) | Kenya | Cross-sectional | Adolescents of immigrant backgrounds | 149  12-19 (16.38 (1.45))  M&F | Ethnic identity  Religious identity  School connectedness | MEIM – ethnic belonging subscale  Newly developed 6-item scale  PSSM – 13-item school belonging subscale | 0.81  0.79  0.83 | NR  NR  NR | NR  Construct validity  Construct validity | NR  A single factor structure explaining 47.57% of the variance  A single factor structure accounting for 33% of the variance with factor loadings > 40 |
| Abubakar (2016) | Netherlands Kenya Indonesia  Spain | Validation | Students | 1928  12-18 (14.6 (1.5)) in Netherlands  11-20 (16.5 (1.4)) in Kenya  14-18 (15.7 (0.7)) in Indonesia  14-18 (15.1 (0.9)) in Spain  M&F | School connectedness | PSSM | 0 .84 in Netherlands  0 .73 in Kenya  0.82 in Indonesia  0.86 in Spain. | NR | Measurement invariance  Functional equivalence  Cross-cultural adaptation | A clustered item model showed excellent fit to the data across contexts: χ2 (12, *N* = 1,941) = 20.20, *p* < 0.001, χ2/*df* = 1.68, TLI = 0.98, CFI = 0.99, and RMSEA = 0.016  The relationship between the latent structure of the PSSM and life satisfaction scale was similar across contexts: χ2 (47, *N* = 1,941) = 125.06, *p* < 0.001, χ2/*df* = 2.66, TLI = 0.967, CFI = 0.973, ΔCFI = 0.006, and RMSEA = 0.029  Scale forward translated to target languages and back translated to English. A meeting was held to harmonize the translations. |
| Abubakar (2016) | Bulgaria | Cross-sectional | Bulgarian mainstreamers and Roma minority pupils | 264  10-18  M&F | Family connectedness  School connectedness  Community Connectedness  Peer connectedness | FACES-II  PSSM  Sense of community membership  7 items | NR  NR  NR  NR | NR  NR  NR  NR | NR  NR  NR  NR | NR  NR  NR  NR |
| Abu-Rayya (2016) | Australia | Cross-sectional | Muslim Students | 321  14-18 (15.6 (1.1))  M&F | Ethnic identity  Religious identity | MEIM  Multi-Religion Identity Measure (MRIM) | 0.92  0.90 | NR  NR | NR  NR | NR  NR |
| Abu-Rayya (2009) | Australia | Development and validation | Students | 457  17-19 (17.7 (0.36))  M&F | Religious identity | Multi-Religion Identity Measure (MRIM) | 0.83 for the overall scale  0.81 – 0.86 for the subscales | NR | Construct validity  Convergent validity  Predictive validity | Factor analysis yielded a one-factor solution accounting for 63% of the total variance  Significant positive correlations with measures of degree of religiousness (0.61 – 0.86), prayer practice (0.64 – 0.90), and attitudes towards Christianity/ Islam (0.71 – 0.92)  Correlations with all measures of well-being were significant and moderate, with absolute values in the range of 0.37 – 0.62 |
| Ackard (2006) | USA | Cross-sectional | Student | 4746  7^th^-11^th^ graders  M&F | Family Connectedness | 3 items | NR | NR | NR | NR |
| Ackard (2008) | USA | Cross-sectional | Sexually active Adolescents | 10,095  13-19 (16.7 (1.5))  M | Family Connectedness | 7 items | 0.86 | NR | NR | NR |
| Acosta (2019) | USA | Trial | Students | 2824 11&12  M&F | School connectedness | School connectedness scale | 0.81 (omega) | NR | NR | NR |
| Addae (2020) | Ghana | Cross-sectional | students | 2068  13-18  M&F | Family Connectedness | 4 items | 0.74 | NR | NR | NR |
| Adebiyi (2008) | Nigeria | Cross-sectional | Street children | 360  9-17 (16.2 (1.3))  M&F | Peer connectedness  Family connectedness | 5 items  5 items | NR  NR | NR  NR | NR  NR | NR  NR |
| Adibereshki (2020) | Iran | Cross-sectional | Students with hearing loss | 122  6^th^-9^th^ graders  M&F | Family and peer Connectedness | EPOCH measure of adolescents wellbeing | 0.75 | NR | NR | NR |
| Ahlborg (2019) | Sweden | Cross-sectional | Students | 7804  11-15  M&F | Family, school, and peer connectedness | Health Behaviour in School Children’s (HBSC) survey questionnaire | 0.84, 0.83, and 0.94 for family, school, and peer connectedness, respectively | NR | NR | NR |
| Ahmad (2014) | Malaysia | Cross-sectional | Students | 23645  12-17  M&F | Family Connectedness | 1 item | NR | NR | NR | NR |
| Akar (2013) | Turkey | Development and validation | Students | 781  10-15 (12.7 (0.9))  M&F | School Connectedness | Sense of Belonging to School Scale (SEBES) | 0.85 | NR | Construct validity  Convergent validity | -EFA suggested a 2-factor structure accounting for 60.72% of the total variance.  -High correlation between the scale and its subscales.  -Low to high correlations between subscales (*r* = 0.36 – 0.85, *p* < .01).  -High negative correlation between the scale scores and loneliness scores on the UCLA loneliness scale.  -High positive correlation between the scale’s scores and social Support. |
| Akiba (2010) | USA | Cohort | Students | 2787  15  M&F | School Connectedness | School connectedness scale | 0.82 | NR | NR | NR |
| Albanesi (2007) | Italy | Cross-sectional | Students | 566  14-19 (16.10 (1.20))  M & F | Community connectedness | Sense of Community Scale for adolescents (SOC-A) | 0.93 | NR | Construct validity | CFA derived 5 factors that accounted for 53.8% of the common variance |
| Albrecht (2002) | USA | Cross-sectional | Adolescents participating in a smoke cessation program | 53  14-19 (17.24 (1.63))  F | Family,  School and Community connectedness | Health Behavior questionnaire | 0.83, 0.86, and 0.89, respectively | NR | NR | NR |
| Aldridge (2016) | Australia | Development and validation | Students | 4067  12-17  M&F | School Connectedness  Ethnic Identity | What Is Happening in This School (WHITS) Questionnaire  Ethnic and Moral Identity Scale (EMIS) | 0.89 – 0.93 across subscales  0.91 and 0.94 for the subscales | NR  NR | Construct validity  Content validity  Face validity  Discriminant validity  Concurrent validity  Predictive validity  Construct validity | Factor analysis yielded a 6-factor structure accounting for 62.51% of the total variance  Established by basing items on the scale on sound theoretical grounds and extensive literature review  Students indicated that the items were clear, concise and easy to understand  Correlations between items in the scale were higher than the correlations with items from other scales  Each subscale differentiated significantly between schools  Significant negative correlations between the scale and subscales with bullying  2-factor structure accounting for 65.90% of the total variance |
| Aldridge (2016) | Australia | Cross-sectional | Students | 2122  12-17  M&F | School connectedness  Ethnic identity | What Is Happening in This School (WHITS) Questionnaire  Ethnic and Moral Identity Scale (EMIS) | NR  NR | NR  NR | NR  NR | NR  NR |
| Alivernini (2019) | Italy | Cross-sectional | Students | 36712  15.6 (0.8)  M&F | Peer relatedness | Classmate Social Isolation Questionnaire (CISQ-A) | 0.76 – 0.83 | NR | NR | NR |
| Allen (2020) | USA | Cross-sectional | Transgender, non-binary and gender nonconforming TNG/ LGBT | 287  18.2 (2.5) | School Connectedness | 6 items | NR | NR | NR | NR |
| Alloway (2013) | UK | Cross-sectional | Students | 104  15-17 (15.8 (0.5))  M&F | Peer, family, school, and community Connectedness | Social connectedness scale | NR | NR | NR | NR |
| Al‐Yagon (2016) | Israel | Cross-sectional | Typically developing adolescents | 356  12-15 (13.25 (0.70))  M & F | School connectedness | PSSM | 0.87 | NR | NR | NR |
| Anderman (2002) | USA | Cohort | Students | 58653  M&F | School Connectedness | School connectedness scale | 0.78 | NR | NR | NR |
| Anderson (2007) | USA | Cross-sectional | Youth  attending neighborhood youth centers | 1,757  13.0  M & F | Family connectedness | Multidimensional Scale of Perceived Social Support | 0.90 | NR | NR | NR |
| Anderson-Butcher (2012) | USA | Development and validation | Students | 870  7^th^-10^th^ graders  M & F | School connectedness | Perceived School Experiences Scale (PSES) – School Connectedness Subscale | 0.90 – 0.96 across samples for the overall scale, 0.85 – 0.95 for the school connectedness subscale | 0.89 for the overall scale, 0.84 for the school connectedness subscale | Predictive validity  Construct validity  Measurement invariance | As expected, the overall PSES, the School  Connectedness subscale, the Academic Motivation subscale, and the Academic Press subscale means positively and significantly correlated with participants’ perceptions of belonging (r = 0.58, r = 0.56, r = 0.46, r = 0.59, respectively) and perceptions of social competence (r = 0.33, r = 0.24, r = 0.33, r = 0.28, respectively)  Factor analysis suggested a 3-factor structure with good model fit: χ2 = 107.34, df = 74, p = 0.01, RMSEA = 0.034, CFI = 0.99, GFI = 0.95  Configural (χ2 = 180.26, df = 151,  p < 0.01, RMSEA = 0.032, CFI = 0.99,  GFI = 0.92) and complete (χ2 =  221.21, df = 179, p < 0.01, RMSEA = 0.036, CFI = 0.99, GFI = 0.90) gender invariance established |
| Ang (2016) | Malaysia | Cross-sectional | Students | 618  12-17 (14.8 (1.0))  M&F | Peer, family, school, and community Connectedness | HMAC | NR | NR | NR | NR |
| Anwar (2016) | Pakistan | Cross-sectional | Students | 225  16-19 (17.4 (1.0))  M&F | School connectedness | School connectedness scale | 0.75 | NR | Cross-cultural adaptation | Scale forward translated to Urdu and back translated to English |
| Arango (2016) | USA | Cross-sectional | Adolescents study | 321  12-15 (13.6)  M & F | School, family, and peer connectedness | UCLA Loneliness Scale-Revised | 0.80 | NR | NR | NR |
| Arango (2019) | USA | Trial | Adolescents | 142  12-15 (13.4 (1.1))  M&F | School connectedness  Family connectedness  Community Connectedness | School connectedness scale  Parent-Family connectedness scale  Community connectedness Scale | 0.85  0.90  0.70 | NR  NR  NR | NR  NR  NR | NR  NR  NR |
| Aronowitz (2004) | USA | Cross-sectional | Impoverished adolescents | 443  11-15  F | Family Connectedness | Parent-Family connectedness scale | NR | NR | NR | NR |
| Arslan (2017) | Turkey | Development & validation | Students | 562  11-19 (15.3 (2.0))  M&F | School connectedness | School belongingness scale  Student subjective wellbeing questionnaire (SSWQ) | 0.86 for the overall scale  0.83 & 0.85 for the subscales  0.77 – 0.82 for the sub-scales | NR | Construct validity  Convergent validity  NR | EFA and CFA produced a 2-factor structure with good model fit: χ2 = 65.389, df = 34, p = 0.001, SRMR= 0.046, RMSEA = 0.065, CFI = 0.97, TLI = 0.96  Expected correlation (r) with levels of school connectedness (0.6), joys of learning (0.5), educational purpose (0.4), academic efficacy (0.4), and subjective wellbeing (0.6)  NR |
| Arslan (2018) | Turkey | Cross-sectional | Students | 413  11-18 (13.9 (4.6))  M&F | School connectedness | School belongingness scale | NR | NR | Convergent validity | Expected correlation (r) with psychological wellbeing (0.6), positive affect (0.5), psychological distress (-0.4), and negative affect (-0.4) |
| Arslan (2018) | Turkey | Cross-sectional | Students | 825  14-19 (16.5 (1.2))  M & F | Peer, school, family, and community connectedness | Social connectedness scale and  General Belongingness Scale | 0.92 and 0.88 | NR | NR | NR |
| Arslan (2019) | Turkey | Cross-sectional | Students | 223  10-15 (12.1 (1.0))  M&F | School connectedness | School belongingness scale | 0.76 for the overall scale  0.70 & 0.73 for the subscales | NR | Construct validity  Measurement invariance | CFA confirmed a 2-factor structure with good model fit: (χ2= 40.38, df = 34, p = 20, CFI = 0.98, TLI = 0.97, RMSEA = 0.029)  Configural, metric and, scalar invariance provided adequate-to-good data model fit statistics for both gender and grade. |
| Arslan (2020) | Turkey | Cohort | Students | 402  10-15 (12.2 (1.0))  M&F | School connectedness | School belongingness scale | 0.78 for the overall scale | NR | NR | NR |
| Arslan (2019) | Turkey | Cross-sectional | Students | 244  14-18 (16.3 (1.0))  M&F | School Connectedness | School belongingness Scale | 0.87 | NR | NR | NR |
| Artiran (2020) | Turkey | Cross-sectional | Students | 158  13-18 (16.2 (1.4))  M&F | Family relatedness | Basic Psychological Needs Satisfaction and Frustration Scale | 0.63 for the relatedness subscale | NR | NR | NR |
| Arunachalam (2016) | Vietnam | Cross-sectional | General adolescent population | 4705  14-25 (19.1)  M | Family connectedness | 8 items | NR | NR | NR | NR |
| Arundell (2019) | USA | Cross-sectional | Students | 429  15.5 (1.6)  M&F | Peer, family, school, and community Connectedness | Social Connectedness Scale | 0.94 | NR | NR | NR |
| Asanjarani (2020) | Iran | Cross-sectional | Students | 676  12-18 (14.57 (1.34))  M & F | School connectedness | School Belongingness Scale | 0.61 | NR | NR | NR |
| Aspy (2012) | USA | Cross-sectional | Students | 1117  12-17 (14.3)  M&F | School connectedness | School connectedness score from the youth asset survey | 0.72 | 0.75 | NR | NR |
| Ataman (2019) | Turkey | Cross-sectional | Nursing students | 255  First years  M&F | Family Connectedness | Family sense of belonging scale | 0.82 | NR | NR | NR |
| Austin (2020) | USA | Trial | Students | 766  11-14 (12.3 (1.1))  M&F | Community connectedness | 1 item | NR | NR | NR | NR |
| Awaluddin (2019) | Malaysia | Cross-sectional | Students | 27455  13-17  M&F | Family Connectedness | 1 item | NR | NR | NR | NR |
| Azagba (2014) | Canada | Cross-sectional | Students | 8491  15.2 (0.1)  M&F | School Connectedness | 3 items | 0.74 | NR | NR | NR |
| Azzopardi (2020) | Indonesia | Cross-sectional | In-and out- of school adolescents | 1337 in-school  824 out- of school  16-18  M&F | Peer, family, school, and community connectedness  Family connectedness | Social connectedness scale – revised  Family attachment scale | NR  NR | NR  NR | NR  NR | NR  NR |
| Bao (2018) | China | Cohort | Students | 888  15.6  M&F | School Connectedness | Bao’s School Connectedness Scale | 0.89 | NR | NR | NR |
| Bao (2020) | China | Cross-sectional | Students | 1,010  15.0 (1.7)  M&F | School Connectedness | Bao’s School Connectedness Scale | 0.88 | NR | NR | NR |
| Barnert (2020) | USA | Cross-sectional | Youth returning home after incarceration | 50  15-19 (17)  M&F | Family connectedness | 3 items | NR | NR | NR | NR |
| Barrera-Osorio (2020) | Colombia | Cross-sectional | Students | 1486  12.9  M&F | School Connectedness | Pre-adolescent Civic Engagement Scale | NR | NR | NR | NR |
| Bartle-Haring (2012) | USA | Cohort | Adolescents | 500  10-14  M & F | Family connectedness | Social connectedness scale | 0.846 for mothers and 0.878 for fathers | NR | NR | NR |
| Basile (2018) | USA | Cohort | Students | 3549  12.8 (1.1)  M& F | School Connectedness | PSSM | 0.72 | NR | NR | NR |
| Batanova (2014) | USA | Cross-sectional | Middle school students | 581  10-14  M & F | School connectedness | School connectedness scale | 0.73 for girls and 0.71 for boys | NR | NR | NR |
| Bearinger (2005) | USA | Cohort | Students | 569  9-15 (11.9)  M&F | Peer, family, school, and community connectedness | Items from the Indian urban youth survey yielded sub-scales measuring these constructs after factor analysis | 0.73 for school connectedness  0.57 for peer, family, and community connectedness | NR | NR | NR |
| Bearinger (2010) | India | Cross-sectional | Adolescents | 569  9 -15 (11.9)  M & F | Family connectedness  School connectedness  Community connectedness | 10 items  6 items  6 items | 0.78  0.73  0.57 | NR  NR  NR | NR  NR  NR | NR  NR  NR |
| Beiswenger (2010) | England | Cross-sectional | Students from socioeconomically diverse backgrounds | 142  11 -14 years  12.4 (.59)  M & F | Peer relatedness | The Child Loneliness Scale | 0.86 and 0.79 across time points | NR | Construct validity | All item loadings were above 0.52 |
| BeLue (2009) | USA | Cross-sectional | General adolescent population | 35184  12-17 years  M&F | Community connectedness | 4 items | NR | NR | NR | NR |
| Benner (2008) | USA | Cross-sectional | Students | 1120  14.6  M&F | School Connectedness | Gottfredson’s effective school battery | 0.74 | NR | NR | NR |
| Bernstein (2019) | USA | Cross-sectional | Adolescents | 2,000  19.99 (4.95)  M & F | Family, school, peer, and community connectedness | Social connectedness Scale and Need to belong scale | NR | NR | NR | NR |
| Bersamin (2019) | USA | Cross-sectional | Students | 503  9-12^th^ grade  M & F | School connectedness | 9 items | 0.78 – 0.85 | NR | NR | NR |
| Beyens (2016) | Belgium | Cross-sectional | Students | 402  16.41 (1.43)  M & F | School connectedness | Need to Belong Scale | 0.83 | NR | Construct validity | CFI = 0.95, RMSEA = 0.05,  SRMR = 0.05 |
| Beyers (2003) | Belgium | Cross-sectional | Middle and late adolescent students | 975  16.3 (1.3) for middle adolescents  18.7 (1.5) for middle adolescents  M&F | Family connectedness | The differentiation in the family system scale  Emotional closeness and mutuality sub-scales of the relationship with mother/ father questionnaire  Connection and reciprocity sub-scales of the Mannheim individuation questionnaire  Trust and communication sub-scales of the inventory of parent and peer attachment | 0.79 for adolescent reciprocity and 0.88 for parent reciprocity  0.88 for emotional closeness and 0.72 for mutuality  0.78 for connection and 0.72 for reciprocity  0.89 for the two sub-scales | NR  NR  NR  NR | Cross-cultural adaptation | Scales independently translated to Dutch by the authors then a common version agreed upon following discussions |
| Bleil (2000) | USA | Cross-sectional | Adolescents with Asthma | 55  11.8  M&F | Family Relatedness | Relatedness Questionnaire | 0.89 | NR | NR | NR |
| Blum (2003) | Antigua  Bahamas  Barbados  British Virgin Islands  Dominica  Grenada  Guyana  Jamaica  St. Lucia | Cross-sectional | General adolescent population | 15695  10-18 years  M&F | Family connectedness | Scale derived from the Minnesota adolescent health survey and youth risk behavior survey | 0.80 | NR | NR | NR |
| Bluth (2016) | USA | Trial | General adolescent population | 34  14-17 years  M&F | Peer, family, school, and community connectedness | Social connectedness scale | 0.94 | NR | NR | NR |
| Bo Ram (2020) | USA | Cohort | Asian American girls | 243  12-19 (16 (1.6))  F | Family connectedness | 5 items | 0.87 | NR | NR | NR |
| Bolland 2016 | USA | Cohort | Adolescents | 6,562  12-17  M&F | School connectedness  Community Connectedness | School connectedness scale  Psychological Sense of community | 0.62  0.66 | NR | NR | NR |
| Bond (2007) | Australia | Cohort | Students | 2678  14.0  M&F | School, peer, family, and community Connectedness | Interview schedule for social interaction. | 0.87 | NR | NR | NR |
| Bonell (2017) | UK | Cross-sectional | Students | 140  11-12  M & F | School connectedness | Beyond Blue School Climate Questionnaire (BBSCQ) | 0.80, ordinal alpha= 0.83 | NR | NR | NR |
| Booth (2014) | USA | Cohort | Middle and high school students | 894  7-12^th^ grades  M & F | School connectedness | 5 item | 0.62 – 0.72 | NR | NR | NR |
| Borofsky (2013) | USA | Cohort | Adolescents | 118  12.7 (0.8)  M&F | School Connectedness | 7 items | 0.70 and 0.69 across time points | NR | NR | NR |
| Borowsky (1997) | USA | Cohort | Students | 71594  9^th^ and 12^th^ graders  M&F | Community connectedness | 4 items | 0.72 | NR | NR | NR |
| Borowsky (1999) | USA | Cohort | American Indian and native Alaska students | 11666  7^th^-12^th^ graders  M&F | Family connectedness | 5 items | 0.87 | NR | NR | NR |
| Borowsky (2002) | USA | Cohort | Adolescents with a history of grade retention | 13781  7^th^-12^th^ graders  M7F | Family connectedness  School connectedness | 12 items  6 items | 0.86 & 0.88 for mother and father, respectively  0.76 | NR  NR | NR  NR | NR  NR |
| Borraccino (2020) | Italy | Cross-sectional | Immigrant youth | 47,799  11, 13 & 15 years  M & F | School, peer and family connectedness | 2 items | 0.71 – 0.87 | NR | NR | NR |
| Bottiani (2017) | USA | Trial | Students | 19,726  15.9 (1.3)  M&F | School Connectedness | California Health Kids Survey | 0.81 | NR | NR | NR |
| Bottorff (2020) | Canada | Cross-sectional | Adolescents | 25  13-18  M & F | Family connectedness | 4 items | NR | NR | NR | NR |
| Bourgeois (2014) | Australia | Cross-Sectional | Students | 1343  11-18  M&F | Family, school, peer, and community Connectedness | Self in a Social context-Virtual connectedness subscale (SSC-VC) | 0.83 | NR | Construct validity | Factor analysis yielded a 3-factor that accounted for 50.5% of the total variance with eigenvalues > 1 |
| Boutelle (2009) | USA | Cohort | Students | 2516  12.8 (0.8)  M&F | Family connectedness | 4 items | 0.69 | 0.69 – 0.82 | NR | NR |
| Bower (2015) | Australia | Cohort | Students | 107  12-17  M&F | Peer and Family Connectedness | Contextualized assessment tool for risk and protection management (CATRPM) | 0.85 and 0.87 for peer and family connectedness, respectively | NR | NR | NR |
| Bower (2015) | USA | Trial | Students | 20  12-15 (13.2 (0.5))  M&F | School Connectedness | Child and Adolescent Social Support Scale | 0.92 – 0.97 | 0.75 – 0.78 | NR | NR |
| Bradley (2012) | Australia | Cross-sectional | Adolescents | 433  14.51 (1.70)  M & F | Family, school, peer, and community connectedness | Social Connectedness Scale–Revised | NR | NR | NR | NR |
| Bradshaw (2014) | USA | Validation | Students | 49928  16.0 (1.4)  M7F | School connectedness | School engagement sub-scale of the US Department of Education 3-factor model of school climate | 0.94 for the overall scale  0.82 – 0.87 for the subscales | NR | Construct validity  Measurement invariance | EFA revealed a 6-factor structure with good model fit. CFA confirmed the factor structure with adequate fit indices: CFI = 0.945, TLI = 0.934, RMSEA = 0.048, SRMR = 0.050  Configural and factorial invariance provided adequate-to-good data model fit statistics for gender, race, and grade |
| Braithwaite (2015) | USA | Cross-sectional | Adolescents | 330  12-17 (14.24)  M & F | Family connectedness | Social connectedness scale | 0.84 – 0.90 |  |  | NR |
| Brajša-Žganec (2005) | Croatia | Cross-sectional | Children exposed to different levels of war violence | 583  12-15 (13.67)  M & F | Family, school, peer, and community connectedness | Interpersonal Support Evaluation List (ISEL)- | 0.71 for belonging and acceptance subscale | NR | NR | NR |
| Brandseth (2019 | Norway | Cross- sectional | Students | 574  16-17  M & F | School connectedness | Teacher and Classmate Support Scale | 0.91 | NR | NR | NR |
| Breeman (2015) | Netherlands | Cross-sectional | Students with psychiatric disorders | 414  5-13 (10.1)  M&F | School connectedness | Closeness sub-scale of the Student-Teacher relationship scale | 0.88 | NR | NR | NR |
| Brooks (2018) | Canada  England  Scotland | Cross-sectional | Students | 26701  11-15years  M&F | Self, peer, family, school, and community connectedness | Fisher’s Spiritual Wellbeing scale | 0.86 in Canada  0.84 in England  0.83 in Scotland | NR | NR | NR |
| Brooks (2012) | UK | Cross-sectional | Students | 1255  15.0  M&F | School Connectedness | School sense of belonging | NR | NR | NR | NR |
| Brown (2000) | USA | Development and validation | Students | 1739  7^th^-12^th^ graders  M&F | School Connectedness | The School Connection Scale | 0.86 | NR | Content validity  Construct validity  Predicative validity | The initial pool of items was examined by teachers, university faculty and school district staff to ensure content validity. Minor revisions were made following this review  Factor analysis yielded a 3-factor structure explaining 49.5% of the total variance  Each subscale had a significant negative correlation with substance abuse as would be predicted from the literature and significant positive correlation with school participation and grades |
| Brown (2019) | USA | Cross-sectional | Adolescents | 855  9-13  M&F | Family Connectedness | 6 items | NR | NR | Content and face validity | Content and face validity of the questionnaire were established by an expert advisory panel. |
| Browne (2020) | USA | Cohort | Adolescent | 54  10-17 (14.1 (2.5))  M&F | Family Connectedness | 4 items | 0.89 and 0.92 across time points | 0.89 | Construct validity | PCA yielded a single factor that accounted for 74.21% of the total variance |
| Buckley (2009) | Australia | Cross-sectional | Students | 540  13.5  M&F | Family connectedness  School Connectedness | Parental bonding instrument  School as a caring community profile-II | 0.71 and 0.93 for father and mother bonding, respectively  0.71 | NR | NR | NR |
| Buckley (2020) | Australia | Trial | Students | 826  13.5 (0.5))  M&F | School Connectedness | 18 items | 0.89 | NR | NR | NR |
| Butler-Barnes (2018) | USA | Cohort | Black adolescent girls | 733  14.5 (1.6)  F | School connectedness | Inventory of School Climate-Student scale | 0.83 | NR | NR | NR |
| Byrd (2015) | USA | Cross-sectional | Black middle and high school students | 99  11 -18  15.21 (1.53)  M & F | School relatedness | Relatedness subscale of the Basic Needs Satisfaction scale | 0.86 |  | NR | NR |
| Caleon (2019) | Singapore | Cohort | Students | 787  Grade 7 and 8  M & F | School Relatedness | The Inventory of Teacher-Student Relatedness | 0.81 – 0.89 | 0.32 – 0.50 | NR | NR |
| Cardeli (2020) | USA | Cross-sectional | Refugee students | 34  11-15 (12.94 (1.07))  M & F | School connectedness | PSSM | 0.55 | NR | NR | NR |
| Carlo (2011) | USA | Cohort | Adolescents | 470  10-14  M & F | Family connectedness | Social Connectedness Scale | 0.38 – 0.88 | NR | NR | NR |
| Carney (2018) | USA | Cross-sectional | Students | 973  3^rd^-6^th^ graders  M&F | School connectedness | The Community and Youth Collaborative Institute-School Experiences Survey (CAYCI-SES) | 0.83 | NR | NR | NR |
| Caroll (2017) | Australia | Development and validation | Students | 1673  11-18 (13.9 (1.7))  M&F | Peer, family, school, and community connectedness | Self in a Social Context—Social Connectedness Scale | 0.95 for peer connectedness  0.91 for family connectedness  0.89 for school connectedness  0.87 for community connectedness | NR | Content validity  Construct validity | Professionals and adolescents judged the items as relevant and the language used as appropriate  All except two items loaded on the hypothesized factors; with most cross-loadings <0.1. |
| Carter (2019) | USA | Cross-sectional | Students | 530  18-25 (19.3 (1.5))  M&F | Ethnic identity | MEIM | 0.90 | NR | NR | NR |
| Cartera (2007) | New Zealand | Cross- sectional | Students | 652  14-17  M&F | School, Peer, and  Family Connectedness | Youth Risk Behavior Survey (YRBS) | NR | NR | NR | NR |
| Carvajal (2004) | USA | Cross-sectional | Adolescents | 2,004  12.3 (0.93)  M & F | School connectedness  Parent relatedness | 4 items  Parent relatedness scale | 0.75  0.89 | NR  NR | NR  NR | NR  NR |
| Cassidy (2009) | UK | Trial | Students | 461  11-15 (13.1 (1.3))  M&F | Peer connectedness | Social identity scale | 0.89 | NR | NR | NR |
| Casteli (2018) | Switzerland | Validation | Students | 1942  11.7  M&F | Family, school, Peer, and  Community Connectedness | Middle years development instrument (MDI) – Connectedness subscale | 0.68 – 0.85 across subscales | NR | Cross-cultural adaptation  Construct validity  Measurement invariance | Scale adapted to Swiss-Italian through forward- and back-translation  EFA extracted 14 factors that explained 62.80% of the variance. CFA of the Connectedness subscale demonstrated acceptable fit indices (χ2 (df) = 7.31, TLI = 0.94, CFI = 0.95, RMSEA = 0.06) for a 5-factor structure  Partial strong invariance across gender for the connectedness subscale was confirmed (χ2 =766.68, df = 184, CFI = 0.94, RMSEA = 0.042). Strict invariance across preadolescent groups for Italian native language and non-Italian native language was confirmed (χ2 =738.17, df = 215, CFI = 0.95, RMSEA = 0.035) |
| Cavicchiolo (2019) | Italy | Validation | Students | 36,712  15.6 (0.77)  M & F | Peer relatedness | Classmates Social Isolation Questionnaire for Adolescents (CSIQ-A) | 0.76 and 0.83 for subscales | NR | Construct validity  Measurement invariance  Criterion validity | Factor analysis established a 2-factor model with good model fit: CFI = 0.96; RMSEA = 0.06; SRMR = 0.04  Full scalar invariance established across immigrant background (∆CFI = 0.003), gender (∆CFI = 0.010) and socioeconomic status (∆CFI = 0.003)  Positive correlation of both subscales with positive affect (r = 0.40 and 0.25, respectively) |
| Celeste (2019) | Belgium | Cohort | Students | 3446  14.74 (1.18)  M & F | School connectedness | School engagement scale | 0.85 | NR | NR | NR |
| Cemalcilar (2009) | Turkey | Cohort | Volunteer adolescents | 505  12.3  M&F | Community connectedness | 5 items | 0.60 | NR | NR | NR |
| Cero (2018) | USA | Cross-sectional | Juvenile offenders | 590  16.6 (1.4)  M | Peer, family, school, and community Connectedness | Millon adolescent clinical inventory | NR | NR | NR | NR |
| Cervantes (2011) | USA | Trial | Latino Adolescents | 153  11-14  M | School and family Connectedness | SAMHSA  Government Performance and Results Act (GPRA) Participant Outcome Measures for  Discretionary Programs | 0.73 and 0.54 for school and family connectedness, respectively | NR | NR | NR |
| Chan (2013) | USA | Cohort | Students | 526  9-17 (11.2 (1.6))  M&F | School connectedness | HMAC | 0.64 at TI and 0.65 at T2 | NR | NR | NR |
| Chan (2013) | China | Cross-sectional | Macanese adolescents | 365  10-17  M | Family, school, peer, and community connectedness | Social Bonding Scale | 0.42 – 0.79 | NR | NR | NR |
| Chan (2015) | China | Cross-sectional | Students | 1880  11-19 (13.5 (1.2))  M&F | School connectedness | Teacher-Student Relationship subscale of the Quality of School Life Scale | 0.71 | NR | NR | NR |
| Chang (2013) | China | Cross-sectional | Students | 4011  15.78 (1.95)  M & F | School connectedness | PSSM | NR | NR | NR | NR |
| Chapman (2013) | Australia | Cross-sectional | Students | 572  13.6 (0.5)  M&F | School connectedness | PSSM | NR | NR | NR | NR |
| Chapman (2011) | Australia | Cross-sectional | Students | 509  13-15  M&F | School Connectedness | School as caring community profile II | 0.71 | NR | NR | NR |
| Chen (2012) | Canada | Cross-sectional | Students | 178  16-19 (16.20 (1.80))  M & F | Family, school, peer, and community connectedness | Social connectedness scale-revised | NR | NR | NR | NR |
| Chen (2013) | USA | Cross-sectional | students | 3,350  12.5  M&F | School Connectedness | 6 items from the school connectedness scale | 0.84 | NR | NR | NR |
| Chester (2019) | UK | Cross-sectional | Students | 4985  11-15  M&F | School, Family, and  Community Connectedness | HBSC questionnaire | 0.67 – 0.95 | NR | NR | NR |
| Chew (2016) | Singapore | Validation | Adolescent with epilepsy | 152  13-16  M&F | Family Connectedness | Family Resilience Assessment Scale | 0.92 – 0.97 | NR | Convergent validity  Construct validity | As hypothesized, there was a signiﬁcant positive relationship between the scale and self-esteem (r=0.58, p b 0.001)  EFA yielded a 7-factor structure that accounted for 83% of the total variance.  There was low to moderate correlations between two pairs of subscales: ‘ﬂexibility and connectedness’ and ‘collaborative problem-solving’; ‘resources–community’ and ‘resources–neighbors’. |
| Chiesi (2010) | Italy | Validation | Students | 661  15-18  M&F | Community connectedness | Brief scale of sense of community in adolescents | 0.74 – 0.88 for the sub-scales | 0.99, P < 0.001  0.93 – 0.96 for the sub-scales, P < 0.001 | Construct validity  Concurrent validity  Discriminant validity | CFA confirmed a 5-factor analysis with good model fit: TLI = 0.97, NFI = 0.97, CFI = 0.98, RMSEA = 0.06  Expected correlation (r) with perceived wellbeing and social support scales (correlation between sub-scales ranging from 0.08 – 0.35)  Significant differences in gender [F(1,648) = 16.12, P<0.001] and types of schools [F(1,648) = 10.36, P<0.001] |
| Chipuer (2003) | Australia | Cross-sectional | General adolescent population | 464  11-17  M&F | School connectedness  Community connectedness | PSSM  Neighborhood youth inventory (NYI) & neighborhood cohesion instrument (NCI) | 0.90 – 0.92  0.44 – 0.93 for subscales in NYI  0.68 – 0.88 for subscales in NCI | NR | NR | NR |
| Chiu (2016) | 41 countries^1^ | Cross-sectional | Students | 193,073  15-year olds  M&F | School connectedness | 6-item School belonging scale from the Programme for International Student Assessment (PISA) | 0.77 | NR | NR | NR |
| Cho (2005) | USA | Trial | Students | 1218  15.0  M&F | School and Peer  Connectedness | The high school questionnaire (HSQ) | 0.68 and 0.64 for school and peer connectedness, respectively | NR | NR | NR |
| Chu (2015) | Singapore | Cohort | Youth offenders | 168  13-18  M & F | Family connectedness | 13 items | 0.84 | NR | NR | NR |
| Chu (2017) | USA | Cross-sectional | Students | 469  18-42 (19.4 (2.0))  M&F | Family, school, peer, and community connectedness | Thwarted belongingness subscale of the Interpersonal needs questionnaire (INQ) | 0.90 | NR | NR | NR |
| Chularkan (2020) | Thailand | Cross-sectional | Adolescents living in children’s homes | 216  10-14 (12.4 (1.3))  M&F | Peer, family, school, and community connectedness | Social connectedness scale - revised | 0.80 | NR | NR | NR |
| Chun (2011) | USA | Cross-sectional | Hispanic adolescents | 478  11-14 (12.71 (.61))  M & F | School connectedness | PSSM | 0.88 | NR | NR | NR |
| Chun (2013) | USA | Cross-sectional | Students | 444  16-20 years  M&F | School connectedness | Commitment to learning subscale of the developmental assets profile | 0.79 | NR | NR | NR |
| Chung-Do (2015) | USA | Development and validation | Students | 717  9^th^-12^th^ graders  M&F | School connectedness | New 15-item School Connectedness Scale | 0.73 – 0.93 for the subscales | NR | Construct validity  Face validity | CFA confirmed a 5-factor structure with good model fit: χ2 = 368.75, df = 77, p<.0001, CFI = 0.992, TLI = 0.989, RMSEA = 0.073  Structural equation models confirmed face validity |
| Cikrikci (2017) | Turkey | Cross-sectional | Students | 140  14-18 (16.22 (0.86))  M & F | Family, school, peer, and, community connectedness | General belongingness scale | 0.79 | NR | NR | NR |
| Clark (2014) | New Zealand | Cross- sectional | Students | 2,059  12-18  M &F | School connectedness  Family Connectedness | 8-item School connectedness scale from a previous study  10-item Family connectedness scale from a previous study | 0.63  0.84 | NR | NR | NR |
| Clark (2020) | USA | Cross-sectional | Psychology students | 235  19.4 (2.1)  M&F | Peer, family, school, and community Connectedness | Motivation to remain friends questionnaire | 0.89 | NR | NR | NR |
| Clements-Nole (2019) | USA | Cohort | Adolescents | 111  12-17  M&F | School Connectedness | Youth asset survey | NR | NR | NR | NR |
| Cohen (2020) | Israel | Cross-sectional | Students | 651  12-15 (14.1 (1.3))  M&F | School relatedness | Basic psychological needs scale | 0.75 | NR | NR | NR |
| Cole-Lewis (2016) | USA | Cross-sectional | Students | 161  13.5 (1.1)  M&F | School connectedness  Family connectedness | School connectedness scale  Parent-family connectedness scale | 0.84  0.88 | NR | NR | NR |
| Collibee (2016) | USA | Cross-sectional | Students | 57  13-15 (14.1 (0.5))  M&F | Family relatedness | Adolescent Promoting and relatedness scale | 0.84 and 0.89 | NR | NR | NR |
| Conner (2016) | USA | Cohort | Children and Adolescents | 1482  14.3(1.8)  M&F | Family connectedness | 12 items | 0.74 for mother and 0.77 for father | NR | NR | NR |
| Connolly (2015) | Canada | Trial | Students | 509  11-14 (12.4 (0.6))  M&F | School Connectedness | PSSM | 0.86 | NR | NR | NR |
| Corrales (2016) | Australia | Cross-sectional | Adolescent in mentorship Programme | 254  18.9 (1.5)  M&F | Family, school, peer, and community Connectedness | The Sense of Belonging Instrument -Psychological Subscale (SOBI-P) | 0.93 | NR | NR | NR |
| Correa-Velez (2010) | Australia | Cohort | Youth with refugee backgrounds | 97  11-19 (15.1 (1.6))  M&F | Ethnic identity | MEIM | 0.81 | NR | NR | NR |
| Coskan (2016) | Belgium and Turkey | Cross-sectional | Students | 276  18.1 (2.1)  M&F | Family relatedness | Relatedness measure developed by Gungor and Phalet 2011 | 0.88 | NR | NR | NR |
| Cotier (2017) | China | Cross-sectional | Adolescents | 188  17.67 (1.93)  M & F | Family, school, peer, and community connectedness | Social Comparison Scale | 0.87 for belonging subscale | NR | NR | NR |
| Cowden (2018) | South Africa | Validation | Students | 1052  14.9 (1.7)  M&F | School connectedness | PSSM | 0.53 – 0.71 | NR | Construct validity  Measurement invariance  Discriminant validity  Concurrent validity | PCA extracted a 3-factor structure with good model fit: χ2 = 373.56, df = 87, P < 0.001, CFI = 0.915, SRMR = 0.043, RMSEA = 0.048  Scale showed good metric and Configural invariance across sex, age and poverty quantile groupings  Significant differences between sex across the 3 sub-scales; Significant differences between age groups in the inclusion subscale and between poverty quantile groupings in the belonging subscale  Inclusion and belonging subscales were significant predictors of substance use: χ2(3) = 38.75, p < 0.001 |
| Cox (2008) | USA | Cohort | Physical education students | 344  12.4 (0.7)  M&F | School Connectedness | PSSM | NR | NR | NR | NR |
| Craig (2014 | Canada | Trial | Multiethnic sexual minority youth | 263  13-20  M & F | Family, school, peer, and, community connectedness | Social connectedness scale | 0.80 | NR | NR | NR |
| Crespo (2010) | New Zealand | Cohort | Students | 1773  9-16 (12.12 (1.73))  M & F | School connectedness  Family connectedness | 8 items selected and adapted from the School Connectedness Scale and PSSM  2 items | 0.86 – 0.90  0.90 – 0.92. | NR  NR | NR  NR | NR  NR |
| Crocetti (2016) | Netherlands | Cohort | Students n | 497  13-18 (13.03 (0.46))  M & F | Family relatedness | Balanced relatedness scale | 0.85 – 0.89 | NR | NR | NR |
| Crouch (2014) | USA | Cross-sectional | Adolescents with disability | 133  11-15  M&F | School Connectedness | PSSM | 0.75 | NR | NR | NR |
| Cummins (2019) | USA | Cohort | Students | 7,052  9^th^ -12^th^ graders M&F | School Connectedness | California Health Kids Survey | NR | NR | NR | NR |
| Cunningham (2004) | Australia | Cohort | Middle Adolescents | 200  14-17  (15.33 (0.81))  M& F | School connectedness | Social Questionnaire for Secondary Students (SQSS) | 0.74 – 0.84 | NR | NR | NR |
| Cupito (2015) | USA | Cross-sectional | Latino adolescent | 191  14.0  M&F | School Connectedness | PSSM | 0.83 | NR | NR | NR |
| Curcio (2017) | Australia | Cross-sectional | Adolescents | 329  13-17  M & F | School Connectedness | School Connectedness Scale | 0.71 – 0.74 | NR | NR | NR |
| Czyz (2019) | USA | Cross-sectional | Adolescents with a history of suicidal attempt or ideation | 34  13-17 (15.5 (1.4))  M&F | Family, school, peer, and community connectedness | Interpersonal needs questionnaire (INQ) – thwarted belongingness subscale | NR | NR | NR | NR |
| Czyz (2012) | USA | Cohort | Psychiatrically hospitalized Adolescents | 338  13-17 (15.6 (1.3))  M&F | Peer and family Connectedness | Perceived Emotional / Personal Support Scale | 0.83 and 0.91 for family and peer connectedness, respectively | NR | NR | NR |
| Dandy (2008) | Australia | Validation | Adolescents from diverse ethnocultural groups | 485  10-15 (12.20 (1.36))  M & F | Ethnic identity | MEIM | 0.66 | NR | Construct validity | 2-factor structure supported on factor analysis: GFI=0.98, RMSEA= 0.05, CFI and TLI above 0.90 |
| Dang (2014) | USA | Cross-sectional | Maltreated homeless youth | 150  14-21 (18.0)  M&F | School connectedness  Family connectedness | School connectedness scale  Parent-family connectedness scale (5 items) | 0.79  0.90 | NR | NR | NR |
| Davidson (2010) | USA | Cross-sectional | Rural, lower- to middle-class, white youth | 383  3^rd^-7^th^ graders  M & F | School relatedness | Closeness subscale of the Student– Teacher Relationship Scale | 0.82 | NR | NR | NR |
| Davies (2020) | UK | Intervention | Disadvantaged and hard to reach youth | 99  19.0  M & F | Community connectedness | Inclusion of Community in the Self Scale | NR | 0.74 | NR | NR |
| Davis (2019) | USA | Cohort | students | 2,177  11-13 (12.3 (0.71))  M&F | School Connectedness | PSSM | 0.72 | NR | NR | NR |
| Dawes (2017) | USA | Cohort | Youth victimized by peers | 1360  11-12  M & F | School connectedness | PSSM | 0.82 | NR | NR | NR |
| Day (2016) | USA | Cross-sectional | Students | 337945  10-18 (15.05 (1.29))  M & F | School Connectedness | School Connectedness Scale | NR | NR | NR | NR |
| Day (2009) | USA | Cohort | Adolescents | 349  11-14 (11.2 (1.0))  M&F | Family Connectedness | Social connectedness scale | 0.85 and 0.88 for mother and father connectedness, respectively | NR | NR | NR |
| de Albuquerque (2012) | UK | Cohort | Adolescents | 88  13-23  (17.7 (2.36))  M & F | Family connectedness | Subjective Family Picture | 0.70 – 0.85 | 0.60 | NR | NR |
| De Luca (2012) | USA | Cross-sectional | Students | 1618  12-19 (15.0)  M&F | Peer connectedness  Family connectedness  School  connectedness | 2 items  3 items  1 item | NR  NR  NR | NR  NR  NR | NR  NR  NR | NR  NR  NR |
| De Luca (2012) | USA | Cross-sectional | Students | 663  9-12^th^ graders  M & F | School connectedness | School engagement scale | 0.63 | NR | NR | NR |
| De Lucca (2020) | USA | Cross-sectional | Adolescents | 4,898  15.0  M&F | Family Connectedness | National survey of Child Health | NR | NR | NR | NR |
| De Pedro (2017) | USA | Cross-sectional | Sexual minority adolescents | 21,953  7-11  M&F | School Connectedness | School Connectedness Scale | 0.80 | NR | NR | NR |
| DeBate (2016) | USA | Cross-sectional | Students | 384  3^rd^-5^th^ graders  M&F | Peer, family, and community connectedness | 6-item General connectedness scale for children | 0.56 | NR | NR | NR |
| Debnam (2014) | USA | Cross-sectional | Students | 19833  15.9 (1.4)  M&F | School connectedness | 10 items from various surveys | 0.88 | NR | NR | NR |
| DeCarlo (2005) | USA | Cross-sectional | African American adolescents | 110  14-16 (14.9)  M&F | Racial identity  Ethnic identity | Racial Identity Attitude Scale – Long Form  MEIM | NR | NR | NR | NR |
| DeCou (2019) | USA | Cross-sectional | Adolescents admitted to a public psychiatric hospital | 550  11-17  (14.60 (1.68)  M & F | Family, school, peer, and community relatedness | The Resiliency Scales for Children and Adolescents | 0.94 | NR | NR | NR |
| Delgado (2016) | USA | Cohort | Latino adolescents | 6782  10-19 (15.1 (1.7))  M&F | School connectedness | 2 items | 0.78 | NR | NR | NR |
| Demanet (2012) | Belgium | Cross-sectional | Students | 11872  15-17  M & F | School connectedness  Peer connectedness | PSSM  4 items | 0.80  0.74 & 0.75 | NR | NR | NR |
| Demirbas-Celik (2019) | Turkey | Cross-sectional | Students | 360  16.2  M & F | School relatedness | Basic Psychological Needs Scale (BPNS) | 0.73 | NR | NR | NR |
| Detrie (2007) | USA | Cross-sectional | LGB adolescent | 218  18.0  M&F | Family, school, peer, and community Connectedness | Social connectedness scale | 0.94 | NR | NR | NR |
| Devieux (2016) | USA | Cross-sectional | Haitian American adolescents | 276  14-17  M&F | School connectedness | School connectedness scale | NR | NR | NR | NR |
| Di Giacomo (2019) | Italy | Cross-sectional | General adolescent population | 1221  15-22 (18.3 (3.0))  M&F | Peer, family, school, and community connectedness | EPOCH measure of adolescent wellbeing | NR | NR | NR | NR |
| Ding (2020) | China | Cross-sectional | Chinese adolescents | 1529  14.74 (1.48)  M & F | School connectedness | School connectedness scale | 0.78 | NR | NR | NR |
| Dinnen (2020) | USA | Cross-sectional | Highly mobile students | 647  4^th^ and 5^th^ grade students | School Connectedness | The Community and Youth Collaborative Institute-School Experiences Survey (CAYCI-SES) | 0.74 | NR | NR | NR |
| Direcki (2020) | Turkey | Development and validation | Students | 855  Class 5-8  M&F | School Connectedness | School Belonging Scale | 0.92 | NR | Construct validity  Convergent validity  Discriminant validity | All the scale items had factor loading > the 0.55 cut-off point. Communalities of the items ≥ 0.35.  Average Variance Extracted of > 0.50 for the Tendency to School and Friendship Relations factors.  Average Variance Extracted values of the factors were higher than the MSV and ASV values. Also, the square root of average variance extracted scores of the factors were greater than the correlations between the factors. |
| Disckon (2016) | USA | Cross-sectional | Students | 748  11-14  (12.71 (0.059))  M & F | School connectedness | PSSM | 0.77 | NR | NR | NR |
| Dixson (2020) | USA | Cross-sectional | Students | 447  12-19 (15.8 (1.3))  M&F | School Connectedness | School Belonging Scale | 0.85 | NR | Construct validity | EFA yielded a 1-factor structure with factor loadings ranging between 0.759 and 0.903 |
| Donald (2002) | Australia | Cross-sectional | General adolescent population | 3082  15-24 (18.6 (2.8))  M&F | Family, school, peer, and community connectedness | 7 items | 0.48 for females, 0.46 for males | NR | NR | NR |
| Dooley (2015) | Ireland | Cross-sectional | Students | 3094  14.9 (1.6)  M&F | Peer and school connectedness | HMAC | 0.71 and 0.82 for peer and school connectedness, respectively | NR | NR | NR |
| Dost-Goskan (2020) | Turkey | Cross-sectional | General adolescents’ population | 1097  14-16 (15.12 (0.64))  M & F | Family relatedness | Basic Psychological Needs Scale | 0.81 and 0.82 | NR | Construct  validity | CFI = 0.99; TLI = 0.99; RMSEA = 0.01 for mother scale CFI = 0.99; TLI = 0.99; RMSEA = 0.02 for father scale |
| Dovi (2020) | USA | Cohort | Youth recently diagnosed with cancer and healthy controls | 146  8-17  M & F | School connectedness | HMAC | NR | NR | NR | NR |
| Duggins (2016) | USA | Cohort | Victims of school bullying | 373  13.59 (1.22)  M & F | School connectedness | School connectedness school | 0.91 | NR | NR | NR |
| Duke (2009) | USA | Cohort | Comprised of youth from the core in-home interview sample | 9130  15.4 (1.79)  M & F | School connectedness  Family connectedness  Community connectedness  Peer connectedness | 8 items  11 items  6 items  1 item | 0.88  0.79  0.61  NR | NR  NR  NR  NR | NR  NR  NR  NR | NR  NR  NR  NR |
| Duke (2020) | USA | Cross-sectional | Students | 81,885  13-19 (15.5)  M&F | School connectedness | 7 items | 0.84 | NR | NR | NR |
| Duru (2008) | Turkey | Cross-sectional | Students | 404  16-27 (18.7)  M&F | Peer, family, school, and community connectedness | Social connectedness scale | NR | NR | NR | NR |
| Dwairy (2003) | Israel | Validation | Arab students | 518  10^th^&12^th^ graders  M & F | Family Connectedness | Multigenerational Interconnectedness Scale | 0.80 | NR | Convergent validity  Construct validity  Cross-cultural adaptation | Average item-total correlation of 0.41  PCA supported an apriori hypothesized 3-factor structure  Three bilingual psychologist made item translations |
| Dwairy (2006) | Saudi Arabia  Yemen  Egypt  Algeria  Jordan Lebanon Palestine  Israel | Cross-sectional | Arab adolescents | 2,893  10^th^&12^th^ graders  M & F | Family connectedness | Multigenerational Interconnectedness Scale | 0.78, 0.77, and 0.75 | NR | Divergent validity  Convergent validity  Construct validity | Correlated negatively with a measure of psychological maturity.  Average item-total correlation of 0.43  PCA analysis yielded seven factors with eigenvalues above 1.00, together accounting for 51.13% of the variance |
| Dwairy (2009) | Israel | Cross-cultural | Immigrant and non-immigrant adolescents | 189  16-17  M&F | Family connectedness | Multigenerational interconnectedness scale | 0.82 for the overall scale  0.70 – 0.81 for the subscales | NR | Construct validity | A PCA, conducted with an a priori 3-factor solution, showed good convergence of the items into three subscales that explained 55.10% of the variance |
| Dwairy (2006) | Egypt | Cross-Sectional | students | 351  16-17  M&F | Family Connectedness | Multigenerational interconnectedness scale | 0.60 – 0.75 | NR | Construct validity | All except one item loaded onto the intended factors on factor analysis with item-factor loadings between 0.18 and 0.71. |
| Dwairy (2010) | France  Saudi Arabia  Poland  Argentina  Kuwait  Israel  India  Jordan | Cross-sectional | Adolescent | 2,884  M&F | Family Connectedness | Multigenerational interconnectedness scale | 0.78 | NR | NR | NR |
| Easterbrook (2013) | UK | Cohort | Students | 112  19.8(3.35)  M & F | Peer connectedness | 1 item | NR | NR | NR | NR |
| Ehman (2020) | USA | Cohort | Adolescents with cancers and comparison peers | 298  8-17 (13.0)  M&F | Peer and family connectedness | HMAC | 0.76 and 0.81 for family and peers, respectively | NR | NR | NR |
| Ehrenreich (2009) | USA | Validation | Clinically anxious adolescents | 65  7-17 (11.4 (2.7))  M&F | Family connectedness | Family Assessment Clinician Interview (FACI) | Interrater reliability (kappa) of 0.79 – 1.00 for the subscales (P < 0.001) | NR | Convergent validity | Correlations (r) between FACI subscales and subscales on the Family environment scale (FES): FACI-protection vs FES-Control (0.29, p = 0.08), FACI-Warmth vs FES-expressiveness (0.38, p < 0.05), FACI-Warmth vs FES-Conflict (-0.33, p < 0.05), FAHI-Warmth vs FES-Cohesion (0.26, p < 0.10), FAHI-Warmth vs FES-Control (0.26, p < 0.10) |
| Eisenberg (2003) | USA | Cross-sectional | Students | 5,882  7^th^ -12^th^ graders  M&F | School Connectedness | 1 item | NR | NR | NR | NR |
| Ellickson (2003) | USA | Cohort | Students | 3,000  18.0  M & F | School connectedness  Community connectedness  Family connectedness  Peer connectedness | 3 items  2 items  6 items  2 items | NR  0.89  0.76  NR | NR  NR  NR  NR | NR  NR  NR  NR | NR  NR  NR  NR |
| Emery (2015) | Canada | Cross-sectional | Students | 302  11.1 (0.9) for elementary school students  15.0 (1.1) for high school students  M&F | Peer, family, and school relatedness | Children’s intrinsic need satisfaction scale – relatedness subscale | -0.75 & 0.67 for overall relatedness subscale across samples  -0.76 – 0.82 across domains for elementary school students  -0.32 – 0.85 across domains for high school students | NR | NR | NR |
| Engles (2020) | Germany | Trial | Students | 285  12.7(1.1)  M&F | School connectedness | 3 items | 0.76 | NR | NR | NR |
| Enns (2020) | Canada | Cross-sectional | Students | 685  7^th^ -12^th^ graders M&F | Family Connectedness | Children Intrinsic Needs satisfaction scale | NR | NR | NR | NR |
| Ernestus (2014) | USA | Cross-Sectional | Students | 260  16.4(1.0)  M&F | School Connectedness | Adolescent Health Survey | 0.70 | NR | NR | NR |
| Espelage (2016) | USA | RCT | Students with disabilities | 123  11 &12 year olds  M & F | School connectedness | PSSM | 0.66 | NR | NR | NR |
| Espelage (2020) | USA | Cohort | Students in relationships or dating | 1668  12.8 (1.1)  M&F | School connectedness | PSSM (4 items) | 0.68 – 0.74 across time points | NR | NR | NR |
| Estrada (2013) | USA | Cross-sectional | Students | 852  7^th^ graders  M & F | School connectedness | School connectedness scale | 0.82 | NR | NR | NR |
| Ethier (2018) | USA | Cohort | Sexual minority students | 497  16.1 (1.6)  M&F | School connectedness | School connectedness scale | 0.76 | NR | NR | NR |
| Evans (2015) | USA | Cross-sectional | Racially/ethnically diverse rural youth | 5752  11-19 (14.42 (1.78))  M & F | Ethnic Identity | MEIM | 0.95 | NR | NR | NR |
| Faircloth (2005) | USA | Cross-sectional | Students | 5494  9^th^-12^th^ grades  M & F | School connectedness | 7 items | 0.71 – 0.76 | NR | NR | NR |
| Faircloth (2011) | USA | Cross-sectional | Students | 598  7^th^ & 8^th^ graders  M&F | School connectedness | Classroom Belonging and Support Scale | 0.84 – 0.90 across time points | NR | Measurement equivalence | Insignificant difference in the fit of the measurement model for this instrument between ethnic groups, grade levels, or gender groups at either time point |
| Farahani (2018) | Australia | Cross-sectional | Migrant adolescents | 327  11-17 (13.7 (1.3))  M&F | School connectedness  Ethnic identity | PSMM – Abridged  MEIM | NR  0.87 | NR | NR  Construct validity | NR  Factor analysis revealed a 1-factor structure which accounted for 60.9% of the variance |
| Farid (2014) | Malaysia | Cross-sectional | Adolescents from welfare institutions | 1082  12-19 (15.7)  M&F | Family connectedness | 2 items | 0.61 | NR | NR | NR |
| Faro (2019) | USA | Cross-sectional | Emerging adults | 249  18-29 (19.7 (2.00)  M & F | Family, school, peer, and community Connectedness | Social Connectedness Scale | 0.91 | NR | NR | NR |
| Faulkner (2009) | Canada | Cross-sectional | Students | 2012  12-20 (14.9 (1.9))  M&F | School connectedness | 3 items | 0.64 | NR | NR | NR |
| Fenaughty (2019) | New Zealand | Cross-sectional | Sexual and gender minority and heterosexual cisgender students | 8500  12-19  M&F | School connectedness | 1 item | NR | NR | NR | NR |
| Fernandez (2019) | USA | Cross-sectional | Hispanic early adolescents | 192  11-15  12.1 (.95) | School connectedness | School connectedness scale | 0.84 | NR | NR | NR |
| Fernández (2020) | Uruguay | Cross-sectional | School going adolescents | 172  12-18 (14.67 (1.62))  M & F | Community connectedness | Personal Wellbeing Index | NR | NR | NR | NR |
| Fernandez-Rio (2014) | Spain | Cross-sectional | Students | 507  12-17 (14.4 (1.7))  M&F | School relatedness | Basic Psychological need in exercise scale | 0.79 | NR | NR | NR |
| Fielder (2013) | USA | Cohort | Students | 483  18-21 (18.1 (0.3))  F | Family connectedness | Parenting style index – involvement subscale | 0.90 | NR | NR | NR |
| Filus (2019) | Greece  Norway  Poland Switzerland | Cross-sectional | Adolescents | 745  18.0  M&F | Family Connectedness | Multigenerational interconnectedness scale | 0.72 – 0.98 | NR | NR | NR |
| Fin (2019) | Brazil | Trial | Students | 61  12-14  M&F | Peer Connectedness | Basic Psychological need in exercise scale | 0.62 – 0.95 | NR | NR | NR |
| Fitzpatrick (2008) | USA | Cross-sectional | Low-income African American adolescents | 1526  5^th^-12^th^ graders  M&F | School connectedness  Religious connectedness | 1 item  3 items | NR  NR | NR  NR | NR  NR | NR  NR |
| Fleming (2007) | New Zealand | Cross-sectional | Students | 9,570  9-13  M&F | Family connectedness  School connectedness | 5 items  4 items | NR  NR | NR  NR | NR  NR | NR  NR |
| Fonesca (2002) | USA | Cross-sectional | Students | 9,042  12-18  M&F | Family Connectedness | Voice of Connecticut youth survey | 0.72 | NR | NR | NR |
| Forber-Pratt (2020) | USA | Cross-sectional | Students | 11,353  14-18 (15.9 (1.2))  M&F | School Connectedness | 6 items from an unpublished measure | 0.86 | NR | NR | NR |
| Forrest (2013) | USA | Cross-sectional | Students | 1,479  9-13  M&F | School Connectedness | Healthy pathway child report | NR | NR | NR | NR |
| Fosco (2012) | USA | Cross-sectional | Students | 179  6^th^ graders  M&F | Family connectedness | Connectedness with caregivers subsection of the teen supplemental survey | 0.90 for mothers, 0.87 for fathers | NR | NR | NR |
| Fosco (2020) | USA | Cross-sectional | Students | 151  13-16 (14.6 (0.8))  M&F | Family connectedness | 4 items | 0.95 and 0.89 (intra- and inter-rater reliability) | NR | NR | NR |
| Foster (2017) | USA | Cross-sectional | Students | 224  12-15 (13.9 (1.1))  M&F | School connectedness  Peer connectedness  Family connectedness  Community Connectedness | School connectedness scale  HMAC  Parent-family connectedness scale  Community connectedness scale | 0.84  0.79  0.90  0.75 | NR  NR  NR  NR | NR  NR  NR  NR | NR  NR  NR  NR |
| Fousiani (2016) | Cyprus | Cross-sectional | Students | 548  10^th^-11^th^ graders  M&F | Family, school, peer, and Community relatedness | Basic Psychological Needs satisfaction | 0.80 | NR | NR | NR |
| Frank-waldheter (2015) | USA | Cross-sectional | Low social economic Adolescents | 28  15.5 (1.1)  M&F | Family Connectedness | Relationship problem inventory | NR | NR | NR | NR |
| Fraser (2009) | Australia | Cross-sectional | Children of parents with mental illness | 44  12-17 (13.0 (1.6))  M&F | Peer, family, school, and community connectedness | Social connectedness scale | NR | NR | NR | NR |
| Freeze (2014) | South Africa | Cross-sectional | Adolescent boys with and without conduct disorder | 80  13-18  M | Family connectedness | The Parental Bonding Instrument | NR | NR | NR | NR |
| French (1995) | USA | Cross-sectional | Students | 33,393  15.0 (1.8)  M&F | Family connectedness  School connectedness  Community connectedness | 1 item  1 item  1 item | NR  NR  NR | NR  NR  NR | NR  NR  NR | NR  NR  NR |
| Fritsch (1990) | USA | Cross-sectional | Inpatient adolescents | 84  12.5-18.5 (15.6)  M&F | Peer relatedness | Engagement sub-scale of the Chestnut Lodge Adolescent Interaction scale | NR | NR | NR | NR |
| Froiland (2016) | USA | Cross-sectional | Students | 110  9^th^ graders  M&F | School connectedness | 5 items created by the National center for Education Statistics | NR | NR | NR | NR |
| Frostick (2018) | UK | Cross-sectional | Adolescents from areas of deprivation | 639  11-16 years  M&F | School connectedness | 5 items taken from the school connectedness scale | NR | NR | NR | NR |
| Frydenberg (2009) | Australia | Cohort | Metropolitan Catholic schools | 536  12&14  M & F | School connectedness | Items adapted from What is Happening in This Class questionnaire, the Beyond blue student survey, and the Drug Education Evaluation and Monitoring (DEEM) Project survey | 0.86 | NR | NR | NR |
| Fulkerson (2007) | USA | Cross-sectional | At-risk-for-overweight and overweight youths | 1351  7^th^-12^th^ graders  M&F | Family connectedness | New 4-item Family connectedness scale | 0.69 | NR | NR | NR |
| Furlong (2011) | USA | Validation | Students | 500,800  11-18  M&F | School Connectedness | School connectedness scale | 0.82 – 0.88 across groups | NR | Construct validity  Concurrent validity  Measurement invariance | CFA confirmed a one-factor structure across sociocultural groups  Correlations of 0.44 to 0.55 across sociocultural groups between the scale and a social support scale  Configural (χ2 (572.24), df = 90, p < 0.001, RMSEA = 0.073, CFI = 0.980, NNFI = 0.961), metric (χ2 (718.71), df = 158, p < 0.001, RMSEA = 0.060, CFI = 0.977, NNFI = 0.974), and scalar (χ2 (1310.29), df = 226, p < 0.001, RMSEA = 0.063, CFI = 0.977, NNFI = 0.964) equivalence confirmed across sociocultural groups |
| Gaete (2016) | Chile | Validation | Early adolescents | 1250  12-15  (11.9 (1.2))  M & F | School connectedness | PSSM | 0.92 | NR | Construct validity | Factor analysis yielded a 1-factor structure with excellent fit to the data after removing the negatively worded items: GFI = 0.99; AGFI = 0.99; NFI = 0.99; RFI = 0.99; RMSR = 0.04 |
| Gagne (2014) | Canada | Cross-sectional | Students | 211  10-18 (14.8 (1.5))  M&F | School connectedness | PSSM | 0.89 | NR | NR | NR |
| Gaias (2019) | USA | Cross-sectional | Adolescents | 1857  6^th^-11^th^ graders  M & F | School connectedness | The Spanish version of the US-based Maryland Safe and Supportive Schools School Climate Survey | 0.84 | NR | NR | NR |
| Gaias (2020) | USA | Cross-sectional | Students | 133  9^th^ graders  M&F | School Connectedness | PSSM | 0.82 and 0.88 across time points | NR | NR | NR |
| Galliher (2004) | USA | Cohort | Students | 7,613  16.3  M&F | School Connectedness | School connectedness Scale | 0.77 | NR | NR | NR |
| Galliher (2011) | USA | Cohort | Students | 137  14-19 (15.2 (1.0))  M&F | Ethnic identity | MEIM | 0.63 – 0.92 | NR | NR | NR |
| Gao (2015) | China | Cross-sectional | Students | 677  12-17 (14.4)  M&F | School connectedness | PSSM | 0.83 | NR | NR | NR |
| Gao (2019) | China | Cross-sectional | Internet addicted high school students | 2271  15.99 (0.92)  M & F | School connectedness | Child Rating Scale (CRS) | 0.76 | NR | NR | NR |
| Gao (2020) | China | Cross-sectional | Students | 539  17-22 (19.9 (1.0))  M&F | School connectedness | The dormitory belonging scale | 0.81 | NR | Construct validity | CFA yielded acceptable goodness-of-fit statistics: χ2/df = 2.56, RMSEA = 0.05, GFI = 0.96, CFI = 0.98, IFI = 0.98 |
| Gao (2020) | China | Cross-sectional | Students | 1,201  19.4 (1.4)  M & F | School Connectedness | School Connectedness scale | 0.76 | NR | NR | NR |
| Garcia-Moya (2020) | UK and Spain | Development and validation | Students | 10,323  11-15  M&F | School Connectedness | New Student-Teacher Connectedness Scale | Composite reliability of 0.98 for the overall scale in the UK, 0.91 – 0.97 for subscales  Composite reliability of 0.98 for the overall scale in Spain, 0.88 – 0.96 for subscales | NR | Construct validity  Content validity  Convergent validity  Measurement invariance | CFA supported a 3-factor model with good model fit indices in both countries  Item development was guided by the definition of student–teacher connectedness and its dimensions presented in the literature  In the UK, the scale correlated positively with a general measure of teacher support (r = 0.50), school satisfaction (r = 0.35), and life satisfaction (r = 0.17) and negatively with school-related stress (r = 0.13). In Spain, Connectedness scores correlated positively with teacher support (r = 0.59), school satisfaction (r = .31), and life satisfaction (r = 0.28) and negatively with school-related stress (r = 0.15)  Configural (ꭓ2 = 2572.821, df = 102, CFI = 0.993; TLI = 0.991, SRMR = 0.014; RMSEA = 0.075), metric (ꭓ2 = 2529.494, *df = 111*, CFI = 0.993, ΔCFI = 0.000; ΔSRMR = 0.14; ΔRMSEA = 0.004, TLI = 0.992, RMSEA = 0.071), and scalar invariance (ꭓ2 = 2796.400, *df = 132*, CFI = 0.992, ΔCFI = 0.001; ΔSRMR = 0.17; ΔRMSEA = 0.003, TLI = 0.992, RMSEA = 0.068) established across countries |
| Gazis (2010) | Australia | Cross-sectional | Indigenous students | 274  13.6 (0.7)  M&F | Ethnic identity | MEIM | 0.85 | NR | Cross-cultural adaptation | Scale modified for local use by first replacing references of ethnicity with culture. Scale then modified to reflect the local idioms |
| Géczy (2020) | Canada | Cross-sectional | Adolescents | 29,315  7th-12^th^ graders  M & F | Family connectedness  School connectedness | 11 items  6 items | NR  NR | NR  NR | NR  NR | NR  NR |
| Geng (2012) | China | Cross-sectional | Migrant adolescents | 36  11-14 (12.5 (1.0))  M&F | Community connectedness | Sense of Belonging Questionnaire (sense of belonging to the countryside or the cities subscales) and the Brief Implicit Association Test | 0.88 for the overall scale  0.85 and 0.87 for the sub-scales | NR | NR | NR |
| Geng (2020) | China | Cohort | Students | 77,150  14.9 (1.8)  M&F | School relatedness  Peer relatedness | New 3-item Teacher-Students Relationship Scale  New 3-item peer relationships scale | 0.92, 0.86, and 0.84 across grades  0.78, 0.77, and 0.82 across grades | NR  NR | Construct validity  Construct validity | 1-factor structure with factor loadings above 0.74; CFI = 1.00, RMSEA = 0.00, SRMR = 0.00, χ2 (df) = 208.067 (3), P < 0.05  1-factor structure with factor loadings above 0.70; CFI = 1.00, RMSEA = 0.00, SRMR = 0.00, χ2 (df) = 145.532 (3), P < 0.05 |
| Georgiades (2013) | USA | Cohort | Students | 77150  14.9 (1.8)  M&F | School Connectedness | School connectedness scale | 0.76 – 0.82 | NR | NR | NR |
| Gerard (2015) | USA | Cross-sectional | Students | 675  8^th^-11^th^ graders  M&F | School Connectedness | Marjoribanks school capital scale | 0.63 | NR | NR | NR |
| Gerra (2020) | 28 countries^2^ | Cross-sectional | Students | 50,436  16-year olds  M&F | School connectedness | 1 item | NR | NR | NR | NR |
| Gervais (2020) | New Zealand | Cohort | General adolescent population | 1774  10-17 years  M&F | Family connectedness | 11 items from FACES-II | 0.90 at TI, 0.91 at T2, 0.93 at T3 | NR | NR | NR |
| Gharaei (2018) | Belgium | Cross-sectional | Moroccan and Turkish minority youth | 1489  12-18  M & F | Community connectedness | 1 item | NR | NR | NR | NR |
| Ghobadzadeh (2016) | USA | Cross-sectional | Sexually active adolescent girls at elevated risk for pregnancy | 253  13-17 years  F | School connectedness  Peer  Connectedness  Family connectedness | School connectedness scale  6 item scale adapted from the Add Health  5 item scale adapted from the parent-family connectedness scale | 0.87  0.89  0.86 | NR  NR  NR | NR  NR  NR | NR  NR  NR |
| Gillet (2009) | USA | Cohort | Adolescents with eating disorder | 102  19.3 (2.4)  F | Family Connectedness | Family implicit rules profile (FIRP) | 0.82 – 0.92 | 0.94 | Construct validity | Stable factors were identiﬁed on PCA that corresponded to the subscales with loadings ranging from 0.41 to 0.89. |
| Gillson (2008) | UK | Cross-sectional | Students | 63  11-12 (11.5)  M&F | School relatedness | Need satisfaction in the workplace questionnaire | 0.73 | NR | NR | NR |
| Gilman (2006) | USA | Cross-sectional | Students | 654  14.8 (1.6)  M&F | School Connectedness | PSSM | 0.90 | NR | NR | NR |
| Girelli (2019) | Italy | Validation | General adolescent population | 308  14.0  M&F | Peer relatedness | 12 items from the Basic Psychological Needs Satisfaction scale | 0.80 for the relatedness subscale | NR | Criterion validity  Content validity  Construct validity  Measurement invariance  Cross-cultural adaptation | Statistically significant positive correlation of each of the three psychological needs with psychological well-being (0.43, p < 0.001 for the relatedness sub-scale)  Items that were highly rated for clarity by adolescents and experts were adapted for this measure  CFA confirmed a 3-factor structure with good model fit: CFI = 0.962, RMSEA = 0.059, SRMR = 0.037  Multi-group CFAs showed configural, metric and scalar invariance of the scale across gender (Metric invariance: ∆CFI = 0.006; Scalar invariance: ∆CFI = 0.003)  Scale translated to Italian and back-translated to English using standardized procedures |
| Gnambs (2016) | Austria | Cohort | Adolescents | 600  11-16  M & F | School relatedness | Basic psychological needs scale | 0.68 | NR | NR | NR |
| Godor (2017) | Belgium  Czech Republic Estonia Finland  France Germany Italy Netherlands  Poland Slovak Republic Slovenia Spain  UK | Cross-sectional | Students | 81506  15& 16 years  M & F | School connectedness | Sense of belonging scale | 0.82 | NR | NR | NR |
| Golaszewski (2018) | USA | Cross-sectional | Students | 639  11-25 (12.2 (1.0))  M&F | School connectedness | School connectedness scale | 0.82 | NR | NR | NR |
| Goldbach (2018) | USA | Cross-sectional | Students | 3221  6^th^ 12^th^ graders  M & F | School connectedness | 4 items | 0.68 | NR | NR | NR |
| Goldfarb (2017) | USA | Cohort | Students | 14,916  12-16 (14.7 (1.4))  M&F | Family connectedness | Youth relationship with parent index from the National Longitudinal survey of youth | 0.71 – 0.85 | NR | NR | NR |
| Goldweber (2013) | USA | Cross-sectional | Secondary school students | 12,763  6^th^-12^th^ graders  M & F | School connectedness | 2 items | NR | NR | NR | NR |
| Gonzalez (2014) | USA | Cross-sectional | Latino youth | 179  14.1 (1.33)  M & F | School connectedness | PSSM | 0.83 | NR | NR | NR |
| Goodenow (1993) | USA | Development and validation | Students | 1365  9-16 years  M&F | School connectedness | PSSM | -0.875 and 0.884 for urban students at 2 time points  -0.803 and 0.771 for urban students for English and Spanish versions, respectively |  | Construct validity | -Suburban students reported stronger connectedness than urban students as predicted (3.86 vs. 3.10, t [7531] = 20.0, p< 0.001)  -Newcomers to the town scored significantly lower than the residents as predicted (F= 7.16, p< 0.01)  -Significant differences in belonging scores as predicted with respect to minority status, special education status, and gender (F= 5.41, p < 0.001)  -Students rated as having high, medium, or low social standing were different in their PSSM scores as predicted F(2,451] = 26.59, < 0.001) |
| Goodyear (2002) | USA | Cross-sectional | Pregnant adolescents | 493  16.8 (1.3)  F | Ethnic identity | MEIM – Ethnic belonging subscale | 0.82 | NR | NR | NR |
| Gordon (2020) | USA | Cohort | Adolescents | 12,939  7^th^ -12^th^ graders  M & F | Community connectedness | 6 items | 0.52 | NR | NR | NR |
| Gordon (2013) | USA | Cohort | Students | 578  6^th^-10^th^ graders  M&F | Self, peer,  Family, and school connectedness | HMAC | NR | NR | NR | NR |
| Gore (2016) | USA | Cross-sectional | Students | 187  11-18  M & F | School Connectedness | School Connectedness Scale | 0.81 | NR | NR | NR |
| Gottlieb (2016) | USA | RCT | Youths depression | 439  12-17  M & F | Family connectedness | Family Assessment Measure–3rd edition (FAM-III) | NR | NR | NR | NR |
| Govender (2013) | South Africa | Cross-sectional | Students | 241  13-17 (14.7 (0.74))  M&F | School Connectedness | PSSM | 0.84 | NR | NR | NR |
| Gower (2018) | USA | Cross-sectional | Transgender and Gender Diverse Youth | 2168  5^th^-11^th^ graders  M & F | Family connectedness | 3 items | 0.75 | NR | NR | NR |
| Gowing (2019) | Australia | Cross-sectional | Students | 336  13-18 (15.1 (1.7))  M&F | School Connectedness | The School Connectedness Scale | NR | NR | NR | NR |
| Goyer (2019) | USA | Trial | Negatively stereotyped adolescents & comparison peers | 806  6^th^&7^th^ graders  M&F | School connectedness | 10 item- social and academic fit scale | 0.82 | NR | NR | NR |
| Graupensperger (2020) | USA | Cohort | Athletes | 135  19.8 (1.4)  M&F | Peer connectedness | 5 items | 0.81 | NR | NR | NR |
| Gray (2012) | Cambodia | Case control | Survivors of sexual violence | 47  15.6 (2.7)  F | Family, school, peer, and community relatedness | The Resilience Scales for children and Adolescents | NR | NR | NR | NR |
| Gregory (2019) | Australia | Validation | School Children | 51,574  10-13 (12.06 (0.60))  M&F | School  Family and Peer connectedness  School connectedness | Middle Years Development Instrument (MDI) – Connectedness Subscale  EPOCH Measure of Adolescent Wellbeing | 0.78 – 0.95 across age and gender  NR | 0.40 – 0.66  NR | Construct validity  Convergent and discriminant validity  NR | 5-factor model of the connectedness subscale was supported on CFA: CFI = 0.99, TLI = 0.99, RMSEA = 0.05  Correlation within scales in the connectedness domain (r = 0.25 – 0.51), and expected correlations between the connectedness subscale and EPOCH measure, including subscales (0.25 – 0.56)  NR |
| Gregory (2020) | UK | Cross-sectional | Autism siblings and comparison peers | 122  11-16  M & F | School connectedness | The belonging scale adapted from PSSM | 0.826 | NR | NR | NR |
| Guay (2008) | Canada | Cohort | Students | 834  17.7 (1.9)  M&F | Peer and family relatedness | Relatedness scale | 0.96 for each construct | NR | NR | NR |
| Guay (2017) | Canada | Cohort | Students from disadvantaged backgrounds | 946  7^th^-10^th^ graders  M&F | Peer, family, and school relatedness | Interpersonal relationship quality scale | McDonald’s ω ranged between 0.79 and 0.80 for all constructs | NR | NR | NR |
| Gummadam (2016) | USA | Cross-sectional | Students | 311  19.3 (2.3)  M&F | School Connectedness | PSSM | 0.89 | NR | Construct validity | Subscales were positively correlated with one another, as would be expected given that they are subscales of the same measure. |
| Gungor (2017) | Belgium | Cross-sectional | Immigrant youth | 290  19.9 (1.3) for natives  19.5 (1.9) for immigrants  M&F | Peer and family relatedness | Resilience Scale – relatedness sub-scale | 0.81 | NR | Construct validity  Cross-cultural equivalence  Convergent validity | 4-factor structure accounting for 33% and 37% of the variance for natives and immigrants, respectively.  Good model fit statistics: χ2 (88) = 180.51, p < 0.001, χ2/df = 2.05, RMSEA = 0.06, SRMR = 0.07  Significant correlations of the subscale with relationship satisfaction (B = 0.28 and 0.22, p < 0.01 and p < 0.05 for both groups) |
| Haapala (2017) | Finland | Cross-sectional | Students | 758  14.5 (.04)  M&F | School relatedness | Need for relatedness scale | 0.90 | NR | NR | NR |
| Haapala (2014) | Finland | Cross-sectional | Students | 1463  12.9  M&F | Peer Relatedness  School relatedness | Need for relatedness scale  School wellbeing profile | NR | NR | NR | NR |
| Haerens (2019) | Belgium | Cross-sectional | Students | 659  14.7  M&F | School relatedness | Basic Psychological need Satisfaction and Frustration Scale (BPNSFS) | NR | NR | NR | NR |
| Hagborg (1998) | USA | Case control | Adolescent with Learning Difficulties | 74  9^th^-12^th^ graders  M&F | School Connectedness | PSSM | NR | NR | NR | NR |
| Hale (2005) | USA | Cross-sectional | College students | 247  17- 4 (19.36 (1.33))  M & F | Peer connectedness | Interpersonal Support Evaluation List, College Version (ISEL2) | 0.65 | NR | Convergent validity | Correlation of 0.46 between the scale and the Inventory of Socially Supportive Behavior |
| Hall (2007) | USA | Cross-sectional | Adolescents from the general population | 4,746  11-18  M & F | School connectedness  Family connectedness | 1 item  4 items | NR  NR | NR  NR | NR  NR | NR  NR |
| Hamm (2005) | USA | Cross-sectional | Students | 285  6^th^ graders  M&F | School connectedness | Classroom belonging and social support scale | 0.68 at T1 and 0.72 at T2 | NR | NR | NR |
| Handebo (2018) | Ethiopia | Cross-sectional | Students | 635  17.6 (1.5)  M&F | Family connectedness  Religious connectedness  School connectedness  Peer connectedness | 9 items  9 items  7 items  7 items | NR  NR  NR  NR | NR  NR  NR  NR | NR  NR  NR  NR | NR  NR  NR  NR |
| Harding (2015) | UK | Cohort | Students | 6,643  11-16  M&F | Family Connectedness | Family Affluence scale | NR | NR | NR | NR |
| Hardway (2006) | USA | Cross-sectional | Adolescents with immigrant backgrounds | 489  14.9 (0.4)  M&F | Family connectedness | FACES-II and 3 items adapted from a previous study | 0.85 – 0.88 across groups for FACES-II  0.63 – 0.75 across groups for the 3 items | NR | NR | NR |
| Harris (2019) | USA | Cross-sectional | African American adolescent males | 96  16-21 (18.0)  M | Family connectedness | Father-Son Closeness and Connectedness Scale | 0.91 | NR | Construct validity | EFA revealed a 2-factor structure with items highly loading in their intended factors |
| Hatchel (2019) | USA | Cross-sectional | Ethnically diverse sample of transgender adolescents | 4778  10-18 (14.71)  M & F | School connectedness | 5 items | 0.85 | NR | NR | NR |
| Hatchel (2019) | USA | Cross-sectional | Sexual minority adolescents | 934  14-18 (15.9 (1.2))  M&F | School Connectedness | 6 items | 0.80 | NR | NR | NR |
| Hazzard (2020) | USA | Cohort | Students | 13,532  15.4 (0.1)  M&F | Family connectedness | Youth asset survey – relationship with mother and relationship with father subscales | 0.86 and 0.90 for both sub-scales, respectively | NR | NR | NR |
| He (2015) | USA | Cross-sectional | Adolescents involved with child welfare | 995  11-17 years  M&F | Peer connectedness  Family relatedness | The loneliness and social dissatisfaction questionnaire  Relatedness scale of the Rochester Assessment Package for Schools | NR  0.85 | NR  NR | NR  NR | NR  NR |
| He (2020) | Germany  Italy  Spain | Cross-sectional | Immigrant and non-immigrant students | 23,500  15-year olds  M&F | School connectedness | Sense of belonging at school measure | 0.85 in Germany  0.81 in Italy 0.88 in Spain | NR | Measurement invariance | Scalar invariance was supported within each country whereas collapsing the  countries for a six-group comparison lacked scalar invariance |
| Hebron (2018) | UK | Cohort | Adolescents with autism spectrum disorder | 54  10-13  M&F | School Connectedness | PSSM | 0.89 | NR | NR | NR |
| Heikamp (2020) | Belgium | Cross-sectional | Immigrant adolescents | 1,050  15.1(1.2)  M&F | School Connectedness | PSSM | 0.84 – 0.87 | NR | NR | NR |
| Henrich (2001) | USA | Validation | Students | 499  6^th^ &7^th^ graders  M & F | Peer relatedness | Neediness and Relatedness subscales of the Depressive Experiences Questionnaire for Adolescents (DEQ–A) Interpersonal Concerns factor | 0.67 | NR | Convergent validity | Bivariate correlation between neediness and relatedness was substantial (r = 0.70, p < 0.01). Efficacy was positively associated with Relatedness (r = 0.23, p < 0.01 |
| Henrich (2005) | USA | Cohort | Adolescents | 7033  16.0  M & F | School connectedness  Family Connectedness | School connectedness scale  Parent-family connectedness scale | 0.76  0.88 | NR  NR | NR  NR | NR  NR |
| Hernandez (2017) | USA | Cohort | Mexican-origin youth | 674  10.4  M&F | School connectedness  Ethnic identity | 7 items from the School Attachment Scale  4 items from MEIM and 2 items from a previous study | 0.60 – 0.79  0.75 – 0.88 | NR  NR | Construct validity  Cross-cultural adaptation  NR | Unidimensional structure with good model fit: CFI = 0.94, RMSEA < 0.06) in 5^th^ grade, CFI = 0. 96, RMSEA < 0.06) in 7^th^ grade, CFI = 0.97, RMSEA < 0.08 in 9^th^ grade  Scale translated to Spanish and back-translated to English by independent translators  NR |
| Herrenkohl (2009) | USA | Cohort | Adolescents | 754  10-27  M&F | School Connectedness | 7 items | 0.73 | NR | NR | NR |
| Hignett (2018) | UK | Cohort | At risk adolescents | 58  12-16  M&F | Family, Peers, and Community Connectedness | Inclusion of others in Self | NR | NR | NR | NR |
| Hilario (2014) | Canada | Cross-sectional | Asian Immigrants | 1,225  12-19  M&F | School connectedness  Family connectedness  Cultural connectedness | 6 items from the British Columbia Adolescent Health Survey  11 items from the British Columbia Adolescent Health Survey  6-item cultural connectedness measure adapted from the MEIM | NR  NR  NR | NR  NR  NR | NR  NR  NR | NR  NR  NR |
| Hill (2019) | USA | Cross-sectional | Adolescents in psychiatric hospital | 387  12-17 (14.7 (1.5))  M&F | School, family, peer, and community connectedness | The interpersonal Needs Questionnaire (INQ) | 0.93 | NR | NR | NR |
| Hirota (2020) | Japan | Cross-sectional | Students | 7759  9-15 years  M&F | School connectedness | Social capital questionnaire for adolescent students- school social cohesion and network subscale | 0.81 | NR | NR | NR |
| Ho (2017) | Australia | Cross-sectional | Recently arrived Chinese adolescent migrants | 55  11-17 (14.26 (1.47))  M & F | School connectedness | PSSM | NR | NR | NR | NR |
| Ho (2015) | China | Cross-sectional | Students | 775  12-14  M&F | School connectedness  Family Connectedness | School connectedness scale  Family connectedness scale | NR  NR | NR  NR | NR  NR | NR  NR |
| Hoferichter (2015) | Canada and Germany | Cross-sectional | Students | 1,088  12-15 (13.7 (0.5))  M&F | School connectedness | School belonging scale from the Programme for International Student Assessment (PISA) | 0.72 & 0.83 in Canada and  0.70 & 0.78 in Germany for classroom belonging and teacher belonging sub-scales, respectively | NR | NR | NR |
| Hoffman (2017) | USA | Cross-sectional | Students | 3296  13.0 (1.0)  M&F | School connectedness | Community and youth collaborative institute school experience scales | NR | NR | NR | NR |
| Hold (2008) | USA | RCT | At-risk, urban minority adolescents | 40  9^th^ graders  M & F | School connectedness | PSSM | 0.73 | NR | NR | NR |
| Hollifield (2015) | USA | Cohort | European American adolescents | 337  17.7  M&F | Family relatedness | Network of relationships inventory | 0.90 | NR | NR | NR |
| Holligan (2020) | Canada | Cohort | Binge drinking Students | 5,238  9^th^-12th graders  M&F | School Connectedness | 6 items | 0.83 | NR | NR | NR |
| Hong (2020) | USA | Cohort | Adolescents | 4573  13.4  M&F | School Connectedness | HBSC | NR | NR | NR | NR |
| Horwitz (2021) | USA | Cross-sectional | Sexual minority youth seeking emergency medical services | 6423  12-17 years  M&F | School connectedness  Family connectedness  Peer connectedness | School connectedness scale  Parent-family connectedness scale  HMAC | 0.79  0.75  0.78 | NR  NR  NR | NR  NR  NR | NR  NR  NR |
| Houltberg (2011) | USA | Cross-sectional | Students | 248  14.8 (14-16)  M&F | Family connectedness | FACES-II and parental behavior measure | 0.83 for FACES-II  0.80 for mothers and 0.86 for fathers in the parental behavior measure | NR | NR | NR |
| Howard (2015) | USA | Cohort | Adolescents with cancer & comparison peers | 254  8-19 years  M&F | Peer, family, school, and community connectedness | HMAC | 0.73 – 0.89 across the sub-scales | NR | NR | NR |
| Howle (2016) | Australia | Cohort | Students | 399  13.43 (1.21)  F | School relatedness | Subscale of the Need for Relatedness Scale | 0.95 | NR | NR | NR |
| Hsiao (2008) | USA | Case control | Adolescents | 313  14.2  M&F | Ethnic identity | MEIM | 0.83 | NR | NR | NR |
| Hsieh (2019) | Taiwan | Cross-sectional | Students | 6445  7^th^-12^th^ graders  M&F | School connectedness | 5 items | 0.68 | NR | NR | NR |
| Huang (2020) | USA | Cross-sectional | Students | 5712  15.8 (0.3)  M&F | School connectedness | 6 items | 0.84 | NR | NR | NR |
| Huey (2020) | USA | Cohort | Students | 917  14-17 (15.64)  M & F | Family connectedness | Positive Youth Development Measure | 0.86 – 0.89 | NR | NR | NR |
| Hughes (2015) | USA | Cohort | Academically at-risk Latino students | 204  1^st^ -9^th^ graders  M & F | School connectedness | PSSM | 0.89 |  | Measurement invariance | A comparison of the factor structure of the Year 4 and Year 8 questionnaires demonstrated that longitudinal measurement invariance was achieved |
| Hui (2011) | China | Cross-sectional | Students | 461  12-20 (15.6 (1.8))  M&F | Family relatedness | Parent-Child Relationship Quality Scale | 0.90 and 0.91 for mother and father sub-scales, respectively | NR | NR | NR |
| Hunt (2002) | USA | Cross-sectional | Students | 304  1^st^ -12^th^ graders  M& F | School connectedness | 5 items | 0.89 | NR | NR | NR |
| Hurd (2018) | USA | Cross-sectional | Students | 28,104  15.9 (1.3)  12-21 years  M&F | School connectedness | 6 items | 0.80 | NR | NR | NR |
| Hussain (2018) | USA | Validation | American Indian and Native Alaska students | 249  11-16 (12.9 (1.2))  M&F | School connectedness | PSSM | 0.81 for each group | NR | Construct validity  Measurement invariance | EFA revealed a 2-factor structure with a better fit than the original 3-factor structure: χ2 (78) = 1699.62, p < 0.001  Correlations between the factor loadings for the two groups and similar random correlations provided evidence of measurement invariance across the two groups |
| Huynh (2016) | USA | Cross-sectional | Immigrants | 350  17.2 (0.8)  M&F, | School Connectedness | 12 items adapted from Tyler’s work on Institutional engagement | NR | NR | NR | NR |
| In (2019) | USA | Cross-sectional | Students | 873  4^th^-6^th^ graders  M&F | School Connectedness | The Community and Youth Collaborative Institute-School Experiences Survey (CAYCI-SES) | 0.74 | NR | NR | NR |
| Ingram (2019) | USA | Cohort | Students | 118  11-14 (12.5 (0.6))  M&F | School Connectedness | PSSM | 0.60 | NR | NR | NR |
| Inguglia (2018) | Italy | Cross-sectional | Adolescents attending four secondary school | 556  16-19 (17.25, (0.92).  M & F | Family, school, peer, and community relatedness | Basic Psychological Need Satisfaction and Frustration Scale (BPNSFS) | 0.79 - 0.81 | NR | NR | NR |
| Inguglia (2011) | Italy | Cross-sectional | adolescents | 331  16-19 (17.4 (1.1))  F&M | Family Relatedness | Relationship-with-mother-father questionnaire | 0.70 – 0.82 | NR | NR | NR |
| Inguglia (2015) | Italy | Cross-sectional | Adolescents | 325  17-18 (17.9 (0.7))  M&F | Family, school, peer, and community relatedness | Basic psychological needs scale (BPNS) | 0.84 | NR | NR | NR |
| Isakson (1999) | USA | Cohort | Students | 41  13.7  M&F | School Connectedness | PSSM | 0.91 | NR | NR | NR |
| Ja (2017) | New Zealand | Cohort | Students | 1996  10-15 (12.21 (1.75))  M & F | School connectedness  Peer connectedness  Family connectedness | 6 items adapted from PSSM and School connectedness scale  New 7-item Peer connectedness scale  New 11-item Family connectedness scale | 0.85 – 0.88  0.69 – 0.73  0.90 – 0.92 | NR  NR  NR | Predictive validity | Connectedness scales predicted lostness and confidence over time |
| Jain (2018) | USA | Cross-sectional | Students dating | 112,378  14-17  M&F | School Connectedness | 4 items | 0.80 | NR | NR | NR |
| Jaremka (2017) | USA | Cross-sectional | Adolescents | 173  18.85 (1.16)  M&F | Family, school, peer, and community connectedness | State Social Disconnection Scale | 0.91 | NR | NR | NR |
| Jetten (2015) | Australia | Cross sectional | Adolescents | 827  12-16  M&F | Peer, family, school, and community Connectedness | Exeter Identity Transition Scale | NR | NR | NR | NR |
| Jõesaar (2011) | Estonia | Cross-sectional | Young athletes | 659  6-17 (12.7 (1.7))  M&F | Peer relatedness | Basic Psychological Needs in Exercise Scale | 0.87 | NR | NR | NR |
| Jones (2013) | USA | Development and validation | Adolescents in out-of-home care | 53  15-20 (17.2)  M&F | Peer connectedness | Youth Connections Scale | NR | 0.88 for the overall scale  0.77-0.86 for subscale | Concurrent validity  Face validity | Significant strong correlation (r=0.74, p=0.001) between the scale and the Social Support Behaviors scale  Experts in the field of child welfare reviewed the scale for face validity and determined it to have strong face validity |
| Jose (2012) | New Zealand | Cohort | Nationally representative sample of adolescents | 1774  10-15  M&F | School connectedness  Community connectedness.  Peer connectedness  Family connectedness | 6 items adapted from PSSM and school connectedness scale  4 items adapted from the Sense of Community Index  7 items  New 11-item family connectedness scale constructed using items generated for the study and from FACES-II instrument | 0.87  0.74  0.89  0.91 | NR  NR  NR  NR | Convergent validity  NR  NR  NR | High correlations between connectedness and wellbeing (0.79–0.88)  NR  NR  NR |
| Joyce (2015) | USA | Cohort | Sexual minority youth | 18,924  11-21 years  80.0% < 18 years  M&F | School connectedness | 3 items | 0.77 | NR | NR | NR |
| Juvonen (2019) | USA | Cohort | Students | 4,086  7^th^-8^th^ graders  M&F | School Connectedness | Gottfredson’s Effective School Battery | 0.83 | NR | NR | NR |
| Kakihara (2010) | Sweden | Cohort | Students | 1,022  12-17 (14.3 (1.0))  M&F | Family Connectedness | 5 items | NR | NR | NR | NR |
| Kaminski (2010) | USA | Cross-sectional | Students | 4,131  12-18 (15.1)  M&F | Peer, family, and school connectedness | Vaux Social Support Record | 0.72 for the overall scale  0.85 – 0.90 for the subscales | NR | NR | NR |
| Karcher (2003) | USA | Cross-sectional | Students | 177  Grade 8-12  M & F | Self, family, school & peer connectedness | HMAC | 0.71-0.89 | NR | NR | NR |
| Karcher (2004) | USA | Cross-sectional | Students | 91  6^th^-12^th^ graders  M&F | Ethnic identity | MEIM | 0.83 and 0.77 | NR | NR | NR |
| Karcher (2005) | USA | Cross-sectional | Students | 303  6^th^-10^th^ graders  M&F | Peer, family, and school connectedness | HMAC | NR | NR | NR | NR |
| Karcher (2005) | USA | Trial | Rural students | 73  4&5^th^ graders  M&F | School, family, and peer connectedness | HMAC | 0.85, 0.82 and 0.73 for the subscales, respectively | NR | NR | NR |
| Karcher (2008) | USA | Trial | Students | 516  13.1 (2.28)  M & F | Peer and School connectedness | HMAC | NR | NR | NR | NR |
| Karcher (2010) | USA | Validation | Students | 3,328  6^th^-8^th^ graders  M&F | Self, peer, family, school, and community connectedness | HMAC | 0.76 – 0.85 across sub-scales | NR | Construct validity  Measurement invariance | Overall good model fit: χ2 (1439) =  12,555.58, p < 0.001, CFI = 0.964, RMSEA = 0.051, SRMR = 0.048  Good model fit for both genders and adequate-to-good model fit for ethnic groups demonstrated factorial invariance; Configural invariance provided good model fit across gender (χ2 = 14,327.13, df = 2878, p < 0.001, CFI = 0.963, RMSEA = 0.051, SRMR = 0.051) and ethnic groups (χ2 = 15,380.00, df = 4317, p < 0.001, CFI = 0.961, RMSEA = 0.052, SRMR = 0.062) |
| Karreman (2012) | Netherlands | Cross-sectional | Students | 131  15-19  M & F | Peer connectedness | 30-item Autonomy–Connectedness Scale | 0.76 - 0.80 | NR | NR | NR |
| Karwautz (2003) | Austria | Cross-sectional | Female adolescents with anorexia nervosa and comparison peers | 31  15.7 (1.3)  F | Family connectedness | Subjective family image test (SFIT) | NR | NR | NR | NR |
| Kast (2016) | USA | Cross-sectional | Students | 4,814  9^th^&12^th^ graders  M&F | Family connectedness | Items from the Minnesota Adolescent Health Survey | NR | NR | NR | NR |
| Katz (2019) | USA | Cross-sectional | Students | 54  16.6  M & F | Peer connectedness | Social Connectedness Survey | NR | NR | NR | NR |
| Keith (1994) | USA | Cohort | Students | 400  14.1  M&F | Peer, family, school, and community connectedness | Quality of Student Life Questionnaire (QSLQ) | 0.90 | 0.90 | NR | NR |
| Kelly (2012) | Australia | Cross-sectional | Adolescents | 7,064  10-14 years  M & F | School connectedness | 7 items | 0.77 | NR | NR | NR |
| Kern (2016) | USA and Australia | Development and validation | Diverse sample of adolescents | 4,480  10-18 years  M&F | Peer, family, school, and community connectedness | EPOCH measure of adolescent wellbeing | 0.65 – 0.89 across samples for the connectedness subscale | 0.36 – 0.55 across samples for connectedness sub-scale | Face validity  Construct validity  Convergent and discriminant validity  Predictive validity | Items at face value represented the 5 factors of the scale  A 5-factor model demonstrated good model fit on CFA: RMSEA = 0.053, SRMR = 0.038, χ2 (160) = 545  Expected correlation between the connectedness sub-scale and measures of wellbeing, psychologic symptoms, life conditions, health and behavior  Connectedness predicted better self-rated academic performance (r = 0.29) and objective language arts test scores (r = 0.25) |
| Kern (2019) | China, USA & Australia | Validation | Students and comparison sample | 6727  14.1 (2.9)  9-19 years  M&F | Peer, family, school, and community connectedness | EPOCH measure of adolescent wellbeing | 0.94 for the overall scale across samples, 0.77 – 0.90 for subscales (omega) | NR | Construct validity  Measurement invariance  Cross-cultural adaptation | A 5-factor structure fit the data well: RMSEA = 0.048, SRMR = 0.053, CFI = 0.960, TLI = 0.952  The 5-factor model showed strict invariance across gender (RMSEA = 0.041, CFI = 0.95, χ2 = 2079.7, df = 370) and age (RMSEA = 0.044, CFI = 0.94, χ2 = 2352.1, df = 370), as well as configural (RMSEA = 0.042, CFI = 0.96, χ2 = 2983.5, df = 480) and weak invariance across countries (RMSEA = 0.045, CFI = 0.95, χ2 = 3476.0, df = 510)  Scale translated to Chinese then back-translated to English by independent translators following translation procedures. Experts reviewed the two versions and agreed that the translated version captured the language and meaning of the original measure |
| Kernsmith (2011) | USA | Cross-sectional | Students | 343  9^th^ -12^th^ graders M&F | School Connectedness | The Measure of School Connection | 0.82 | NR | NR | NR |
| Kerpelman (2005) | USA | Cohort | Students | 188  7^th^-11^th^ graders  F | Family Connectedness | Social Bond Measure | NR | NR | NR | NR |
| Khawaja (2018) | Australia | Cross-sectional | Refugees/ immigrants | 237  11-18 (14.9 (1.7))  M&F | School Connectedness | PSSM | 0.71 | NR | NR | NR |
| Kia-Keating (2007) | USA | Cross-sectional | Adolescent refugees | 76  15.6 (2.0)  M&F | School connectedness | PSSM | NR | NR | NR | NR |
| Kiang (2009) | USA | Cross-sectional | Students from diverse backgrounds | 682  16.0 (1.2)  M&F | Ethnic identity | MEIM | 0.68 – 0.88 for the subscales | NR | NR | NR |
| Kim (2020) | USA | Cohort | Students | 93  15 year olds  M&F | School connectedness | 10 items modified from the high school questionnaire | 0.91 | NR | NR | NR |
| Kim (2019) | USA | Cross-sectional | Students | 1867  9-12 graders  M&F | School Connectedness | Social connectedness scale | 0.81 | NR | NR | NR |
| King (2018) | USA | Trial | Youths seeking emergency medical care | 218  12-15 (13.5 (1.1))  M & F | Family, school, peer, and community connectedness  Community connectedness  Family, school, peer, and community connectedness | UCLA Loneliness Scale-Revised  The Community Connectedness Scale  The Interpersonal Needs Questionnaire-Revised (INQ-R) | 0.81  0.70  0.79 | NR  NR  NR | NR  NR  NR | NR  NR  NR |
| King (2019) | USA | Validation | American Indian and native Alaska adolescents | 12-17 years  M&F | Cultural connectedness | Cultural connectivity scale – California (CCS-CA) | NR | NR | Cross-cultural adaptation  Face validity | A series of FGDs and KIIs (including 2 youth FGDs) helped adapt the scale.  Modifications of the items from the original scale to reflect the current culture were made after these FGDs  Consensus on face validity was reached during the FGDs |
| King (2019) | USA | Cohort | aly representative adolescents | 20,745  15.39 (.13)  M & F | Family connectedness | 4 items | 0.74 | NR | NR | NR |
| King (2009) | USA | Cohort | Culturally and linguistically diverse adolescents | 679  13-16 (14.9 (0.4))  M&F | Family relatedness | FACES II | 0.86 and 0.87 for mother and father relatedness, respectively | NR | NR | NR |
| King (2015) | Philippines | Cross-sectional | Students | 848  14.64 (1.5)  M&F | Peer , school and family relatedness | Relatedness Scale | 0.77, 0.58, and 0.82 for peer, school, and family connectedness, respectively | NR | NR | NR |
| King (2018) | USA | Cross-sectional | Students | 16,684  15.4  M&F | Family Connectedness | 4 items | 0.76 | NR | NR | NR |
| King-Sears (2020) | USA | Cross-sectional | Students with and without disability | 10  12.1 (12.0)  M&F | School connectedness | PSSM | 0.84 | NR | NR | NR |
| Kiracaburun (2018) | Turkey | Cross-sectional | Students | 339  18.0 (2.5)  M&F | Peer, family, school, and community Connectedness | Social connectedness scale | 0.89 | NR | NR | NR |
| Klinck (2020) | USA | Cohort | Students | 1,344  11-14 (12.7 (0.7))  M&F | School connectedness | School connectedness scale | 0.70 at TI &  0.75 at T2 | NR | NR | NR |
| Knight (2007) | USA | Cohort | Patients seeking routine medical care | 305  16.0 (1.8)  M & F | Spiritual connectedness | 15-item Spiritual Connectedness Scale | NR | NR | NR | NR |
| Knight (2018) | Australia | Cross-sectional | Adolescents with Chronic Fatigue Syndrome | 39  13-17  M & F | School connectedness | PSSM | NR | NR | NR | NR |
| Krishnan (2015) | USA | Cross-sectional | Undergraduate students | 674  19 (1.33)  M & F | Family, school, peer, and community connectedness | Measure of Attitudes Toward Social Networking Sites (MATS) | 0.84 for the social connection subscale | NR | Construct validity | EFA yielded a 4-factor solution that accounted for 70.31% of the total variance |
| Kuang (2019) | Taiwan  China  Japan  South Korea | Validation | Students | 36,863  4^th^ or 8^th^ graders  M&F | School connectedness | Sense of School Belonging Scale (SOSB) | 0.82 | NR | Construct validity  Differential item functioning  Content validity | Unidimensional measure with good model fit.  The scale showed neither gender DIF nor grade DIF, while two items showed society DIF  All except one item were judged to be appropriate. This item was subsequently deleted. |
| Kuczynski (2020) | USA | Cross-sectional | Students | 1,456  19.7 (2.4)  M&F | Peer, family, school, and community Connectedness | Social connectedness scale | 0.96 | NR | NR | NR |
| Kumar (2015) | India | Cross-sectional | Students | 100  17-20 years  M&F | Peer, family, school, and community connectedness | Social connectedness scale | NR | NR | NR | NR |
| Kuo (2017) | Taiwan | Cross-sectional | Aboriginal adolescents | 383  15.87 (0.88)  M & F | School connectedness | PSSM  Need to belong scale | 0.88  0.77 | NR | Construct validity | PCA yielded two factors explaining 37.9% of the variance |
| Kuo (2019) | Taiwan | Cross-sectional | Students | 359  15-18  M&F | School connectedness  Ethnic identity | PSSM  MEIM | 0.89  0.88 | NR  NR | NR  NR | NR  NR |
| Kuperminc (2020) | USA | Cross-sectional | Vulnerable high school students | 114  12.8-15.9 (14)  M & F | School connectedness | 5 items | 0.78 | 0.26 – 0.54 | NR | NR |
| Kuperminic (2008) | USA | Cross-sectional | Latino students | 195  13.8  M&F | School Connectedness | PSSM | 0.81 | NR | NR | NR |
| Lahlah (2013) | Netherlands | Cross-sectional | Dutch and Moroccan-Dutch adolescent boys | 364  16.01 (0.91)  M | Family connectedness | 36 items of the Egna Minnen Betraffande Uppfostran for Adolescents (EMBU- A) | NR | NR | NR | NR |
| Lam (2015) | China | Cross-sectional | Students | 406  13.92 (1.32)  M & F | School connectedness | PSSM | 0.65 – 0.85 | NR | Construct validity | PCA yielded two factors explaining 52% of the variance with item loadings > 0.5 |
| Langille (2012) | Canada | Cross-sectional | Students | 408  15-19 years  M&F | School connectedness | School connectedness scale | 0.76 | NR | NR | NR |
| Langille (2014) | Canada | Cross-sectional | Sexually active students | 1,415  15-19 years  M&F | School connectedness | 5 school connectedness scale | 0.82 | NR | NR | NR |
| Lardier (2019) | USA | Cross-sectional | Urban high school students of color | 401  16.55 (1.31)  M & F | School connectedness | Items adapted from the Communities that Care Survey | 0.80 | NR | NR | NR |
| Law (2013) | Australia | Cohort | Students attending eight state schools | 563  9-16.6  M & F | School connectedness  Peer connectedness  Peer connectedness  Family connectedness | PSSM  The Peer Involvement in Delinquent Acts Scale (PIDAS)  The Modiﬁed Friendship Scale (MFQS)  Parental Bonding Instrument | 0.91 - 0.93  0.78 - 0.84  0.88 - 0.91  0.36 - 0.88 for subscales across time points | NR  NR  NR  NR | NR  NR  NR  NR | NR  NR  NR  NR |
| Lazaro-Visa (2019) | Spain | Cohort | Students | 693  10-18  M&F | School Connectedness | School climate and Functioning Scale | 0.73 – 0.79 | NR | NR | NR |
| Leake (2007) | USA | Cross-sectional | Stepfamily adolescents | 60  16.7 (1.69)  M & F | Family connectedness | The Family Belonging Scale-Revised | 0.90 | NR | NR | NR |
| Lee (2014) | USA | Cross-sectional | Students | 3,268  15-year olds  M&F | School connectedness | Items | 0.86 | NR | NR | NR |
| Lee (2017) | South Korea | Cohort | Adolescents | 414  11-12  M&F | School Connectedness | 6 items | 0.85 | NR | NR | NR |
| Lee (2017) | Australia | Development and validation | Student | 7,207  14.0 (1.5)  M&F | School Connectedness | School Climate and School Identification Measure –Student (SCASIM-st)  Perceived School Experiences Scale (PSES) – School Connectedness Subscale  Gottfredson’s Effective School Battery (ESB)  Chicago Public Schools Student Connection Survey  Teacher Academic Support in the Classroom Life Measure | McDonald’s Omega of 0.86 – 0.93 for subscales  0.91  0.57 – 0.76 across subscales  0.63  0.95 | 0.60 – 0.75 across subscales over one month  0.44 – 0.58 across subscales over one year  NR  NR  NR | Content validity  Convergent validity  Construct  validity  Measurement invariance  Criterion validity  NR  NR  NR | The experts’ agreement rate was 75.7% and all items were rated with highest marks (greater than a 2.5 mean)  Significant positive correlations between subscales on the scale and subscales on the Effective School Battery, the Perceived School Experiences Scale, the Chicago Public Schools Student Connection Survey and the Teacher Academic Support in the Classroom Life Measure  EFA yielded a 4-factor structure. CFA confirmed the structure with high factor loadings ranging from 0.89 to 0.96 and good model fit ($\chi^{2}(7, N=1164)$ = 53.84 *p* < 0.001; RMSEA = 0.075; CFI = 0.992; TLI = 0.984)  Configural, metric, and scalar measurement invariance established across gender, age, and language groups  Students' school climate or school identification as measured by the SCASIM-st was associated with academic achievement, school attendance, aggressive behaviour at school, and depression  NR  NR  NR |
| Leemis (2019) | USA | Cohort | Racially diverse students | 3,549  12.8 (1.1)  M&F | School connectedness | 4 items from PSSM | 0.77 | NR | NR | NR |
| Legette (2020) | USA | Cohort | Students | 322  11-13  M&F | School connectedness | 5 items | 0.83 at TI and 0.85 at T2 | NR | NR | NR |
| Lemberger (2012) | USA | Trial | Students | 100  4^th^&5^th^ graders  M&F | School connectedness | Child and adolescent social support scale – people in my school subscale | 0.95 and 0.94 across time points | NR | NR | NR |
| Lenzi (2013) | Italy | Cross-sectional | Early-and middle-adolescents | 403  11-15 (13.6 (1.6))  M&F | Community connectedness  Peer connectedness | Neighborhood Intergenerational Closure Scale and Sense of Community Scale for Adolescents (SOC-A)  Sense of Community Scale for Adolescents (SOC-A) | 0.78 and 0.82  0.91 | NR  NR | NR  NR | NR  NR |
| Lenzi (2013) | Italy | Cross-sectional | Early- and middle-adolescents | 403  11-15 (13.6 (1.64))  M & F | Peer connectedness  Community connectedness | Friendship subscale of the Neighborhood Youth Inventory  Neighborhood Intergenerational Closure scale | 0.91  0.78 | NR  NR | NR  NR | NR  NR |
| Leon (2017) | Spain | Cross-sectional | Students | 1964  15.0 (1.52)  M&F | Peer relatedness  School relatedness | 5 items  5 items | 0.93  0.88 | NR  NR | Construct validity  Construct validity | Factor loadings ranging  between 0.54 and 0.86 with  good model fit statistics: χ2 (1961, 5) = 103.14 (p = 0.00), RMSEA = 0.10, CFI = 0.99 and TLI = 0.98.  Factor loadings ranging between 0.72 and 0.93 with good model fit: χ2 (1961, 5) = 92.15 (p =0.00), RMSEA=0.09, CFI=0.99, TLI=0.98 |
| Lester (2013) | Australia | Cohort | Students | 3,459  12-14 years  M&F | School connectedness | School connectedness scale | 0.80 | NR | Construct validity | Unidimensionality was conﬁrmed through factor analysis (CFI > 0.9, SMR < 0.10 at all-time points) |
| Lewis (2006) | USA | Trial | Students | 65  13.3 (0.5)  M&F | School connectedness | PSSM | 0.88 – 0.95 across time points | NR | NR | NR |
| Li (2013) | China | Cross-sectional | Students | 2,758  13.5 (1.1)  M&F | School connectedness | School connectedness scale | 0.85 | NR | Construct validity  Convergent validity | 1-factor structure on CFA  Significant and positive correlations with perceptions of teacher support, student support, academic achievement and negative correlation with depression |
| Li (2018) | China | Cross-sectional | Students | 1,898  13.6 (1.2)  M&F | School connectedness | 3 items | NR | NR | NR | NR |
| Li (2020) | China | Cross-sectional | Rural adolescents | 813  15-year olds  M&F | School connectedness | PSSM | 0.82 | NR | NR | NR |
| Li (2018) | China | Cross-sectional | Adolescents | 136  13-18 (15.3 (1.5))  M&F | Family Relatedness | Parental Bonding Instrument | NR | NR | NR | NR |
| Liau (2015) | Singapore | Cohort | Students | 2,998  3^rd^,4^th^,7^th^ & 8^th^ graders  M&F | Family connectedness | Parent-family connectedness scale | 0.89 | NR | NR | NR |
| Little (2005) | USA | Cohort | Adolescent whose mothers had a history of depression | 185  11.8 (0.6)  M&F | Peer, family, school, and community connectedness | Depressive experiences questionnaire for adolescents (DEQ-A) – connectedness and neediness subscales | 0.75 for connectedness subscale and 0.66 for neediness sub-scale | NR | NR | NR |
| Liu (2016) | China | Cross-sectional | Chinese migrant adolescents | 798  13.10 (1.48)  M & F | Ethnic identity | MEIM | 0.78 | NR | NR | NR |
| Liu (2016) | China | Cross-sectional | Students | 1407  10.08-17.42 (12.74 (0.57))  M & F | School connectedness | School Engagement Scale | 0.79 | NR | NR | NR |
| Lohmeier (2011) | USA | Validation | Students | 929  9^th^-12^th^ graders  M&F | School Connectedness | 51-item Parker’s School Connectedness Scale | 0.93 & 0.81 across schools; 0.78 for the combined sample | NR | Construct validity | A 7-factor solution accounting for 44.13% of the variance on EFA demonstrated the best fit to the data |
| Lombardi (2019) | USA | Cohort | HIV exposed adolescents | 18,921  12-18 (16.0 (1.8))  M&F | Family Connectedness  Peer connectedness  School connectedness | 8 items  5 items  5-item school connectedness scale | 0.67  0.93  0.77 | NR  NR  NR | NR  NR  NR | NR  NR  NR |
| Lombas (2019) | Spain | Trial | Students | 524  13.6  M&F | Peer Connectedness | Psychological needs Satisfaction Scale in Education | NR | NR | NR | NR |
| López (2012) | USA | Cross-sectional | Young Mexican athletes | 669  13.95  M & F | Peer relatedness | Need for Relatedness Scale | 0. 87 | NR | Construct validity | The factors extracted from EFA and CFA explained 65.34% of the variance |
| Loukas (2009) | USA | Cohort | Students | 500  11.7 (0.8)  M&F | School connectedness | School connectedness scale | 0.76 at T1and 0.77 at T2 | NR | NR | NR |
| Lubans (2016) | Australia | RCT | Adolescent boys | 361  12.7 (.5)  M | School relatedness | The Basic Needs Satisfaction in Sport Scale | 0.84 | NR | NR | NR |
| Luyckx (2009) | Belgium | Cross-sectional | Students | 714  16-20 (18.0 (0.6))  M&F | Family, school, peer, and community relatedness | Need satisfaction scale | 0.77 – 0.81 across samples and time points | NR | Construct validity | 3-factor structure showed adequate model fit on CFA: RMSEA = 0.08, CFI = 0.96, SRMR 0.06 |
| Lynch (2002) | USA | Cross-sectional | Adolescents from low-income urban neighborhoods | 127  10.1 (1.8)  M & F | Family relatedness | Relatedness questionnaire | NR | NR | NR | NR |
| Lyons (2019) | USA | Cross-sectional | Students | 2670  11 (2.4)  M & F | School connectedness | School Bonding scale | NR | NR | NR | NR |
| Ma (2017) | USA | Cross-sectional | Hispanic Adolescents | 225  13-16 (14.42 (1.08))  M & F | Ethnic identity  Family connectedness | 3 items  7 items | 0.70  0.84 | NR  NR | NR  NR | NR  NR |
| Madil (2014) | USA | Cross-sectional | Students | 628  1^st^,3^rd^ & 5^th^ graders  M&F | School relatedness  Peer relatedness | Student-teacher relationship scale – closeness sub-scale  Sense of community scale | 0.84 at T1 and 0.89 at T2  0.83 at T1 and 0.82 at T2 | 0.79 and 0.65 (8 weeks)  0.78 and 0.63 | NR  NR | NR  NR |
| Mahabee-Gittens (2011) | USA | Cross-sectional | Adolescents presenting to an emergency department | 272  9-16 years  M&F | Family connectedness | Multitrait-multimethod model | 0.59 – 0.89 across sub-scales | NR | NR | NR |
| Mahabee-Gittens (2011) | USA | Cohort | Students | 4,061  14.4  M & F | Family connectedness | Family connectedness scale | 0.83 | NR | NR | NR |
| Mahabee-Gittens (2012) | USA | Cohort | Students | 6426  9-18  M & F | Family Connectedness | 3 items | 0.78 | NR | NR | NR |
| Malafaia (2016) | Portugal | Cross-sectional | Students | 732  Grades 8 and 11  M & F | School connectedness | 6 items | 0.78 | NR | NR | NR |
| Malaquias (2015) | Portugal | Cross-sectional | Adolescents | 248  15-20 (16.27 (1.22))  M&F | Peer, family, school, and community connectedness | Social Connectedness Scale—Revised | 0.90 | NR | NR | NR |
| Mallett (2011) | USA | Cross-sectional | Students | 546  12-19 (16 (1.25))  M&F | Ethnic identity  School connectedness | MEIM  1 item | 0.78 –0.80  0.86 | NR  NR | NR  NR | NR  NR |
| Mameli (2018) | Italy | Cross-sectional | Students | 540  13-70 (14.9)  M&F | School connectedness | Connected classroom climate inventory | 0.92 | NR | NR | NR |
| Mandara (2009) | USA | Cross-sectional | African American students | 259  12-14 (12.6 (0.7))  M&F | Racial identity | MEIM | 0.80 and 0.73 across groups | NR | NR | NR |
| Mann (2015) | USA | Trial | Female students | 48  12-14 years  F | School connectedness  Self-connectedness | School connectedness scale  Adolescent personality style inventory – identity subscale | 0.82  0.84 | NR  NR | NR  NR | NR  NR |
| Mao (2012) | China | Cross-sectional | Internal migrants and local adolescents | 1,015  11-17 (13.6 (1.1))  M&F | School connectedness  Peer connectedness  Family connectedness  Community connectedness | School Adaptation Scale  School Adaptation Scale  FACES-II  Social Support Rate Scale | 0.71  0.82  NR  0.76 | NR  NR  NR  NR | NR  NR  NR  NR | NR  NR  NR  NR |
| Marino (2020) | Italy | Cross-sectional | Students | 209  11-13  M & F | School connectedness | HMAC | 0.74 and 0.83 across time points | NR | NR | NR |
| Markham (2003) | USA | Cross-sectional | Sexual risk-taking students | 976  9^th^ -10^th^ graders  M & F | Family connectedness | 14 items | 0.87 | NR | NR | NR |
| Marsh (2020) | USA | Development and validation | Students with behavioral & emotional disorders (EBD) and comparison peers | 200  6-18 years  M&F | School connectedness | School Connectedness Questionnaire | 0.64 – 0.76 across sub-scales in the EBD group  0.51 – 0.80 across sub-scales in the comparison peers | NR | Content validity  Construct validity | Items on the final version of the scale were based on feedback from experts who assessed the scale’s appropriateness, length of items, readability of items and any other relevant aspect  3-factor structure accounting for 62.81% and 59.25% of the variance in EBD and comparison peers, respectively |
| Marshik (2017) | USA | Cohort | Students | 10,395  11 and 11.5  M & F | Peer relatedness | Peer scale of the Self-Description Questionnaire | NR | NR | NR | NR |
| Marsiglia (2019) | Uruguay | Cross-sectional | Students | 353  12.39 (.65)  M & F | Peer connectedness | 1 item | NR | NR | NR | NR |
| Marta-Simoes (2020) | Portugal | Cross-sectional | Students | 362  12-18 (14.3 (1.5))  M&F | Peer, family, school, and community connectedness | Social safeness and pleasure scale | 0.94 | NR | NR | NR |
| Martin-Albo (2015) | Spain | Cross-sectional | Students | 703  12-17 (14.0 (1.4))  M&F | School relatedness | Psychological needs scale | NR | NR | NR | NR |
| Martinez-Fuentes (2020) | USA | Cross-sectional | Self-identified Latino adolescents | 370  14-18 (16.14 (1.12))  M & F | Ethnic identity | Ethnic Identity Scale | NR | NR | NR | NR |
| Marx (2019) | USA | Cross-sectional | Transgender and gender non-conforming adolescents | 610  14-18 (15.7 (1.2))  M&F | School connectedness | 6 items | 0.85 | NR | NR | NR |
| Master (2016) | USA | Cross-sectional | Students | 165  14-18 (16.0 (1.2))  M&F | School connectedness | 4 items | 0.94 and 0.92 for both classrooms | NR | NR | NR |
| Matlin (2011) | USA | Cross-sectional | African American adolescents | 212  13-19 (15.5 (1.3))  M&F | Community connectedness | Collective Efficacy Scale – Social Cohesion Subscale | 0.72 | NR | NR | NR |
| Matteau‐Pelletier (2020) | Canada | Cohort | Students | 6185  7^th^-11^th^ graders  M & F | School connectedness | Items from Student Tobacco, Alcohol and Drugs Survey | 0.79 | NR | NR | NR |
| Maurizi (2013) | Chile | Cohort | General adolescent population | 894  12-17 (14.4)  M&F | School connectedness | 8 items | 0.80 | NR | NR | NR |
| Maurizi (2013) | USA | Cross-sectional | Latino adolescents residing in low-income urban neighborhood | 202  14.5 (0.69)  M & F | Community connectedness  School connectedness | 10 items adapted the school belonging measure  9 items adapted from PSSM and Perceived Cohesiveness scale | 0.87  0.85 | NR  NR | NR  NR | NR  NR |
| McDermot (2014) | Australia | Cohort | Student in Cyclone affected area | 371  13.2 (1.0)  M&F | Peer, family, school, and community Connectedness | Australian Community Participation Questionnaire | NR | NR | NR | NR |
| McCallum (2011) | UK | Cross-sectional | Adolescents | 99  10-14 (16.07 (1.04))  M & F | Community Connectedness | Sense of Belonging Instrument – Psychological Subscale (SOBI-P) | 0.90 – 0.95 | NR | NR | NR |
| McCue (2019) | UK | Cross-sectional | General adolescent population | 175  11.7-18.5 (14.8 (1.8)  M&F | Self-connectedness | A measure adapted from previous studies | 0.64 | NR | NR | NR |
| McDavid (2014) | USA | Cohort | Early adolescence | 134  10-13  M & F | Peer relatedness | Need for Relatedness Scale | NR | NR | NR | NR |
| McDermott (2020) | UK | Development and validation | Students | 722  Year 8  M&F | School connectedness | New 20-item School Belonging Scale | 0.69 – 0.87 across sub-scales | NR | Construct validity  Divergent validity | -Inter-scale correlations, which were all statistically significant (p < 0.01) and of weak-to-moderate (0.305 to 0.554) magnitude  - A 4-factor solution was derived from factor analysis explaining approximately 49% of the variance with item loadings above 0.32 and minimal item cross loadings (i.e. <.40)  Scale measured distinct dimensions of connectedness |
| McDonough (2016) | New Zealand | Cohort | Students | 1,940  10-15 (12.2 (1.8))  M&F | Peer connectedness | 7 items | 0.89, 0.89, and 0.88 across time points | NR | NR | NR |
| McGraw (2008) | Australia | Cohort | Students | 941  16-19 (17.4 (0.6))  M&F | School connectedness  Peer connectedness  Family Connectedness | PSSM  Revised UCLA Loneliness Scale  Parent-family connectedness scale | 0.90  0.92  0.90 | NR  NR  NR | NR  NR  NR | NR  NR  NR |
| McKinney (2011) | USA | Cross-sectional | Culturally diverse students | 710  11-14 (12.3 (0.9))  M&F | Family connectedness | FACES-II | 0.78 | NR | NR | NR |
| McLaren (2015) | Australia | Cross-sectional | General adolescent population | 40  14-18 (16.5 (1.1))  M&F | Peer connectedness  School connectedness  Community Connectedness | Social Questionnaire for Secondary students  Social Questionnaire for Secondary students  Psychological subscale of the sense of belonging instrument – Psychological Subscale (SOBI-P) | 0.83  0.93  0.90 | NR  NR  NR | NR  NR  NR | NR  NR  NR |
| McLoughlin (2019) | Australia | Cross-sectional | Students | 229  12-17 (14.0 (1.2))  M&F | Peer, family, school, and community connectedness | Social connectedness scale | 0.91 | NR | NR | NR |
| McMahon (2004) | USA | Cohort | Urban youth | 431  10-15 years  M & F | School connectedness | PSSM | 0.77 – 0.88 | NR | NR | NR |
| McMahon (2008) | USA | Cross-sectional | Low-income urban youth with disabilities | 136  11-20 (17.0)  M&F | School connectedness | PSSM | 0.88 | NR | NR | NR |
| McMahon (2020) | Australia | Cohort | Disengaged Adolescents | 40  16-17  M&F | Family, school, peer, and community Relatedness | The basic measure of psychological needs | 0.77 – 0.82 | NR | NR | NR |
| McNeely (2002) | USA | Cohort | Students | 75,515  7^th^-10^th^ graders  M & F | School connectedness | School connectedness scale | 0.79 | NR | NR | NR |
| McWhirter (2011) | Chile | Validation | General adolescent population | 390  12-19 (15.5 (1.8))  M&F | Self, peer, family, school, and community connectedness | HMAC | 0.63 – 0.96 across the subscales | NR | Construct validity  Content validity  Concurrent and discriminant validity | EFA revealed a 11-factor structure accounting for 61.92% of the total variance  Researchers and a psychologist judged the items to be clear and comprehensive  Expected correlations with youth, parent and teacher-reported youth risk behaviors and positive family relations |
| McWhirter (2018) | USA | Cross-sectional | Students | 896  14-19 (16.6 (1.2))  M&F | School connectedness | HMAC | 0.80 | NR | NR | NR |
| Meca (2020) | USA | Cohort | Latino adolescents | 1,613  14.0 (0.4)  M&F | Ethnic identity | MEIM – exploration and belonging subscales | 0.85 and 0.92 for both sub-scales, respectively | NR | Measurement invariance | Good model fit for configural (χ2 (543) = 2132.620, p < 0.001; CFI = 0.920, RMSEA = 0.042; metric (Δχ2 (24) = 37.676, p = 0.037, ΔCFI < 0.001, ΔRMSEA < 0.001 and scaler longitudinal invariance (Δχ2 (24) =135.679, p < 0.001; ΔCFI = 0.006; ΔRMSEA < 0.001 |
| Meisel (2017) | USA | Cohort | Substance abusing adolescents | 387  11–16  M & F | School connectedness | School connectedness scale | 0.75 – 0.81 | NR | NR | NR |
| Mellin (2002) | USA | Cross-sectional | Overweight adolescents | 9,957  14.4 (2.9)  M&F | Family connectedness | Family connectedness scale from the Voice of Connecticut Youth Survey | NR | NR | NR | NR |
| Menendez-Santurio (2016) | Spain | Cross-sectional | Students | 143  14-17 (15.4 (0.8))  M&F | School relatedness | Basic psychological needs in physical education scale – relatedness sub-scale | NR | NR | NR | NR |
| Mengya (2016) | USA | Cohort | Rural adolescents | 979  11.3 (.49)  M & F | School connectedness | Loneliness scale | 0.92 – 0.94 | NR | Convergent validity | Correlations for these scales ranged from 0.42 to 0.49 across time points |
| Merrit (2015) | USA | Cross-sectional | Maltreated adolescents | 1,054  11-17  M&F | Peer Connectedness | Loneliness and Social Dissatisfaction Questionnaire for young children | 0.88 | NR | NR | NR |
| Mesch (2003) | Israel | Cross-sectional | Adolescents | 909  14-18  M & F | Family connectedness  Peer connectedness | 3 items  3 items | 0.815  0.658 | NR  NR | NR  NR | NR  NR |
| Milam (2017) | USA | Cross-sectional | Students | 27,697  16.1 (1.2)  M&F | School connectedness | 4 items from the school climate survey | 0.83 | NR | NR | NR |
| Mildford (2016) | Australia | Cohort | Students | 56  13.5  M&F | School Connectedness | Class connectedness scale | NR | NR | NR | NR |
| Miller (2017) | USA | Trial | Students | 627  10.0 (0.4)  M&F | School Connectedness | School Liking and Avoidance Questionnaire | 0.94 | NR | NR | NR |
| Millings (2012) | UK | Cross-sectional | Adolescents with depression | 5022  11-16  M & F | School connectedness | PSSM | NR | NR | NR | NR |
| Milyavskaya (2009) | Canada  USA  France  China | Cross-sectional | Students | 2,369  11-18 years  M&F | Peer, family, and school connectedness  Peer and school relatedness | Children’s intrinsic needs satisfaction scale –relatedness subscale  Basic need satisfaction at work scale | 0.80 for peer, 0.74 for family, 0.73 for school relatedness  0.84 | NR  NR | NR  NR | NR  NR |
| Minnaert (2007) | Netherlands | Cohort | Students | 114  15-17  M&F | Peer, family, school, and community Connectedness | Social relatedness Scale | 0.70 | NR | Construct validity | CFA yielded good indices of fit, with overall GFI, IFI, and CFI goodness-of-fit measures ranging between 0.94 and 0.97 |
| Mohatt (2011) | USA | Development and validation | Alaska native youth | 284  12-18  M & F | Family, self, and community connectedness  Cultural connectedness | Awareness of Connectedness Scale (ACS)  Alaska Native Cultural Identification (ANCI) | 0.85 for overall scale  0.54 – 0.80 for subscales  NR | NR  NR | Construct validity  Convergent validity  Discriminant validity  NR | 4-factor structure with good model fit: χ2 (47) = 79.5, p < 0.01, χ2/df = 1.69, GFI = 0.96, CFI = 0.97  Moderate to strong correlations with measures of reasons for living, cultural identification, and mastery  Low correlations with and some subscales in these measures  NR |
| Mok (2016) | Germany | Cross-sectional | Turkish-Origin and German Students | 9215  15.23 (0.65)  M & F | School connectedness | 8 items from the National Assessment Study | 0.80 | NR | NR | NR |
| Moller (2010) | USA | Cross-sectional | Undergraduates | 28918  18-23  M & F | Self-relatedness | Basic Psychological Needs Scale | NR | NR | NR | NR |
| Morgan (2009) | UK | Cross-sectional | Students | 6425  11-15  M & F | School connectedness  Family connectedness  Community connectedness | 3 items  4 items  3 items | NR  NR  NR | NR  NR  NR | NR  NR  NR | NR  NR  NR |
| Morin (2015) | USA | Cross-sectional | Adolescents | 28,104  9^th^-12^th^ graders  M & F | School connectedness | Items from Add Health survey, Healthy Kids Survey and School Development Program School Climate Surveys | 0.86 | NR | NR | NR |
| Moscardino (2014) | Russia | Cross-sectional | Adolescents exposed to terrorism | 60  14-18 (15.4 (1.0))  M&F | School connectedness | School connectedness scale | 0.71 | NR | NR | NR |
| Moscardino (2010) | Russia | Cross-sectional | Terrorist attack Survivors | 158  14-17  M&F | Community Connectedness | Sense of Community Index | 0.71 | NR | NR | NR |
| Mrug (2009) | USA | Cohort | African Americans | 594  11.3 (0.5)  M & F | School connectedness | 8 items from the Attitudes Toward School Scale and the  School connectedness scale | 0.77 | NR | NR | NR |
| Mueller (2011) | USA | Cohort | Students | 3,177  12-21 (16 (1.74))  M & F | Family connectedness | 3 items adapted from the parent-family connectedness scale | 0.79 | NR | Construct validity | One-factor structure accounting for 65.39% of the variance |
| Murdock (2005) | USA | Cross-sectional | Lesbian, gay & bisexual students | 101  High school youth  M&F | School connectedness | PSSM | 0.87 | NR | NR | NR |
| Murnaghan (2012) | Canada | Cross-sectional | Students | 10,632  5^th^-12^th^ graders  M&F | School Connectedness | School connectedness scale | NR | NR | NR | NR |
| Murphy (2016) | Australia | Cross-sectional | Preadolescents | 75  10 -12 (10.84 (0.66))  M & F | School connectedness | PSSM | 0.87 | NR | NR | NR |
| Murray (2006) | USA | Cohort | Students | 96  11.5  M&F | School Connectedness | Social Competence rating Scale for Children | 0.80 | NR | NR | NR |
| Murray-Harvey (2009) | Australia | Cross-sectional | Students | 888  10-16 (12.9)  M&F | School connectedness | 7 items | 0.77 | NR | NR | NR |
| Nelson (2018) | USA | Cross-sectional | African American adolescents | 198  11-19 years  M&F | Cultural connectedness | Acculturation, habits, and interests multicultural scale for adolescents (AHIMSA) | 0.87 | NR | NR | NR |
| Neto (2010) | Portugal | Cross-sectional | Immigrant adolescents | 755  15.54 (2.06)  M & F | Cultural connectedness | MEIM | 0.81 – 0.89 | NR | NR | NR |
| Neumark-Sztainer (2009) | USA | Cohort | Overweight adolescents | 412  12.8 (0.8)  M&F | Peer connectedness  Family connectedness | 1 item  3 items | NR  0.69 | NR  NR | NR  NR | NR  NR |
| Newman (2007) | USA | Cross-sectional | General adolescent population | 733  11-18 (14.7 (1.9))  M&F | Peer connectedness | Group belonging scale | 0.80 | NR | NR | NR |
| Nichols (2006) | USA | Cross-sectional | Students | 45  7^th^ & 8^th^ graders  M&F | School connectedness | PSSM | 0.82 | NR | NR | NR |
| Nichols (2008) | USA | Cross-sectional | Students | 9,063  12-18 (15.9 (0.03))  M&F | School connectedness  Family connectedness | School connectedness scale  Parent-family connectedness scale | NR  NR | NR  NR | NR  NR | NR  NR |
| Nickerson (2010) | USA | Cohort | Students | 130  15.5  M&F | School Connectedness | School connectedness scale | 0.77 – 0.84 | NR | NR | NR |
| Niehaus (2012) | USA | Cohort | Students | 330  6^th^ graders  M & F | School connectedness | Items adapted from National Educational Longitudinal Study, the Need Satisfaction Scale and the Scale of Caring Adult  Relationships in School | 0.67 – 0.83 | NR | Construct validity | The two-factor solution, which explained 41% of the variance, was deemed conceptually and structurally more tenable. |
| Niehaus (2016) | USA | Cohort | Latino youth | 1743  10^th^ graders  M & F | School connectedness | ELS:2002 base year student questionnaire | 0.73 | NR | Construct validity | EFA resulted in a 9-item 2-factor solution: χ2 (19) = 963.506 (*p* < 0.001), CFI = 0.930, RMSEA = 0.066 |
| Niolon (2015) | USA | Cohort | Students | 88  15.8 (0.9)  M&F | Family Connectedness | Autonomy and relatedness Coding System | NR | NR | NR | NR |
| Niwa (2014) | USA | Cohort | Ethnic minority adolescents | 585  11.8  M&F | School Connectedness | PSSM | 0.89 | NR | NR | NR |
| Norwalk (2016) | USA | RCT | Early adolescents | 1264  6^th^ graders  M & F | School connectedness | PSSM | 0.83 |  | Predictive validity | School belonging in the Fall was statistically significantly predictive of belonging in the Spring (t = 20.55, p = 0.000). |
| Ntoumanis (2005) | UK | Development and validation | Athletes | 1530  12-16  M&F | Peer relatedness | Peer Motivational Climate in Youth Sport Questionnaire (peerMCYSQ) – relatedness support sub-scale | 0.84 | 0.77 | Content validity  Construct validity | Experts reviewed the items for clarity, age appropriateness, and content. Relevant adjustments were made based on their feedback. Further adjustments were made based on the feedback of a pilot sample of adolescents.  EFA suggested a 6-factor solution. CFA showed that a 5-factor solution had a better model fit |
| Nyberg (2019) | Sweden | Cohort | General adolescent population | 1083  16.0  M&F | School connectedness | 6 items | NR | NR | Predictive validity  Construct validity | Lower scores predicted anxiety and depression at age 30 and 43  RMSEA 0.068, CFI 0.953, TLI 0.922, and SRMR 0.032 |
| O’Brennan (2010) | USA | Cross-sectional | Students | 1,253  12-18  M&F | School connectedness | School connectedness scale | 0.81 | NR | NR | NR |
| O’Neal (2016) | USA | Cross-sectional | Students | 1,202  9-11  M&F | School connectedness | 3-item School belonging scale from the Programme for International Student Assessment (PISA) | 0.79 | NR | NR | NR |
| Oberle (2011) | Canada | Cohort | General adolescent population | 1,943  12.0 (0.5)  M&F | Peer connectedness | Relational provisional loneliness questionnaire – peer belonging subscale | 0.81 | NR | NR | NR |
| Okamoto (2010) | USA | Cross-sectional | Students | 49  11.9 (0.9)  M&F | Cultural connectedness | Hawaiian culture scale – activities and social events subscale | NR | NR | NR | NR |
| Oldfield (2016) | UK | Cross-sectional | Students | 203  11-16  M & F | School connectedness | PSSM | NR | NR | NR | NR |
| Oldfield (2018) | Guatemala | Cross-sectional | Adolescents at risk of drug abuse, homelessness, poverty, and neglect | 90  M&F | School connectedness | PSSM | 0.78 | NR | NR | NR |
| Oman (2013) | USA | Cohort | Youth sexual behaviors | 1089  14.2 (1.6)  M & F | School connectedness | 4 items | 0.56 | NR | NR | NR |
| Omiya (2020) | Japan | Cross-sectional | Students with Autism Spectrum Tendency | 203  7^th^-9^th^ graders  M & F | School connectedness | PSSM | 0.84 | NR | NR | NR |
| Opperman (2015) | USA | Cross-sectional | Adolescents with elevated suicide risk | 129  12-15 (13.6 (1.1))  M&F | School connectedness  Peer connectedness  Family connectedness  Self-connectedness | School connectedness scale  HMAC  Parent-family connectedness scale  UCLA loneliness scale | 0.83  0.84  0.88  0.79 | NR  NR  NR  NR | NR  NR  NR  NR | NR  NR  NR  NR |
| Otsuki (2009) | USA | Cross-sectional | Asian Minority | 65  19.41  M&F | Peer, family, school, and community Connectedness | Revised Social Connectedness Scale | 0.93 | NR | NR | NR |
| Oulali (2019) | Netherlands | Development and validation | Students | 1437  11.72 (0.61)  M & F | Religious identity | Religious Collective Self-Esteem Scale (RCSES) | 0.80 for the overall scale  0.60 – 0.77 for subscales | 0.57 for overall scale  0.36 – 0.58 for subscales | Construct validity  Convergent validity  Discriminant validity  Incremental validity  Measurement Invariance | A 3-factor solution fitted the data best: CFI = 0.95, TLI = 0.92, SRMR = 0.05, RMSEA = 0.05  Small to moderate correlations with measures of individual self-esteem (0.29) and ethnic collective self-esteem (0.40)  Small correlations with school wellbeing (0.18) and social school motivation (0.19)  The scale, including its subscales, significantly predicted, over and above PESES, school wellbeing and school motivation scores  Partial measurement and structural invariance across religious groups established |
| Pacewicz (2020) | USA | Cross-sectional | Female athletes | 276  14.9 (1.3)  F | School relatedness | Basic Needs Satisfaction in Sport Scale (BNSSS) | 0.76 | NR | NR | NR |
| Padilla-Walker (2011) | USA | Cohort | Adolescents | 489  11.9 (0.9)  M&F | Family Connectedness | Social connectedness scale | 0.78 | NR | NR | NR |
| Pallock (2006) | USA | Cross-sectional | Students | 104  14.9 (0.7)  M&F | School connectedness | Teacher bonding scale | 0.65 and 0.66 | NR | NR | NR |
| Parr (2020) | Australia | Cross-sectional | Students | 502  13-17 (14.9 (0.9))  M&F | School connectedness  Community Connectedness | PSSM  Sense of Belonging Instrument - Psychological Subscale (SOBI-P) | 0.93  0.94 | NR  NR | NR  NR | NR  NR |
| Pate (2017) | USA | Cohort | Students | 7,276  13-16 (14.7 (1.1))  M&F | School Connectedness | School connectedness scale | 0.78 | NR | NR | NR |
| Pauwels (2010) | Sweden | Cross-sectional | Students | 1003  12-17  M & F | School connectedness | 9 items | 0.65 – 0.70 | NR | NR | NR |
| Pedreño (2015) | Spain | Cross-sectional | Soccer players | 264  14-16  (14.74 (0.77)  M & F | Peer relatedness | Psychological Need Satisfaction in Exercise Scale (PNSE) | 0.75 | NR | NR | NR |
| Perales (2020) | Australia | Cohort | Sexual minority and different-sex-attracted adolescents | 3,204  14-15 (14.9)  M&F | School connectedness | PSSM | 0.87 | NR | NR | NR |
| Pereira (2020) | Portugal | Trial | Adolescents in residential childcare | 89  7-17 (13.8 (2.6))  M&F | Peer, family, school, and community connectedness | Social connectedness scale - revised | 0.86 pretest and 0.84 posttest | NR | NR | NR |
| Pérez-González (2017) | Spain | Cross-sectional | Sexually victimized adolescents | 1105  12-17 (14.52 (1.76))  M & F | School, family, peer and community connectedness | The Adolescent Resilience Questionnaire | 0.71 – 0.84 | NR | NR | NR |
| Perry (2018) | USA | Cohort | Students | 175  15.8 (1.3)  M&F | School Connectedness | PSSM | 0.85 | NR | NR | NR |
| Pesa (1999) | USA | Cohort | Female adolescents | 2,236  15.9  F | School connectedness  Family connectedness  Community connectedness | 4 items  4 items  Neighbors and sense of community sub-scales | NR  NR  NR | NR  NR  NR | NR  NR  NR | NR  NR  NR |
| Peters (2019) | USA | Cross-sectional | Students | 147  11.17 (0.423)  M & F | School connectedness  Family, self, and community connectedness | PSSM  Awareness of Connectedness Scale (ACS) | NR  NR | NR  NR | NR  NR | NR  NR |
| Petrillo (2016) | Italy | Validation | Students | 390  13-20 (16.4 (1.8))  M&F | School, peer and community connectedness | Classroom sense of community scale (SoC-C) | 0.66 – 0.89 across the subscales | NR | Construct validity  Convergent validity  Measurement invariance | Factor analysis identified and confirmed a 5-factor structure with good model fit: X2/df = 704.74 (289), p < 0.001; X2/df = 2.44, RMSEA = 0.06, SRMR = 0.05, CFI = 0.98, NNFI = 0.98; AIC = 828.74  The scale and its subscales showed significant positive correlations with related measures as expected  Good model fit for configural (X2 = 1125.16  (578), p< 0.000; X2/df = 1.95; CFI = 0.99; NNFI = 0.99; RMSEA = 0.07; SRMR = 0.06; AIC = 1377.51 and metric invariance across gender (X2 =1160.14 (604), p< 0.001; X2/df = 1.92; CFI = 0 .97; NNFI = 0.96, RMSEA= 0.07; SRMR= 0.06; AIC = 1373.16) |
| Pharo (2011) | New Zealand | Cross-sectional | Students | 80  13-22 (15.7 (1.0))  M&F | Peer connectedness | 4-item measure of peer affiliation adapted from a previous study | NR | NR | NR | NR |
| Philipson (2016) | Australia | Cohort | Students | 3,956  12.4 (0.5)  M&F | School connectedness | PSSM | 0.99 | NR | NR | NR |
| Phinney (1992) | Development and validation | Cross-sectional | Students | 417  14-19 (16.5)  M&F | Ethnic identity | MEIM | 0.81 for the overall scale, 0.69 – 0.75 for subscales | NR | Construct validity  Convergent validity | EFA revealed a 2-factor structure accounting for 29.1% of the total variance  Ethnic identity showed significant positive correlation with self-esteem (r = 0.31 & 0.67 for ethnic minority and white students, respectively) |
| Pierre (2020) | USA | Cross-sectional | African American adolescent males | 119  14-18 (15.3 (1.0))  M | School connectedness | PSSM | 0.84 at TI, 0.73 at T2 | NR | NR | NR |
| Pikulski (2020) | USA | Cross-sectional | Clinically anxious youth | 114  10.82 (2.93)  M & F | School connectedness | School connectedness  scale | 0.79 | NR | NR | NR |
| Pina-Watson (2020) | USA | Cross-sectional | Mexican descent adolescents | 722  14-25 (19.7 (1.7))  M&F | Family connectedness | Parent-family connectedness scale | 0.93 – 0.97 across sub-scales | NR | NR | NR |
| Pina-Watson (2015) | USA | Cohort | Latina Adolescents | 276  13-18 (15.7 (1.6)))  M&F | Family Connectedness | 10 items | 0.85 for mother and 0.83 for father connectedness | NR | NR | NR |
| Pinquart (2002) | Germany | Cross-sectional | General adolescent population | 90  11-16 (14.1)  M&F | Family connectedness | Hofer Connectedness Scale | NR | NR | NR | NR |
| Piotrowski (2013) | Poland | Cross-sectional | General adolescent population | 540  14.8 (0.5) – 17.7 (0.9)  M&F | Self-connectedness | Dimensions of identity development scale | 0.69 – 0.88 across sub-scales | NR | NR | NR |
| Pizmony-Levy (2016) | US and Israel | Cross-sectional | LGBT | 5,650  15.9  M&F | School Connectedness | PSSM | NR | NR | NR | NR |
| Podlogar (2017) | Slovenia | Validation | Students | 307  12-17 (14.1 (.96))  M & F | Family, school, peer, and community connectedness | Interpersonal Needs Questionnaire (INQ) –Thwarted belongingness subscale | 0.85 – 0.86 | NR | Construct  Validity  Convergent validity  Cross-cultural adaptation | A 2-factor structure with modifications fit the data well: RMSEA= 0.045, CFI = 0.970 TLI = 0.963, SRMR = 0.036  Thwarted belongingness and Perceived Burdensomeness scores were associated with concurrent and follow-up suicide ideation and lifetime history of suicide attempt, as well as depressive symptoms, binge drinking, and peer victimization  Scale forward and backward translated to Slovenian |
| Polk (2020) | USA | Cross-sectional | Students | 645  9^th^-11^th^ graders  M&F | School connectedness | School climate scale – student-teacher relationship subscale and Community Scale of the Relational Health Indices for Youth measure – school belongingness subscale | 0.93 | NR | NR | NR |
| Ponappa (2014) | USA | Cohort | General adolescent population | 398  10-13  M & F | Family connectedness | Social connectedness scale | NR | NR | NR | NR |
| Portwood (2005) | USA | Trial | Students | 170  4^th^-12^th^ graders  M&F | School connectedness | PSSM | 0.77 – 0.88 | NR | NR | NR |
| Poteat (2007) | USA | Cross-sectional | Students | 143  13-15 (13.5 (0.5))  M&F | School connectedness | PSSM | 0.75 at T1 and 0.66 at T2 | NR | NR | NR |
| Poulou (2019) | Greece | Development and validation | Students | 580  13-18 years  M&F | School and peer relatedness | Existence, Relatedness, and Growth Scale – Relatedness with Teachers and Peers Subscales | 0.79 and 0.78, respectively | NR | Content validity  Construct validity  Criterion validity  Predictive validity | 3 adolescents judged the items to be clear and the contents relevant  CFA confirmed a 4-factor structure with good model fit: X2(236) = 148.182, p = 0.000, X2/df = 2.15, CFI = 0.92,  TLI = 0.90, IFI = 0.95, PNFI/PGFI > 0.73, RMR = 0.056, SRMR = 0.04, RMSEA = 0.05  Positive correlations (0.19-0.56) between the scale and autonomy and competence subscales of self-determination theory  The scale and subscales was a significant predictor of adolescent wellbeing |
| Powers (2014) | USA | Trial | Students | 79  18-21  M & F | Peer, family, school, and community connectedness | Need to belong scale | NR | NR | NR | NR |
| Prado (2009) | USA | Cohort | Hispanic adolescents | 742  15.9 (1.8)  M&F | School connectedness  Family connectedness | 6 items  Parent-family connectedness scale | 0.77  0.88 | NR  NR | NR  NR | NR  NR |
| Prelow (2007) | USA | Cross-sectional | African and European American adolescents | 206  13-19  M & F | School connectedness  Ethnic identity | School connectedness scale  MEIM | 0.68 – 0.73 across samples  0.83 – 0.89 across samples | NR  NR | NR  NR | NR  NR |
| Pretty (2002) | Australia | Cross-sectional | Adolescents with and without intellectual disability | 27  13-16 (13.8)  M&F | Peer connectedness  Community connectedness | Quality of student life questionnaire (QSLQ)  Neighborhood youth inventory (NYI) | NR  NR | NR  NR | NR  NR | NR  NR |
| Pyne (2018) | USA | Cross-sectional | Early adolescents | 2158  6^th^ & 7^th^ graders  M & F | School connectedness | Social and Academic Fit scale | 0.89 | NR | NR | NR |
| Quested (2010) | UK | Cross-sectional | Dancers | 392  18.67 (2.26)  M & F | Peer relatedness | Need for Relatedness Scale | 0.87 | NR | Construct validity | χ2 (534) = 1092.12, p < 0.001; CFI = 0.95; RMSEA = 0.04; TLI = 0.94; SRMR = 0.05 |
| Raes (2020) | Belgium | Cohort | General adolescent population | 135  16-21 (17.53 (0.80))  M & F | Peer, family, school, and community connectedness | Social Connectedness Scale – Revised | 0.88 and 0.90 across time points | NR | NR | NR |
| Ramirez (2013) | USA | Trial | Traumatized school children | 20  12-17  M & F | School Connectedness | Healthy Kids Resilience Measure of School Connectedness | 0.87 | NR | NR | NR |
| Rapee (2020) | Australia | Cohort | Preadolescents | 528  11.2  M & F | School Connectedness | School Belonging Inventory | 0.64 – 0.79 | NR | NR | NR |
| Raphael (1996) | Canada | Development and validation | Students | 160  14-20 (17.4 (1.7))  M&F | Peer, family, school, and community connectedness | The Quality of Life Profile – Adolescent Version | 0.83 for the belonging subscale | NR | Convergent validity | Expected positive correlations with self-esteem, life satisfaction, social support, and life chances |
| Rasmussen (2005) | Denmark | Cross-sectional | Students | 1,537  15.8 (0.4)  M&F | School connectedness | 3 items | 0.75 | NR | NR | NR |
| Resnick (1993) | USA | Cross-sectional | Students | 36,254  7^th^-12^th^ graders  M&F | School connectedness  Family connectedness  Religious/ spiritual connectedness | School connectedness scale  Parent-family connectedness scale  Religious/ spiritual connectedness scale | NR  NR  NR | NR  NR  NR | NR  NR  NR | NR  NR  NR |
| Resnick (2004) | USA | Cohort | Youth violence perpetrators | 13,110  7^th^ -12^th^ grade  M & F | School Connectedness  Family connectedness | School connectedness scale  Parent-family connectedness scale | NR  NR | NR  NR | NR  NR | NR  NR |
| Rew (2001) | USA | Cross-sectional | Students from diverse ethnic backgrounds | 8,806  7^th^, 9^th^, & 11^th^ graders  M&F | Peer, family, school, and community connectedness | Social connectedness scale | 0.80 – 0.82 across groups | NR | NR | NR |
| Rey (2007) | USA | Cross-sectional | Urban African American adolescents | 89  2rd-6^th^ graders  M&F | School connectedness | Student report Survey of children’s social support – perceived teacher support subscale and teacher report student-teacher relationship scale | NR | NR | NR | NR |
| Reyes (2020) | USA | Cross-sectional | Filipino youth | 811  18-24 (19.7 (1.5))  M&F | Peer, family, school, and community connectedness | Social connectedness scale - revised | 0.87 | NR | NR | NR |
| Reynolds (2017) | Australia | Cross-sectional | Students | 340  13.7 (1.1)  M&F | School connectedness | 4 items | 0.90 | NR | NR | NR |
| Reynolds (2017) | USA | Cross-sectional | Black students | 729  19.94 (1.65)  M & F | Family connectedness  Ethnic-racial identity | 10-item parental acceptance subscale of the child report of parental behavior inventory  12-item MEIM | 0.96 for both parents  0.91 | NR  NR | NR  NR | NR  NR |
| Rice (2006) | USA | Cohort | High achieving students | 499  17-20 years  M&F | Peer, family, school, and community connectedness | Social connectedness scale | 0.94 and 0.95 across time points | NR | NR | NR |
| Rieckmann (2004) | USA | Cross-sectional | Navajo Adolescents | 332  14-20  M & F | Cultural connectedness | Navajo Cultural Identity Measure | 0.82 – 0.95 | NR | Construct  validity | χ2 (8, N = 332) = 43.30, p = 0.0000, NNFI = 0.92, CFI = 0.96 |
| Rieke (2017) | Australia | Cross-sectional | Students | 618  16-17 years  M&F | Peer and school connectedness | What is happening in this school (WHITS) questionnaire | 0.89 – 0.93 across sub-scales | NR | Construct validity  Convergent validity  Discriminant validity | CFA indicated that the factor loadings and constructs of the measurement used were valid and reliable  Average variance extracted > 0.5  The shared variances between the factors were lower than the square root of the average variance extracted for the individual factors |
| Riesche (2008) | USA | Cross-sectional | Students | 179  11.5 (0.8)  M&F | School connectedness | Peer network section of Substance Abuse and Mental Health Services Administration (SAMHSA) study | 0.67 | NR | NR | NR |
| Robinson (2020) | USA | Cross-sectional | Adolescents at risk of Sexually transmitted infections | 116  16-19 (17.9 (1.0))  F | Peer connectedness | 1 item | NR | NR | NR | NR |
| Roche (2012) | USA | Cross-sectional | Students | 19  13.6  M&F | School connectedness | PSSM | 0.82 | NR | Cross-cultural adaptation | Scale translated to Spanish and back-translated to English |
| Rodríguez-Meirinhos (2020) | Spain | Cross-sectional | Students | 1047  14.68 (1.53)  M & F | Peer relatedness | BPNSFS-child version | NR | NR | NR | NR |
| Roeder (2019) | USA | Cohort | Students | 334  16.2 (1.4)  19.6 (1.2)  M&F | Peer, family, school, and community connectedness | Interpersonal needs questionnaire (INQ) | 0.86 – 0.94 | NR | Construct validity | CFA on the 15-item version yielded a 2-factor structure with good model fit: RMSEA < 0.10; TLI > 0.90 |
| Rohatgi (2020) | Norway | Cross-sectional | Students | 5313  9^th^-10^th^ graders  M & F | School Connectedness | 6 items | 0.86 | NR | NR | NR |
| Rosales (2017) | USA | Cohort | Asthmatic adolescents | 245  12.0  M&F | Family Connectedness | Eco-Cultural Family Interview | 0.74 | NR | NR | NR |
| Rose (2016) | USA | Cross-sectional | Students with and without disabilities | 14,508  11-21  M & F | School Connectedness | PSSM | 0.60 – 0.64 | NR | NR | NR |
| Rose (2019) | USA | Cross-sectional | African American and Caribbean black Students | 1,170  13-17  M&F | School connectedness  Peer connectedness  Family connectedness  Religious connectedness  Community connectedness | School bonding scale from the National Comorbidity Survey  3 items  3 items  3 items from the Brief Multidimensional Measure of Religious- ness/Spirituality (BMMRS)  3 items measuring connection to neighborhood adapted from two surveys | 0.71 and 0.70, respectively  0.80 and 0.76, respectively  0.67 and 0.56, respectively  0.72 for both groups  0.83 and 0.78, respectively | NR  NR  NR  NR  NR | NR  NR  NR  NR  NR | NR  NR  NR  NR  NR |
| Rose (2010) | Australia | Cross-sectional | Students | 127  11.4 (0.6)  M&F | School Connectedness | PSSM | 0.89 | NR | NR | NR |
| Rostosky (2003) | USA | Cross-sectional | Students | 1725  13-18 (15.04 (0.69))  M & F | School Connectedness | 4 items | 0.81 | NR | NR | NR |
| Rudasill (2014) | USA | Cohort | Students from high poverty neighborhoods | 328  11-12  M&F | School connectedness | New 18-item school connectedness Scale developed with items from the national educational longitudinal study, the need satisfaction scale and the scale of caring adult relationships in school | 0.75 – 0.80 across time points for the connection to adults sub-scale  0.68 – 0.80 across time points for the school support subscale | NR | Construct validity | Factor analysis suggested a 2-factor structure with adequate model fit: χ2(76) = 139.786 -155.538 (p < 0.001); CFI = 0.895-0.928; TLI = 0.864-0.913; RMSEA = 0.057-  0.060 |
| Rutten (2015) | Belgium | Cross-sectional | Students | 472  13.0 (0.4)  M&F | School relatedness | Items adapted from multiple scales | 0.84 – 0.89 | NR | NR | NR |
| Ruttledge (2016) | Ireland | Trial | Students | 709  7-13 (10.83 (0.7))  M & F | School Connectedness | School Connectedness Scale | 0.80 | NR | NR | NR |
| Saetren (2019) | Norway | Validation | Students | 180  17.0 (1,4)  M&F | Peer, family, school, and community relatedness | Resiliency Scales for Children and Adolescents | 0.87 | NR | Cross-cultural adaptation  Construct validity | Scale translated to Norwegian and back-translated to English by independent translators  CFA of the 59-item version resulted in acceptable fit indices: CFI = 0.908, TLI = 0.904, RMSEA = 0.045. |
| Saewyc (2009) | USA and Canada | Cross-sectional | Bisexual adolescents | 70081  7^th^ -12^th^ graders  M & F | School connectedness  Family connectedness  Religious/ spiritual connectedness | 5 – 7 items across samples  7 – 11 items across samples  3 – 5 items across samples | 0.75 – 0.83  0.78 – 0.90  NR | NR  NR  NR | NR  NR  NR | NR  NR  NR |
| Saewyc (2019) | USA | Cross-sectional | Youth at a child advocacy center | 2,042  10-19 (14.4)  M&F | Family connectedness | The Child’s Report of Parental Behaviors Inventory (CRPBI) and the Parent-Child Relationship- Parent report (PCRP) | 0.74 – 0.91 | NR | NR | NR |
| Sakan (2020) | Serbia | Validation | Students | 494  12-18  M&F | Peer, family, school, and community relatedness | Basic psychological needs satisfaction and frustration scale | 0.66 – 0.88 across sub scales | NR | Cross-cultural adaptation  Construct validity  Measurement invariance  Convergent and discriminant validity | Scale culturally adapted by forward translation to Serbian, review by expert panel, and backward translation  A 6-factor model showed acceptable fit indices: χ2 (df) = 446.61 (236), AIC = 29,351.58, BIC = 29,720.86, RMSEA = 0.04, SRMR = 0.05, CFI = 0.91, TLI = 0.89  Invariance across age groups showed that the model fit the data fairly well: ΔCFI = −0.01, ΔRMSEA = −0.01  All factors measuring need satisfaction were in positive relations with the other factors measuring need satisfaction and in negative relations with the factors measuring needs frustrations |
| Sale (2005) | USA | Cross-sectional | Alcohol using Hispanic youth | 2742  9-18  M & F | Family connectedness | 9 items | 0.80 | NR | NR | NR |
| Sampasa-Kanyinga (2017) | Canada | Cross-sectional | Students | 9,912  15.2 (1.9)  M&F | School connectedness | 3 items from the perceived cohesion scale | 0.71 | NR | Construct validity | CFA revealed a single factor structure with good model fit: SRMR < 0.001,  coefﬁcient of determination = 0.79 |
| Sani (2020) | Spain | Cross-sectional | Students | 396  17-29 (19.4)  M&F | Peer, family, and community connectedness | Sense of belonging to group checklist | 0.73 – 0.84 | NR | NR | NR |
| Santana-Vega (2020) | Spain | Cross-sectional | Students | 777  11-17 (13.8 (1.1))  M&F | Community connectedness | 36-item scale designed to measure sense of belonging to the European Union | 0.89 | NR | Content validity | Expert teachers evaluated the scale for pertinence and appropriateness of items. Items were removed or modified following their feedback. |
| Santos (2016) | USA | Cross-sectional | Mexican-origin youth | 436  12.34 (0.95)  M & F | School connectedness  Ethnic identity | School connectedness scale  Private regard subscale of the Multidimensional Inventory of Black Identity–Teen | 0.76  0.87 | NR  NR | NR  NR | NR  NR |
| Santos (2016) | Portugal | Cross-sectional | Adolescents with chronic disease | 135  14 (1. 5)  M & F | Peer, family, school, and community connectedness | Scale of satisfaction with social support – Satisfaction with social support (SSS) and need for activities connected to social support (NASS) dimensions | 0.85 | NR | NR | NR |
| Sargent (2002) | USA | Cross-sectional | Youths with depressive symptoms | 443  19.8 (0.7)  M & F | School connectedness | The Sense of Belonging Instrument – Psychological Subscale (SOBI-P) | 0.86 | 0.84 | NR | NR |
| Saskia Aerts (2015) | Belgium | Cross-sectional | Sexual minority youth | 1,667  15.97(1.57)  M & F | School connectedness | PSSM | 0.88 | NR | NR | NR |
| Sass (2011) | USA & Chile | Validation | Students | 1,401  9^th^-12^th^ graders  M&F | School connectedness | HMAC – Connectedness to School, Teachers, Peers, Self-in-the-Present, and Self-in-the-Future sub-scales | 0.64 – 0.84 in the US sample  0.62 – 0.74 for the Chilean sample | NR | Construct validity  Measurement invariance  Content validity | 5-factor model showed a good model fit for the US sample (χ2 (df = 319) = 1130.73, CFI = 0.963, SRMR = 0.062, RMSEA = 0.074) and Chilean sample after correlating three residuals (χ2 (df = 316) = 1360.08, CFI = 0.933, SRMR = 0.064, RMSEA = 0.065)  Connectedness to school, teachers, and self-in-the-future factors were noninvariant across countries, whereas the factors of connectedness to peers and self-in-the-present were invariant across groups  Scale translated to a Chilean version then reviewed by researchers who suggested minor modifications and changes to language to ensure appropriateness and consistency with Chilean dialect. Finally, a Chilean school counselor also reviewed and approved each item with respect to clarity, meaning, and comprehension |
| Savci (2017) | Turkey | Cross-sectional | Students | 201  14-18  M&F | Peer, family, school, and Community connectedness | Social connectedness scale | NR | NR | NR | NR |
| Savci (2020) | Turkey | Cross-sectional | Students | 549  14-18 (15.6 (1.3))  M&F | Peer, family, school, and community connectedness | Social connectedness scale | 0.83 | NR | NR | NR |
| Sayer (2013) | UK | Validation | Students | 452  10.9 (0.6)  M&F | Community Connectedness | Sense of Community Index – Primary (SCI-P) | 0.91 for the overall scale  0.90 and 0.74 for subscales | NR | Construct validity  Content validity | A 2-factor structure that accounted for 35.84% of the total variance was the more appropriate factor solution  A pilot sample reviewed the phrasing and understandability of the questions, modifications subsequently made following their feedback |
| Sbicigo (2013) | Brazil | Cross-sectional | Students | 685  12-18 (15.1 (1.5))  M&F | School connectedness | 7 items | 0.73 | NR | NR | NR |
| Schachner (2019) | Germany | Cross-sectional | Students | 1,971  11.6 (0.7)  M&F | School Connectedness | PSSM | 0.84 | NR | NR | NR |
| Schacter (2019) | USA | Cohort | Adolescents | 119  13-18 (15.4 (0.9))  M&F | Peer and family connectedness | 3 items | Intrarater (0.69) and inter-rater reliability (0.95) | NR | NR | NR |
| Scharf (2008) | Israel | Cohort | Female students | 120  17-year olds  F | Family relatedness | Inventory of Parent and Peer Attachment and Relationships-with-Mother-Father Questionnaire | 0.97 for both parents | NR | NR | NR |
| Scherman (2008) | New Zealand | Cross-sectional | Internationally adopted children | 50  9-19 (12.9 (2.1))  M&F | Ethnic identity | MEIM | 0.83 | NR | NR | NR |
| Schiefer (2014) | USA | Cross-sectional | Adolescents | 181  13-18 (15.4 (1.3))  M&F | Ethnic identity | MEIM | NR | NR | NR | NR |
| Schreuders (2020) | Belgium Finland Germany Ireland Italy Netherlands Portugal) | Cross-sectional | General adolescent population | 10,653  15.0  M & F | School connectedness | School connectedness scale | NR | NR | NR | NR |
| Schwartz (2007) | USA | Cross-sectional | Adolescents in foster care | 18  12.2, 12.6  M&F | Ethnic identity | MEIM | NR | NR | NR | NR |
| Sebire (2016) | UK | RCT | Students | 539  10.04 (0.57)  M & F | School relatedness | Psychological need satisfaction scales | 0.84 | NR | NR | NR |
| Seil (2014) | USA | Cross-sectional | Students | 8,910  9^th^-12^th^ graders  M&F | School connectedness | 1 item | NR | NR | NR | NR |
| Selfhout (2009) | Netherlands | Cohort | Adolescents | 911  12.41  M & F | Peer relatedness | Balanced Relatedness scale | 0.88 – 0.92 | NR | Convergent validity | Correlations between commitment and balanced relatedness range from 0.32 to 0.46 (p<0.01) |
| Sellers (2006) | USA | Cohort study | African American adolescents | 314  11-17 (13.8 (1.21))  M & F | Racial identity | Multidimensional inventory of Black Identity-Teen (MIBI-T) | 0.63 – 0.73 | NR | NR | NR |
| Semanchin (2017) | USA | Validation | Out of home adolescents | 67  11.7 (1.6)  M&F | Peer Connectedness | Youth Connection Scale – Child (YCS-C) | 0.68 – 0.81 | 0.80 | Face validity  Content validity  Convergent validity | The research team and expert panel concluded that the scale had a high degree of face validity, in that the scale appeared to assess the constructs of perceived number and strengths of connections, types of overall support, and overall connectedness.  Content validity was assessed through a survey completed by the participating workers in this study that included Likert-type questions and qualitative, open-ended questions about the validity and accuracy of the child’s responses, based on the workers’ knowledge of their case and context.  Moderate correlation between the scale and Social Support Scale for Children subscales (r=0.39, p=0.001*)* |
| Serametakul (2019) | Thailand | Cross-sectional | Adolescents with asthma | 442  10-16  M & F | Peer relatedness | Basic Needs Satisfaction in Life Scale | 0.84 | NR | NR | NR |
| Shackleford (2019) | USA | Cross-sectional | Adolescents with congenital heart disease | 92  15.36 (1.66) M & F | School relatedness  Peer, family and Community relatedness | Child and Adolescent Social Support Scale  Basic Psychological Needs Scale Relatedness subscale | NR  0.76 | NR | NR | NR |
| Sharp (2019) | USA | Cohort study | Adolescents with weak family bonds | 22  14.38  M & F | Family connectedness | 8 items | 0.87 | NR | NR | NR |
| Shayo (2019) | Benin  Mozambique  Namibia  Seychelles  Tanzania | Cross-sectional | Sexually active adolescents | 15,318  13-17  M&F | Family connectedness | 1 item | NR | NR | NR | NR |
| Sheldon (2009) | Nigeria and India | Cross-sectional | Students | 1,289  14.3 (1.5)  M&F | School and family Connectedness | Learning Climate Questionnaire | 0.70 | NR | NR | NR |
| Shilo (2015) | Israel | Cross-sectional | Sexual minority adolescents | 238  16.5 (1.3)  M&F | Community connectedness | 8 items | 0.75 | NR | NR | NR |
| Shim-Pelayo (2018) | USA | Cross-sectional | Foster youth | 1354  9^th^-11^th^ graders  M&F | School connectedness | 3 items | 0.80 | NR | NR | NR |
| Shin (2019) | South Korea | Cohort | Students | 1738  5^th^ & 6^th^ graders  M & F | School relatedness | Teacher involvement, structure, and autonomy support scale | 0.78 – 0.83 | NR | NR | NR |
| Shin (2012) | South Korea | Cross-sectional | Students | 702  13-19  M&F | Peer, family  School, and community Connectedness | HMAC – Korean version | 0.82 – 0.87 | NR | NR | NR |
| Shlafer (2013) | USA | Trial | Adolescent girls at high risk for pregnancy | 253  13-17  F | Family connectedness  Peer connectedness | Parent-family connectedness scale  6 item scale adapted from the Add Health | 0.90 – 0.92  0.89 – 0.90 | NR  NR | NR  NR | NR  NR |
| Shneyderman (2013) | USA | Cohort | Students | 6,540  11-21  M & F | School connectedness | School connectedness scale | 0.72 | NR | NR | NR |
| Shochet (2006) | Australia | Cohort | Students | 2022  12-14  M & F | School connectedness | PSSM | 0.89 | NR | NR | NR |
| Shochet (2008) | Australia | Cross-sectional | Students | 153  12-18 (15.2 (1.5))  M&F | School connectedness | PSSM | 0.90 | NR | NR | NR |
| Shochet (2011) | Australia | Cohort | Students | 504  12-14 (13.3 (0.5))  M&F | School connectedness | PSSM | 0.88 – 0.92 | NR | NR | NR |
| Sieving (2000) | USA | Cohort | Students | 3,322  8-11 grade  M & F | Family connectedness | 5 items | 0.84 | NR | NR | NR |
| Simcock (2020) | Australia | Cohort | Students | 60  12.4 (1.6)  M&F | Peer, family school, and community connectedness | Social connectedness scale | NR | NR | NR | NR |
| Sladek (2015) | USA | Cohort | Students | 71  18.85 (0.54)  M & F | Peer, family, school, and community connectedness | 4 items | 0.65 – 0.81 | NR | NR | NR |
| Slap (2001) | USA | Cohort | Adolescents with a history of suicide attempts | 6,577  16.1 (1.7)  M&F | School connectedness  Family connectedness | School connectedness scale  Parent-family connectedness scale | 0.75  0.83 | NR  NR | NR  NR | NR  NR |
| Slap (2003) | Nigeria | Cross-sectional | Students | 4,218  16.3 (2.3)  M&F | School connectedness  Family connectedness | School connectedness scale  Items | 0.71  0.79 for mothers and 0.84 for mothers | NR  NR | NR  NR | NR  NR |
| Slaten (2014) | USA | Cross-sectional | Students | 436  19.28 (2.54  M & F | Peer and  family connectedness | Milwaukee Youth Belongingness Scale | 0.93 and 0.77 for family and peer connectedness, respectively | NR | NR | NR |
| Slaten (2019) | USA | Development and validation | Students | 1,773  6^th^-8^th^ graders  M&F | School, peer, and family connectedness | Milwaukee Youth Belongingness Scale | 0.78 for the overall scale | NR | Construct validity  Convergent validity  Discriminant validity | EFA suggested a 3-factor structure with excellent fit: χ2 (12, N = 906) = 10.17, p > 0.05  Significant positive correlations (0.43 – 0.70) with related measures of resilience, self-esteem, and social support  Negative correlations with bullying (-0.23) and victimization (-0.31) |
| Slee (2019) | UK | Cross-sectional | Students | 100  11.0  M&F | Peer, family, school, and community relatedness | Basic Psychological Needs Satisfaction in Life Scale – relatedness scale | NR | NR | NR | NR |
| Slomkowski (2005) | USA | Cohort | Students | 1,421  15.5 (1.7)  M&F | Family connectedness | 3 items | 0.91 | NR | Construct validity | PCA revealed a single factor solution representing a latent trait of ‘sibling social connectedness’ |
| Smerdon (2002) | USA | Cohort | Students | 11,807  8^th^&10^th^ graders  M&F | School connectedness | 9 items | 0.63 | NR | NR | NR |
| Smith (2010) | Sweden | Cross-sectional | Athletes | 206  16-19 (17.2 (1.0))  M & F | Peer relatedness | Peer Motivational Climate in Youth Sport Questionnaire (PeerMCYSQ) – Relatedness Support sub-scale | 0.81 | NR | Construct validity | CFA showed acceptable model fit for a 5-factor solution: CFI = 0.97; NNFI = 0.96; RMSEA = 0.05; SRMR = 0.07 |
| Snowshoe (2015) | Canada | Development and validation | First Nations Youth | 319  11-29 (15.3 (2.3))  M & F | Cultural connectedness | Cultural Connectedness Scale (CCS) | 0.79 – 0.87 | NR | Construct validity  Convergent validity  Content validity  Face validity | 3-factor structure that accounted for 45.48% of the variance with acceptable model fit: χ2 (103) = 247.526, p < 0.001; CFI = 0.926; TLI = 0.973; RMSEA = 0.066; WRMR = 1.069  Significant correlations of the scale with life satisfaction (0.006 – 0.176), sense of self in the present (0.131 – 0.166), sense of self in the future (0.097 – 0.276), and spiritual attendance (0.273 – 0.506)  Content validity index of 0.91  Face validity ascertained through FGDs with target youth |
| Snowshoe (2017) | Canada | Validation | First Nations Youth | 290  11-24 (14.4 (2.4))  M & F | Cultural connectedness  School connectedness  Self-connectedness | 10-item Cultural Connectedness Scale – short version (CCS-S)  HMAC-Short Version  HMAC | 0.70  0.75  0.73 and 0.74 | NR  NR  NR | Construct validity  NR  NR | -Fit indices for the CCS-S indicated good model ﬁt: χ2 (21) = 746.939, p = 0.001; CFI = 0.967; TLI = 0.970; RMSEA = 0.060; WRMR = 0.784  -Significant correlations between 0.30 – 0.43 for the subscales; p < 0.01  NR  NR |
| Snyder (2015) | USA | Cross-sectional | General adolescent population | 720  13.64 (0.11) M & F | School connectedness | School Engagement Subscale of the Drug Free Schools (DFSCA) Outcome Study questions | 0.69 | NR | NR | NR |
| Soini (2014) | Finland | Validation | Students | 4,397  14-15 years  M&F | Peer relatedness | Motivational climate in physical education scale – social relatedness subscale | 0.88 | NR | Construct validity | The 4-factor solution of the scale showed viable item loading values, ranging from 0.55 to 0.82, indicating acceptable construct validity of the items and the subscales. CFA confirmed a 4-factor structure with acceptable model fit: χ2/(df) = 6.883, AGFI = 0.97, RMSEA = 0.037, NFI = 0.97, CFI = 0.97, TLI = 0.97 |
| Sommerfeld (2019) | Israel | Cross-sectional | Youth residing in informal youth settings | 103  12-18 (15.5 (1.76))  M & F | School and family connectedness  Peer connectedness | HMAC    Interpersonal Needs Questionnaire (INQ) | 0.79 – 0.82  0.80 – 0.85 | NR  NR | NR  NR | NR  NR |
| Sonnentag (2018) | USA | Cross-sectional | Students | 168  18-24  19.36 (1.18)  M&F | Peer, family, school, and community Connectedness | Social Connectedness Scale | NR | NR | NR | NR |
| Spencer (2000) | USA | Validation | Monoracial and Multiracial Early Adolescents | 2184  12.84  M&F | Ethnic identity | MEIM | 0.85 for the entire sample, 0.79 - 0.88 across groups | NR | Construct validity  Measurement invariance | 2-factor structure most preferred on EFA, confirmed by CFA (χ2 = 590.89, CFI = 0.93)  The unconstrained (χ2 (189) = 787.40, CFI = 0.915) and constrained models showed good fit to the data (χ2 (219) = 889.55, CFI = 0.905) across three groups (white, Monoracial minority, and multiracial groups) |
| Springer (2009) | El Salvador | Validation | Students | 982  15.0 (1.4)  M&F | School connectedness | Student Perception of School Cohesion (SPSC) Scale | 0.84 for the overall scale  0.71 – 0.75 for the sub-scales | NR | Face validity  Content validity  Convergent validity  Construct validity  Cross-cultural adaptation | Education officials and principals assessed the appropriateness and relevance of items  Education officials and principals assessed the appropriateness and relevance of items. Comprehension of items was evaluated via a pilot sample of students  Expected significant inverse correlations between the subscales and measures of physical aggression and illicit drug use  EFA suggested a 3-factor structure that accounted for 61.6% of the total variance  Scale items translated to Spanish and back-translated to English by independent translators |
| Standage (2012) | England | Cohort | Students | 494  12.58 (.74)  M & F | School relatedness | Need for Relatedness Scale | 0.89 | NR | NR | NR |
| Stein (2015) | USA | Cross-sectional | Latino adolescents | 173  7^th^-10^th^ graders  M&F | School connectedness  Family connectedness | PSSM  Attitudinal familism scale | 0.80  0.83 | NR  NR | NR  NR | NR  NR |
| Steiner (2019) | USA | Cohort | Students | 14,800  15.4  M&F | School connectedness  Family connectedness | 6-item school connectedness scale  6-item parent-family connectedness scale | 0.78  0.82 | NR  NR | NR  NR | NR  NR |
| Stevens-Watkins (2010) | USA | Cohort | African American males | 1599  14–18 (16 (1.36))  M | Family connectedness | Family connectedness scale | 0.77 | NR | Construct validity | The Eigenvalue for the family connectedness factor was 2.05 and explained 68% of the total variance |
| Stewart (2015) | USA | Cross-sectional | Sexual minority youth | 475  16-20  M & F | School connectedness | School connectedness scale | NR | NR | NR | NR |
| Stjernqvist (2018) | Denmark | Trial | Students | 548  11.7 years  M&F | School connectedness | 3 items from WHO’s ‘Health Behavior in School Children’s (HBSC) survey questionnaire | 0.85 | NR | NR | NR |
| Stoddard (2011) | USA | Cross-sectional | Students | 164  6^th^-8^th^ graders  M&F | School connectedness  Family connectedness | School connectedness scale  Parent-family connectedness scale | 0.77  0.81 | NR  NR | NR  NR | NR  NR |
| Stojek (2010) | USA | Cross-sectional | Women from diverse ethnic backgrounds | 493  18.6  F | Ethnic identity | MEIM | 0.88 | NR | NR | NR |
| Stone (2015) | USA | Cross-sectional | Sexually active adolescents | 2,290  16.3 (0.1)  M&F | School connectedness  Family connectedness  Community connectedness | 1 item  1 item  1 item | NR  NR  NR | NR  NR  NR | NR  NR  NR | NR  NR  NR |
| Streb (2015) | Germany | Cross-sectional | Students | 68  8-12 years | School relatedness | Basic psychological needs scale | NR | NR | NR | NR |
| Strolin-Goltzman (2014) | USA | Cross-sectional | Elementary, middle and high school students | 793  M&F | School connectedness | Items adapted from previous studies | 0.90 – 0.94 across sub-scales | NR | Construct validity | PCA showed a 3-factor structure accounting for 72% of the variance |
| Stuart (2014) | New Zealand | Cohort | Indigenous youth | 415  10-15  M & F | Family Connectedness  Ethnic identity | Family Climate Inventory  MEIM | NR  NR | NR  NR | NR  NR | NR  NR |
| Sun (2013) | China | Cross-sectional | Students | 1627  15.47 (1.85)  M & F | School connectedness  Family Connectedness | School connectedness scale  Parenting Bonding Instrument | 0.66 - 0.75  NR | NR  NR | NR  NR | NR  NR |
| Svavarsdottir (2008) | Iceland | Cross-sectional | Chronically ill adolescents | 209  10-12 years  M&F | School connectedness | School connectedness scale | 0.79 for males and 0.81 for females | NR | Cross-cultural adaptation | -Scale translated to Icelandic by independent translators  -A reconciled version was agreed upon after consultation and discussion with specialists.  -Scale then back-translated to English.  -A conceptually relevant version was then pilot-tested and validated in a sample of Icelandic adolescents then finally proof-read by a professional |
| Tabbah (2016) | USA | Cross-sectional | Arab American adolescents | 61  12-18  M & F | Ethnic identity | MEIM | 0.63 – 0.91 | NR | NR | NR |
| Taliaferro (2017) | USA | Cross-sectional |  | 73,339  11t graders  M&F | Family connectedness  School connectedness  Peer connectedness  Community connectedness | 3 items  3 items  1 item  1item | NR  NR  NR  NR | NR  NR  NR  NR | NR  NR  NR  NR | NR  NR  NR  NR |
| Tam (2020) | USA | Cross-sectional | Students | 594  12.7 (0.7)  M&F | School connectedness  Peer connectedness | School belonging scale  Peer support scale | 0.90  0.80 | NR  NR | NR  NR | NR  NR |
| Tang (2009) | Taiwan | Cross-sectional | Students | 10,233  14.7 (1.8)  M&F | School connectedness | Adolescent Family and Social Life Questionnaire (AFSLQ) | 0.71 – 0.82 | 0.69 – 0.76 (2 weeks) | NR | NR |
| Taubman-Ben-Ari (2010) | Israel | Cross-sectional | Young drivers | 121  17-22 (18.49 (1.26))  M & F | Family relatedness | Attitudes toward Accompanied Driving Scale (ATADS) | 0.80 | NR | NR | NR |
| Taylor (2010) | UK & China | Cross-sectional | Students | 715  13-15 (14.4 (0.8))  M&F | School relatedness | Need for relatedness scale – acceptance sub-scale | NR | NR | Cross-cultural adaptation | Scale translated to Chinese, consensus reached, scale back-translated to English. Minor discrepancies resolved in meetings among the researchers |
| Taylor-Seehafor (2007) | USA | Trial | Homeless youth | 176  16-20 (18.5 (1.2))  M&F | Peer, family, school, and community connectedness | Social connectedness scale | 0.85 | NR | NR | NR |
| Thomaes (2017) | Netherlands | Cross-sectional | Adolescents | 759  12-17  M&F | Peer, family, school, and community relatedness | Basic psychological needs satisfaction scale – relatedness sub-scale | 0.87 | NR | NR | NR |
| Thomas (2004) | USA | Cross-sectional | Violent and Nonviolent American Youth | 282  7–19 (15.3)  M & F | School connectedness | 1 item | NR | NR | NR | NR |
| Thomas (2019) | USA | Cross-sectional | Homeless youth | 176  16-20 (18.5 (1.2))  M & F | Peer, family, school, and community connectedness | Social connectedness scale | 0.82 – 0.88 | NR | NR | NR |
| Thompson (2006) | USA | Cross-sectional | Students | 13,207  6^th^-10^th^ graders | School connectedness | 5 items | 0.77 | NR | NR | NR |
| Thomson (2015) | Canada | Cross-sectional | Students | 1,250  9-13 (11.7 (1.0))  M&F | School connectedness | Scale developed by Developmental studies center | 0.88 | NR | NR | NR |
| Thumann (2016) | Uganda | Cross-sectional | Students | 3,565  7-18  M & F | School connectedness | School connectedness scale | 0.62 | NR | NR | NR |
| Tian (2014) | China | Cohort | Students | 654  15.69 (1.65)  M & F | School relatedness | Basic Psychological Needs at School Scale | 0.70 – 0.86 | NR | NR | NR |
| Tian (2014) | China | Development and validation | Students | 1872  12-19 (15.50 (1.69))  M & F | School relatedness  School Connectedness | Adolescent Students’ Basic Psychological Needs at School Scale (ASBPNSS)  School Connectedness Scale | 0.77 – 0.85 across subscales  Split-half reliability of 0.61 – 0.77  0.76 | 0.71 – 0.74 across subscales (5 weeks)  NR | Construct validity  Convergent validity  Divergent validity  Predictive validity  Measurement invariance  NR | EFA derived a 3-factor model accounting for 53.81% of the total variance; confirmed by CFA: CFI = 0.98; NNFI = 0.97; RMSEA = 0.054, SRMR = 0.048  Significant high correlations with corresponding measures of autonomy, relatedness and competence  Significant weak correlations between each subscale and measures of other needs.  The correlations revealed that the Autonomy subscale positively correlated with school well-being (r = 0.43, p < 0.01), the Relatedness subscale positively correlated with school well-being (r = 0.50, p < 0.01), and the Competence subscale also positively correlated with school well-being (r = 0.45, p < 0.01). The results demonstrated that all three need subscales scores yielded significant contributions to the prediction of the school well-being measure  Configural and metric measurement invariance was established across both gender and age (ΔCFI between the configural invariance model and the metric invariance model both yielded values of 0.00; scalar invariance established only across gender (ΔCFI = 0.00)  NR |
| Tian (2015) | China | Development and validation | Students | 1228  11-19  M&F | School Connectedness | School Belonging Scale | 0.87 | NR | NR | NR |
| Tian (2016) | China | Cohort | Students | 890  11.2 (1.1)  M&F | School connectedness | School Belonging Scale | 0.83 – 0.88 across time points | NR | NR | NR |
| Tian (2016) | China | Cross-sectional | Students | 881  12-15 (13.0 (0.7))  M&F | School relatedness | Adolescent Students’ Basic Psychological  Needs at School Scale | 0.76 | NR | NR | NR |
| Tian (2016) | China | Cross-sectional | Students | 1476  15.40 (1.78)  M & F | School relatedness | Adolescent Students’ Basic Psychological Needs at School Scale (ASBPNSS) | 0.86 for the overall scale, 0.79 for relatedness subscale | NR | Construct  Validity | CFA confirmed the 3-factor structure: CFI = 0.95; TLI = 0.94; RMSEA = 0.05 |
| Tian (2019) | China | Cross-sectional | Adolescents from the general population | 4265  13.66 (2.74)  M & F | School connectedness | School Engagement Scale | 0.76 | NR | NR | NR |
| Tiller (2017) | USA | Cohort | Adolescents with cancer & comparison peers | 279  14.0 (3.0)  M&F | Peer connectedness | HMAC | 0.76 | NR | NR | NR |
| Timmons (2011) | USA | Cross-sectional | Students | 1,482  16.6 (1.2)  M&F | Family connectedness | UCLA loneliness scale | NR | NR | NR | NR |
| Tingey (2020) | USA | Trial | Adolescents | 394  13-16  M&F | School and family connectedness | HMAC | 0.72 – 0.80 across sub-scales | NR | Cross-cultural adaptation | All questions were reviewed by the local Apache study team, piloted with Apache youth, and revised as necessary prior to utilization |
| Tokic (2018) | Croatia | Cross-sectional | Students | 1,074  13.4  M&F | Family relatedness | Basic psychological needs satisfaction scale | 0.66 for mothers and 0.68 for fathers | NR | NR | NR |
| Tomek (2018) | USA | Cohort | Adolescents living in impoverished neighborhoods | 2,335  10-18  M&F | School connectedness | PSSM | 0.62 | NR | NR | NR |
| Tong (2019) | China | Cohort | Students with depression and stress | 1369  14.29 (0.57)  M & F | School connectedness | PSSM | 0.80 | NR | NR | NR |
| Tozer (2018) | Australia | Cross-sectional | Resettled youth refugees | 93  12-18 (15.46 (1.55))  M & F | School connectedness | PSSM | 0.87 | NR | NR | NR |
| Tran (2013) | Vietnam | Cross-sectional | Students | 972  12-15  M&F | School connectedness  Family connectedness | Scale adapted from the California Health Kids survey  Parental bonding instrument | 0.84  0.83 for mother and 0.84 for father | NR | NR | NR |
| Traube (2012) | USA | Cohort | Youth in child welfare system | 827  11-14 (12.7)  M&F | Family connectedness | Items from the Parent-family connectedness scale | NR | NR | NR | NR |
| Trinh (2015) | Canada | Cross-sectional | Students | 2,660  15.8 (1.3)  M&F | School connectedness | 3 items | 0.64 | NR | NR | NR |
| Tucker (2014) | USA | Cross-sectional | Students | 336  18-46 (19.7)  M&F | Peer relatedness | Basic Psychological Needs Scale  Interpersonal needs questionnaire (INQ) | 0.88  0.92 | NR | NR | NR |
| Tugba (2020) | Turkey | Cross-sectional | Adolescents with internalizing or externalizing disorders and controls | 139  13-18  M & F | Community connectedness | No mobile phone questionnaire (NMP-Q) | NR | NR | NR | NR |
| Tuicomepee (2008) | Thailand | Cross-sectional | Adolescent Tsunami survivors | 400  12-19 (15.3 (1.8))  M&F | School connectedness | School connectedness scale | 0.82 | NR | NR | NR |
| Turner (2013) | UK | Cross-sectional | Students | 48  18-23 (19.5 (1.5))  M&F | Family, school, peer, and community connectedness | 4 items | 0.83 | NR | NR | NR |
| Turner (2014) | Australia | Cohort | Students | 492  13-17  M & F | School connectedness | 4 items | 0.89 | NR | NR | NR |
| Ullman (2015) | Australia | Cross-sectional | Same-sex attracted youth | 252  14-19  M&F | School and peer connectedness | 17 items from the Feelings about yourself and school’ subsection of the state of Victoria’s (Australia) Department of Education, Employment and Training ‘Secondary School Questionnaire | 0.91 and 0.88, respectively | NR | NR | NR |
| Ullrich-French (2009) | USA | Cross-sectional | Students | 386  12.8 (0.8)  M&F | School relatedness | Need for relatedness scale | NR | NR | NR | NR |
| Ulmer (2012) | USA | Cohort | General adolescents | 12,105  Grade 7-12 | School connectedness | 5 items | 0.83 | NR | NR | NR |
| Umana-Taylor (2006) | USA | Cross-sectional | Adolescents from different ethnic backgrounds | 639  15-25 (15.5)  M&F | Ethnic identity | MEIM | 0.79 – 0.91 across groups | NR | NR | NR |
| Umana-Taylor (2013) | USA | Cohort | Adolescents of Mexican descent | 178  18.1 (0.5)  M&F | Ethnic identity | Ethnic identity scale | 0.84 - 0.89 across time points | NR | Cross-cultural adaptation | Scale translated to Spanish and back-translated to English by independent translators; final translations reviewed by a third translator and discrepancies resolved by the research team |
| Uslu (2017) | Turkey | Cross-sectional | Students | 815  11-16 (13.4 (0.8))  M&F | School connectedness | Sense of Belonging sub- scale of the Perceived Cohesion Scale (PCS) | 0.88 | NR | Cross-cultural adaptation | Scale translated to Turkish and back-translated to English and discrepancies resolved. The final version was reviewed by experts and pilot-tested in a sample of adolescents |
| Utter (2013) | New Zealand | Cross-sectional | General adolescents’ population | 9,107  14.0  M & F | Family connectedness | 9 items | 0.84 | NR | NR | NR |
| Vaca (2011) | USA | Cohort | Adolescent males | 20,745  15-19  M | School connectedness  Family connectedness | 5 items  6 items | 0.87  0.85 | NR | NR | NR |
| Van Bergen (2019) | Netherlands | Cross-sectional | Immigrant adolescents | 304  14-18  M&F | Ethnic identity | Psychological acculturation scale | 0.89 – 0.91 across groups | NR | NR | NR |
| Van Lier (2017) | New Zealand | Cross-sectional | Students | 8,500  12-18  M&F | Family connectedness  Community connectedness | 9 items  5 items | 0.84  NR | NR  NR | NR  NR | NR  NR |
| Van Renen (2008) | South Africa | Cross-sectional | Students | 87  14-16 (15.2 (0.7))  M&F | Family connectedness | 10 items | NR | NR | NR | NR |
| Van Ryzin (2019) | USA | Trial | Students | 1,890  7^th^ & 8^th^ graders  M&F | Peer relatedness | Relatedness scale | 0.71 – 0.79 across time points | NR | NR | NR |
| Van Voorhees (2009) | USA | Cohort | Adolescents | 6,504  15.7 (0.1)  M&F | Peer connectedness  Family connectedness | 7 items  7 items | NR  NR | NR  NR | NR  NR | NR  NR |
| Van Zalk (2015) | Western Europe4 | Cohort | General adolescents’ population | 2,194  13.58  M & F | Family connectedness | 5 items | 0.88 – 0.92 | NR | NR | NR |
| Vartanian 2010 | USA | Cohort | Students | 300  18.8  F | Peer, family, school, and community Connectedness | Revised social connectedness scale | 0.94 | NR | NR | NR |
| Vaughan (2011) | USA | Cross-sectional | Students | 2,593  12-17  M&F | School connectedness | 5 items | 0.77 | NR | NR | NR |
| Vaughan (2016) | USA | Cohort | Hispanic or Latino adolescent | 3,000  7^th^-12^th^ graders  M & F | School connectedness | 6 items | 0.72 | NR | NR | NR |
| Vaz (2014) | Australia | Cohort | Students with and without disabilities | 266  11.9 (0.5)  M&F | School connectedness | PSSM | NR | NR | NR | NR |
| Vera (2017) | USA | Cross-sectional | Ethnic minority, low income youth | 163  12-15  M&F | School connectedness | PSSM | 0.87 | NR | NR | NR |
| Vieno (2013) | Italy | Cross-sectional | Students | 22,552  15.7 (0.7)  M&F | School connectedness | 6-item scale from a previous study | 0.72 | NR | NR | NR |
| Vierling (2007) | USA | Cross-sectional | Students at risk for health disparities | 237  12.1 (1.2)  M&F | School and family relatedness | Acceptance subscale of the Need for Relatedness Scale | NR | NR | NR | NR |
| Vogel (2015) | USA | Cohort | General adolescent population | 7548  15.2  M & F | School connectedness  Family connectedness | 4 items  5 items | 0.80  NR | NR  NR | NR  NR | NR  NR |
| Voisin (2005) | USA | Cross-sectional | Detained adolescents | 550  14-18 (15.4 (1.0))  M&F | School connectedness | Student assessment of teacher scale | 0.85 | NR | NR | NR |
| Voisin (2011) | USA | Cross-sectional | Students | 563  13-19  M&F | School connectedness | Student assessment of teacher scale | 0.86 for males and 0.87 for females | NR | NR | NR |
| Voisin (2018) | USA | Cross-sectional | Low income | 638  15.9 (1.4)  M&F | School Connectedness | Student Assessment of Teachers Scale | 0.87 | NR | NR | NR |
| Wajda (2013) | Poland | Cross-sectional | Adolescent girls | 75  17-19  F | Family connectedness | Parental Bonding Instrument | NR | NR | NR | NR |
| Walker 2013 | USA | Cross-sectional | Students | 280  19.65 (2.8)  M&F | Ethnic identity | MEIM | 0.73 – 0.86 | NR | NR | NR |
| Wall (2007) | USA | Cohort | Maltreated Adolescents | 1,179  11-15  M&F | Family Relatedness | Rochester Assessment Package for Schools | 0.88 | NR | NR | NR |
| Wallace (2007) | USA | Cross-sectional | Black American youth | 108  13-20 (16.4)  M & F | Peer connectedness  Ethnic identity | The Peer Bond Scale  MEIM – Affirmation and Belonging Scale | 0.69  0.69 | NR  NR | NR  NR | NR  NR |
| Wallace (2012) | USA | Development and validation | Students | 962  14-20 (16.3 (1.0))  M&F | School connectedness | 32-item scale developed using items from the PSSM, the Tripod survey of student perceptions of secondary-level teaching, and Adolescent Perception of Being Known items | 0.72 – 0.88 across sub-scales | NR | Discriminant validity  Construct validity | Correlations among the latent factors ranged from 0.25 to 0.67, indicating discriminant validity across subscales  A 5-factor structure showed excellent model fit: CFI = 0.96, TLI = 0.95, RMSEA = 0.045, WRMR = 1.03 |
| Wallhead (2013) | USA | Cohort | Students | 363  15 (0.92)  M & F | School relatedness | Basic Psychological Needs Scale in Physical Education | NR | NR | NR | NR |
| Wang (2012) | USA | Cohort | Students | 1,148  7^th^-11^th^ graders  M&F | School connectedness | 5 items | 0.74 – 0.77 across grades | NR | NR | NR |
| Wang (2017) | China | Cross-sectional | Students | 255  11-16 (12.9 (1.1))  M&F | Family Relatedness | Basic Psychological need in exercise Scale | NR | NR | NR | NR |
| Waters (2010) | Australia | Cohort | Students | 5,159  12-14  M&F | School connectedness (connectedness to school)  School connectedness (connectedness to teachers)  Family connectedness | 5 items from the School connectedness scale  6 items from California Health Kids Survey  14 items | 0.81  0.83  0.74 – 0.88 | NR  NR  NR | Construct validity  Construct validity  Construct validity | One-factor model: χ2/df = 8.44; RMSEA = 0.05; AGFI = 0.99  One-factor model: x2/df = 5.62; RMSEA = 0.052; AGFI = 0.99)  Factor analysis yielded 3 factors: x2/df = 16.05; RMSEA = 0.05; AGFI = 0.99 |
| Waters (2010) | Australia | Validation | Students | 2,809  13.0  M&F | School connectedness (connectedness to school)  School connectedness (connectedness to teachers)  Family connectedness | 5 items from the School connectedness scale  6 items from California Health Kids Survey  14 items from the parent-family connectedness scale and the California Healthy Kids Survey | 0.80  0.83  0.89 overall; 0.74 – 0.88 for subscales | NR  NR | Construct  Validity  Construct validity | One factor accounting for 56% of the variance with acceptable model fit: χ2/df = 8.45; RMSEA = 0.052; AGFI = 0.993  One factor accounting for 54% of the variance with acceptable model fit: x2/df = 5.62; RMSEA = 0.052; AGFI = 0.99)  3-factor structure explaining 34% to 87% of the total variance with good model fit: RMSEA = 0.05 and AGFI = 0.99 |
| Watson (2002) | USA | Cross-sectional | Students with and without disabilities | 140  6-19 (11.1)  M & F | Peer, family, school, and community connectedness | Social belonging subscale of Quality of Student Life  Questionnaire (QSLQ) | NR | NR | NR | NR |
| Watson (2016) | USA | Cohort | Same-sex-attracted youth | 12,064  15.8 (1.7)  16.0 (1,6)  M&F | School connectedness | 3 items | 0.77 | NR | NR | NR |
| Watson (2020) | New Zealand | Cross-sectional | Multi-racial sample | 2,707  13-18  14.84 (1.37)  M & F | School relatedness | Basic Psychological Needs Satisfaction Scale | 0.72 | NR | NR | NR |
| Watson (2017) | USA | Cross-sectional | Students | 254  11.5  M&F | School connectedness | School connectedness scale | NR | NR | NR | NR |
| Watt (2009) | Australia | Trial | Adolescents | 144  18.6 (1.3)  M&F | Family, school, peer, and community Connectedness | 10 items | NR | NR | NR | NR |
| Watts (2017) | USA | Cohort | Adolescents | 9,002  16.1 (0.1)  M&F | School connectedness  Family Connectedness | School connectedness scale  Parent-family connectedness scale | 0.73  0.78 | NR  NR | NR  NR | NR  NR |
| Weber (2015) | Austria | Trial | Students | 152  13-15  13.84 (0.64)  M & F | School connectedness  Ethnic identity  Cultural connectedness | School belonging scale  MEIM  Residence Culture Identity Measure (RCIM) | 0.74  0.82  0.84 | NR  NR  NR | NR  NR  NR | NR  NR  NR |
| Weber (2018) | Austria | Trial | Immigrant students | 516  13-19 (14.9 (1.1))  M&F | School connectedness  Ethnic identity  Cultural connectedness | Academic Belonging Scale  MEIM  Residence Culture Identity Measure (RCIM) | 0.75 – 0.78 across time points  0.88 – 0.91 across time points  0.87 – 0.88 across time points | NR  NR  NR | NR  NR  NR | NR  NR  NR |
| Weisskirch (2011) | USA | Cross-sectional | Immigrant adolescents | 1222  19.78 (1.69)  M & F | Ethnic identity | Ethnic Identity Scale | 0.84 – 0.89 | NR | NR | NR |
| Wenlong Mu (2020) | China | Cross-sectional | Students | 1602  12-17  M & F | Family, school, peer, and Community connectedness | Social Connectedness Scale | 0.95 | NR | NR | NR |
| Wentzel (2018) | USA | Cohort | Students | 160  Grade8-10  M & F | School connectedness  Peer connectedness | Classroom Life Measure  Classroom Life Measure | 0.71 – 0.74  0.84 – 0.85 | NR  NR | NR  NR | NR  NR |
| White (2011) | UK | Cohort | Students | 671  14.0  M & F | Family connectedness | 2 items | 0.65 – 0.67 | NR | NR | NR |
| White (2015) | New Zealand | Cross-sectional | General population adolescents | 3017  14-15  M & F | Family, peer, and school connectedness | 8 items | 0.78 | NR | NR | NR |
| Whitehead (2018) | Scotland | Cross-sectional | Adolescents | 81  11.0 (1.9)  M&F | Family, school, peer, and community Connectedness | Resiliency Scale for Children And Adolescents | NR | NR | NR | NR |
| Whiteside-Mansell (2015) | USA | Development and validation | Students | 4,014  12.8  M&F | School Connectedness | 20-item Brief Survey of School Bonding (BSSB) | 0.59 – 0.75 across subscales | NR | Face validity  Construct validity  Convergent validity | Minor modifications were made to the scale following interviews with students.  The fit of the final four factor solution was moderate with a CFI value of 0.93 and a RMSEA value of 0.077.  Low to moderate negative correlations with measures of depression, delinquency, school misconduct, behavior consequences, school crime, and poor grades. Low to moderate positive correlations with family involvement |
| Whiting (2018) | USA | Development and validation | Students | 881  7^th^-12^th^ graders  M&F | School connectedness  School connectedness | Simple School Belonging Scale (SSBS)  PSSM | 0.91  NR | NR  NR | Convergent validity  Discriminant validity  Content validity  Construct validity  Construct validity | Moderate correlation (0.64) between the scale and a measure of social support  The belonging scale correlated weakly with the number of years the student had lived in the local community (r = 0.142, p = .007)  Scale reviewed by experts for clarity and relevance to ensure content validity  The 10-item unifactor model had good fit: χ2(35) = 150.50, p = 0.000, CFI = 0.982, TLI = 0.977, RMSEA = 0.084  A 2-factors structure on factor analysis provided the best fit to the data |
| Widome (2008) | USA | Development and validation | Young adolescents | 118  6^th^ graders  M&F | Community connectedness | Neighborhood Connection Scale | 0.86 & 0.76 for the subscales | NR | Construct validity | EFA yielded two factors with significant moderate correlation (r = 0.6, p < 0.001) |
| Wiebe (2016) | USA | Case-control | Assault victims and comparison peers | 962  10-24 (19)  M&F | Community connectedness | Neighborhood Environment scale | NR | NR | NR | NR |
| Wilkinson-Lee (2011) | USA | Cross-sectional | Students | 4,198  6^th^-12^th^ graders  M&F | School connectedness | CDC classroom climate scale | 0.88 | NR | NR | NR |
| Willard (2019) | USA | Cohort | Adolescent with brain tumors | 53  13.1 (2.3)  M&F | Peer Connectedness | HMAC | 0.72 | NR | NR | NR |
| Willgerodt (2008) | USA | Cohort | Chinese, Filipino and white adolescents | 934  13-21 (15.7 (1.4))  M&F | Family connectedness | 4 items | 0.74 – 0.77 | NR | NR | NR |
| Williams (2018) | USA | Cross-sectional | Students | 585  9^th^-12^th^ graders  M&F | School connectedness | Student engagement scale | 0.78 | NR | NR | NR |
| Williams (2012) | USA | Cohort | Sexual Minority Adolescents | 18,924  11-14  M&F | Family Connectedness | Parent connectedness scale | 0.87 | NR | NR | NR |
| Wills (2007) | USA | Trial | African American adolescents | 670  11.2 (0.4)  M&F | Ethnic identity | Multidimensional Inventory of black identity | 0.67 | NR | NR | NR |
| Willson (2004) | USA | Cohort | Students | 2,327  Middle Schoolers  M&F | School Connectedness | Safe communities Safe Schools | 0.74 | NR | NR | NR |
| Witherspoon (2009) | USA | Cohort | Urban ethnically diverse adolescent | 437  11.37(0.62)  M & F | School connectedness  Family connectedness  Community connectedness | PSSM  Network of Relationships Inventory (NRI)  10-item scale constructed from Me and My Neighborhood questionnaire & Neighborhood Cohesion Scale | 0.89  0.92 – 0.95  0.88 | NR  NR  NR | NR  NR  NR | NR  NR  NR |
| Wolford-Clevenger (2016) | USA | Cross-sectional | Students in dating relationship | 502  18.8  M&F | Family Connectedness | Interpersonal needs Questionnaire (INQ) | 0.95 | NR | NR | NR |
| Wong (2014) | China | Cross-sectional | Students | 1,917  12-15  M&F | School Connectedness | Quality of school life scale | 0.71 | NR | NR | NR |
| Wormington (2016) | USA | Cohort | Students | 9,629  15.8 (1.2)  M&F | School Connectedness | School connectedness scale | 0.87 and 0.92 across samples | NR | Construct validity | EFA suggested a one-factor structure with all item loadings > 0.40 |
| Worrell (2019) | USA | Cross-sectional | Students | 1,048  11-20 (15.7 (1.5))  M&F | School connectedness | 1 item | NR | NR | NR | NR |
| Wright (2007) | USA | Cross-sectional | Students | 87  14-18  M&F | School connectedness | Belonging scale | 0.82 | NR | NR | NR |
| Wright (2016) | USA | Cross-sectional | Boys in residential programs | 100  13-15 (13.8 (0.4))  M | School connectedness | PSSM | 0.88 | NR | NR | NR |
| Wright (2018) | USA | Cohort | Early and middle adolescents | 355  10.8 (0.7), 13.7 (0.8)  M&F | Spiritual connectedness | Personal Experience Inventory | 0.85 – 0.90 across time points | NR | NR | NR |
| Xie (2020) | China | Cross-sectional & cohort | Students | 530  13.15 (.64)  M & F | Peer relatedness | Basic Psychological Needs Scale | 0.78 | NR | NR | NR |
| Yablon (2015) | Israel | Cross-sectional | Students facing constant ethno-political conflict | 534  14-18 (16.1 (1.2))  M&F | School connectedness | California School Climate and Safety Survey | 0.74 – 0.90 across sub-scales | NR | NR | NR |
| Yan (2008) | USA | Cross-sectional | Latino youth | 322  11-13  M&F | Family connectedness  School connectedness | 4 items from the Parent-family connectedness scale  4 items | 0.88  0.85 | NR  NR | NR  NR | NR  NR |
| Yang (2020) | China | Cross-sectional | Students | 198  17-21 (18.0 (0.7))  M&F | Family, school, peer, and community relatedness | Interpersonal needs questionnaire (INQ) – thwarted belongingness subscale | 0.84 | NR | NR | NR |
| Ye (2014) | USA | Validation | Students | 890  13-19 (16.57 (1.23))  M & F | School connectedness | PSSM | NR | NR | Construct  validity | EFA and CFA identified a 3-factor structure with a separate common negative wording factor that had a good model fit: CFI = 0.97, TLI = 0.95, RMSEA = 0.060, RMR = 0.88 |
| Yen (2008) | Taiwan | Cross-sectional | Adolescents | 8004  12-18 (14.7 (1.7))  M & F | School connectedness | 4 items | 0.62 | 0.60 | NR | NR |
| Yildiz (2016) | Turkey | Cross-sectional | Students | 218  14-18 (15.8 (1.4))  M&F | Peer, family, school, and community connectedness | General belongingness scale | 0.89 | NR | NR | NR |
| Yoo (2013) | USA | Cohort | Adolescents | 335  11.2 (0.9)  M&F | Family connectedness | Social connectedness scale | 0.77 | NR | NR | NR |
| You (2008) | USA | Cross-sectional | Students | 866  5^th^-12^th^ graders  M&F | School connectedness | School connectedness scale | 0.82 | NR | NR | NR |
| You (2011) | Australia | Validation | Students | 504  13.2 (0.5)  M&F | School connectedness | PSSM | 0.70 – 0.73 across subscales | NR | Construct validity  Convergent validity | A 3-factor structure fit the data well on factor analysis: χ2 = 58.78, df = 31, p < 0.05; CFI = 0.96; NNFI = 0.97; RMSEA = 0.06  All the factor loadings were statistically significant |
| Young (2011) | Scotland | Cohort | Students | 1,698  11-19  M&F | School connectedness | Items | NR | NR | NR | NR |
| Yuen (2015) | China | Cross-sectional | Students | 543  12-17 (14.9 (0.8))  M&F | Peer, family, and school connectedness | HMAC | 0.63 – 0.76 across sub-scales | NR | NR | NR |
| Zaff (2010) | USA | Development and validation | Students | 909  8^th^-10^th^ graders  M&F | Community connectedness | Active and Engaged Citizenship Scale – Neighborhood Social Connection Subscale | 0.87 – 0.89 across time points | NR | Measurement invariance  Convergent validity  Discriminant validity  Construct validity | Longitudinal as well as gender measurement invariance established  All factor loadings as well as structural  regressions were all statistically signiﬁcant  Average variance extracted of > 050 for most of the factors  Factor analysis derived a higher-order model consisting of one second-order factor, and four first-order factors: χ2 = 4522.96, df = 460, P < 0.001, RMSEA = 0.066 |
| Zeldin (2016) | Malaysia | Cross-sectional | Students | 207  15.3  M&F | Community connectedness | School connectedness scale and 7 items measuring connectedness to peers and adults in the community | 0.79 – 0.87 across time points for the school connectedness scale and  0.75 – 0.85 for the items | NR | NR | NR |
| Zhai (2020) | China | Cross-sectional | Students | 2,758  13.5 (1.1)  M&F | School connectedness | School belonging scale | 0.85 | NR | NR | NR |
| Zhang (2018) | China | Cross-sectional | Students | 468  18-27 (19.3 (1.1))  M&F | School connectedness | PSSM | 0.85 | NR | NR | NR |
| Zhao (2015) | China | Cross-sectional | Students | 504  16-18 (16.9 (0.7))  M&F | School connectedness | PSSM | 0.82 |  | Cross-cultural adaptation  Construct validity | Scale translated to Chinese then pilot tested in a sample of adolescents which prompted minor language adjustment before use  PCA showed that each item loaded on the factor school connectedness with a factor loading above .40 |
| Zhen (2017) | China | Cohort | Students | 605  10-18 (13.4 (1.5))  M&F | School relatedness | Basic Psychological Needs Satisfaction Scale | 0.87 | NR | Construct validity | A 3-factor model showed a good model fit: χ2/df= 3.67, CFI = 0.94, TLI = 0.93, RMSEA = 0.066, SRMR= 0.051 |
| Zhu (2015) | China | Cohort | Students | 833  11-14 (12.5 (0.7))  M&F | School connectedness | Emotional Engagement subscale from the School Engagement scale | 0.79 | NR | NR | NR |
| Zilka (2020) | Israel | Cross-sectional | Immigrants and native adolescents | 559  12-18  M&F | Community connectedness | 42 items | 0.94 | NR | NR | NR |
| Zimmer-Gembeck (2009) | Australia | Cross-sectional | Students | 97  10-12  M&F | Peer, family, school, and community relatedness | 8 items | 0.14 – 0.60 | NR | NR | NR |
| Zullig (2010) | USA | Development and validation | Students | 2,049  6^th^-12^th^ graders  M&F | School connectedness | School Climate Measure | 0.77 for the connectedness subscale | NR | Content and face validity  Construct validity | Seven middle and high school students were recruited to read and review the items for clarity and readability. Through this process, items determined to be inappropriate or not salient to the target audience were deleted  8-factor structure with good model fit on factor analysis: χ2 = 1245.37 (df = 674, p < 0.0001), CFI = 0.946, TLI = 0.946, RMSEA = 0.037 |
| Yoo (2016) | South Korea | Cross-sectional | Adolescents from the general population | 4403  12.0  M & F | Peer relatedness | 2 items | NR | NR | NR | NR |
| Zhou (2012) | USA & China | Cross-sectional | Students | 273  10.54  M & F | School relatedness | Teacher as Social Context Questionnaire | 0.80 – 0.85 | NR | Construct  validity | CFI = 0.99, IFI = 0.99, TLI = 0.99, RMSEA = 0.033 |
| Zhou (2020) | China | Cohort | Adolescents from the general population | 1,009  10-15 12.97 (0.67)  M & F | School relatedness | Adolescent Students’ Basic Psychological Need | 0.76 | NR | NR | NR |
| Zimmer-Gembeck (2006) | Australia | Cross-sectional | Early Adolescents | 324  15.3 (.74)  M & F | Peer relatedness | Basic Need Satisfaction in Relationship Scale | 0.62 – 0.71 | NR | NR | NR |
| Zinn (2017) | USA | Cohort | Foster youth | 732  17-17.5  M & F | Family, peer, Community connectedness | 3 items | NR | NR | NR | NR |
| Zullig (2015) | USA | Validation | Students | 1643  9^th^-12^th^ graders  M & F | School connectedness | School Climate Measure | 0.70 – 0.92 |  | Construct validity  Measurement invariance | 10-factor structure on factor analysis: χ2 = 1242.94 (df 736, p < .01), CFI = 0.95, TLI = 0.94, RMSEA = 0.033, GFI =0 .913  The scale demonstrated adequate invariance across all three tests of invariance for both sex (SRMR = 0.040 – 0.049, CFI = 0.937 – 0.942, RMSEA = 0.0355 – 0.0366) and ethnicity (Hispanic vs Caucasian; SRMR = 0.043 – 0.062, CFI = 0.926 – 0.938, RMSEA = 0.0376 – 0.0394) |
| Zou (2014) | India | Cross-sectional | Students | 50  12-16  M | School, community, family and peer connectedness | HMAC | 0.80 | NR | NR | NR |
| Zuckermann (2020) | Canada | Cohort | Students | 26,793  9^th^-12^th^ graders  M & F | School connectedness | Items from Youth Smoking Survey | NR | NR | NR | NR |
| Zuckermann (2020) | Canada | Cross-sectional | Students | 74,501  12-18  M&F | School connectedness | 5 items | NR | NR | NR | NR |
| La Salle (2021) | 13 countries^3^ | Validation | Students | 34,923  11-17  M&F | School connectedness | Georgia School Climate Survey (GSCS) | NR | NR | Cross-cultural adaptation  Construct validity  Measurement invariance | Scale translated to target languages, reviewed by experts and back-translated to English  An 8-fator model showed a good model fit: χ2(14,748, N = 34,923) = 80850.41, p < 0.001, RMSEA = 0.042, CFI = 0.96, TLI = 0.96), SRMR = 0.05  Good configural (χ2 (7,452, N = 34,923) = 62278.8, p < 0.001; RMSEA = .054; CFI = 0.97, TLI = 0.97; SRMR = 0.05) and acceptable partial scalar invariance (χ2 (7,452, N = 34,923) = 62278.8, p < 0.001; RMSEA = 0.054; CFI = 0.97, TLI = 0.97; SRMR = 0.05) established across countries |
| Ladis (2021) | USA | Cohort | Students | 387  11.6 (0.54)  M&F | School connectedness | School Connectedness Scale | 0.75 & 0.81 across time points | NR | NR | NR |
| Laninga-Wijnen (2021) | Netherlands | Trial | Students | 1,511  10.6 (0.5)  M&F | School connectedness | Classroom Peer Context Questionnaire (CPCQ) | 0.83 | NR | NR | NR |
| Laninga-Wijnen (2021) | Netherlands | Cohort | Students | 1,206  13.61  M&F | School connectedness | Classroom Peer Context Questionnaire (CPCQ) | 0.87 | NR | Construct validity | CFA upheld the original 5-factor structure with good model fit: RMSEA = 0.06, CFI = 0.92, TLI = 0.91, SRMR = 0.07 |
| Laporte (2021) | Belgium | Cross-sectional | Students | 669  16.6 (1.2)  M&F | School relatedness | Basic Psychological Need Satisfaction and Need Frustration Scale (BPNSNF) – relatedness subscale | 0.73 – 0.83 across samples | NR | NR | NR |
| Lateef (2021) | USA | Cross-sectional | Students | 60  11-19  M&F | Ethnic identity | MEIM | NR | NR | NR | NR |
| Lau (2011) | China | Cross-sectional | Students | 1,306  11-12  M&F | School connectedness  Family connectedness | 18 items  20 items | 0.70  0.84 |  | NR  NR | NR  NR |
| Lee (2021) | South Korea | Cross-sectional | Students | 7,333  15.39 (1.70)  M&F | School connectedness | 1 item | NR | NR | NR | NR |
| Legette (2021) | USA | Cohort | Early adolescents | 322  11-13  M&F | School connectedness | 5 items | 0.83 & 0.85 across time points | NR | NR | NR |
| Lensch (2021) | USA | Cross-sectional | Students | 10,251  6^th^-12^th^ graders  M&F | School connectedness | 3 items adapted from the Youth Asset Survey | 0.55 & 0.64 across grades | NR | NR | NR |
| Lensch (2021) | USA | Cross-sectional | Juvenile offenders | 429  13-17  M&F | School connectedness | 4 items from the Youth Asset Survey | 0.79 | NR | NR | NR |
| Lensch (2021) | USA | Cohort | General adolescent population | 1,111  12-17  M&F | School connectedness | Items from the Youth Asset Survey | NR | NR | NR | NR |
| Leung (2021) | Canada | Cross-sectional | Students | 236  15.56 (1.26)  M&F | Peer and family relatedness | Multidimensional Scale of Perceived Social Support (MSPSS) | 0.92 | NR | NR | NR |
| Li (2021) | China | Cross-sectional | Students | 1,348  11-14  M&F | School relatedness | Basic Psychological Need Satisfaction Scale (BPNS) – relatedness subscale | 0.87 | NR | Construct validity | CFA produced an acceptable model fit: χ2/df (9.234), p < 0.001, RMSEA = 0.078, CFI = 0.919, TLI = 0.904), SRMR = 0.055 |
| Lin (2021) | China | Cross-sectional | Students | 1,539  15.8 (0.5)  M&F | Family connectedness  Peer connectedness | Parent-Adolescent Communication Scale (PACS)  Self-Description Questionnaire (SDQ-II) | NR  NR | NR  NR | NR  NR | NR  NR |
| Little & Garber (2000) | USA | Cross-sectional | Students | 486  11.4 (0.8)  M&F | Family, school, peer and community connectedness | Sociotropy-Achievement Scale for Children (SASC) – Connectedness subscale | 0.74 | NR | Construct validity | Factor analysis yielded a 4-factor structure with factor loadings ≥ 0.30 |
| Liu (2021) | China | Cohort | Early adolescents | 1,015  11-15  M&F | School connectedness | School connectedness scale from the California Health Kids Survey | 0.75 – 0.86 across time points | NR | NR | NR |
| Lloyd (2021) | Canada | Cohort | Students | 251,641  12-19  M&F | Community connectedness | British Columbia Adolescent Health Survey (BCAHS) | NR | NR | NR | NR |
| Logan (2011) | USA | Cross-sectional | High-risk adolescents | 2,598  12-16  M&F | School connectedness | 3-item school connectedness scale | NR | NR | NR | NR |
| Logan (2016) | USA | Cross-sectional | Youth with history of violent behavior | 3,931  9^th^-12^th^ graders  M&F | School connectedness | 3-item school connectedness scale | 0.71 | NR | NR | NR |
| Long (2021) | Scotland | Cross-sectional | Students | 2,571  15-16  M&F | School connectedness | 1 item | NR | NR | NR | NR |
| Lopez (2012) | Mexico | Cross-sectional | Athletes | 669  11-18 (13.95 (1.93))  M&F | Peer relatedness | Need for relatedness scale – Acceptance subscale | 0.87 | NR | Construct validity | CFA retained the 5 items of the subscale, which explained 65.34% of the total variance |
| Lucas-Molina (2022) | Spain | Cross-sectional | Students | 1,774  14-18  M&F | School connectedness | School engagement sub-scale of the US Department of Education 3-factor model of school climate | 0.86  Omega=0.91 | NR | NR | NR |
| Mason-Jones (2016) | South Africa | Trial | Students | 2,839  13.65 (1.01)  M&F | School connectedness | 4 items | NR | NR | NR | NR |
| Mastrotheodoros (2021) | Greece | Cohort | Immigrant adolescents | 765  12.7 (0.6)  M&F | Ethnic identity  Cultural identity | MEIM-R  MEIM-R | 0.76 – 0.87 across time points  0.64 – 0.83 across time points | NR  NR | NR  NR | NR  NR |
| Matteau-Pelletier (2020) | Canada | Cohort | Students | 6,185  7^th^-11^th^ graders  M&F | School connectedness | 6 items from the Canadian Student Tobacco, Alcohol and Drug Survey | 0.79 | NR | NR | NR |
| Maurer (2021) | Sweden | Validation | Students | 846  16-21 (18.0 (0.9))  M&F | School, family, peer, and community connectedness | EPOCH measure of adolescent wellbeing | *α* of 0.91 and ω of 0.92 for the overall scale; *α* of 0.74 and ω of 0.75 for the connectedness subscale | NR | Cross-cultural adaptation  Construct validity  Measurement invariance  Convergent validity | Scale translated to Swedish then back-translated to English by independent translators. The translations were then compared by a separate translator who found a high level of agreement in the translations  CFA supported the initial 5-factor structure with good model fit (χ2(160) = 818.72, *p < 0*.001; CFI = 0.917, TLI = 0.902, RMSEA = 0.070, SRMR = 0.044)  Scalar invariance supported across gender (χ2(350) = 1089.25, *p < 0*.001; CFI = 0.907, TLI = 0.902, RMSEA = 0.071  Expected correlations between the overall scale, including subscales and the coping self-efficacy scale (0.28 – 0.63), as well as the depression, anxiety, and stress scale (-0.22 – -0.50) |
| Mazereel (2021) | Belgium | Cross-sectional | General adolescent population | 781  17.4 (3.5)  M&F | School, family, peer, and community connectedness | 1 item | NR | NR | NR | NR |
| McBride (2020) | Canada | Cross-sectional | Youth hospitalized for psychiatric illness | 161  15.4 (1.4)  M&F | Peer connectedness | Peer subscale of the Strengths and Difficulties Questionnaire (SDQ) | NR | NR | NR | NR |
| Melendez-Torres (2021) | UK | Trial | Students | 8,179  12-15  M&F | School connectedness | Beyond Blue School Climate Questionnaire | 0.80 | NR | NR | NR |
| Molinari (2013) | Italy | Cross-sectional | Students | 614  14-18 (16.6 (1.8))  M&F | School connectedness | 5-item BPNS | NR | NR | NR | NR |
| Morales-Chicas (2017) | USA | Cohort | Latino students | 1,825  6^th^ graders  M&F | School connectedness | Gottfredson’s Effective School Battery | 0.79 | NR | NR | NR |
| Moses (2020) | USA | Cohort | Adolescents at risk for family violence | 558  12-14  M&F | Ethnic-racial identity | MEIM | 0.77 | NR | NR | NR |
| Murray-Harvey (2007) | Australia | Cross-sectional | Students | 888  10-16  M&F | School connectedness | 4 items | 0.77 | NR | NR | NR |
| Nair (2021) | USA | Cohort | Latino students | 329  13.67 (0.57)  M&F | School connectedness | School connectedness scale | 0.76 | NR | NR | NR |
| Napoli (2003) | USA | Cross-sectional | Urban adolescents | 243  11-15  M&F | School connectedness | 1 item | NR | NR | NR | NR |
| Nascimento (2021) | Brazil | Cross-sectional | Adolescents in sports | 461  10-17 (15.12 (1.44))  M&F | Peer relatedness | Basic Needs Satisfaction in Sports Scale (BNSSS) | 0.78 – 0.85 | NR | NR | NR |
| Nazari (2022) | Russia | Cross-sectional | Adolescents engaged in video games | 933  13-17  M&F | School, family, peer, and community connectedness | Social connectedness scale - Revised | 0.84 | NR | NR | NR |
| Novak (2021) | China | Cross-sectional | Students | 395  12-20 (14.0 (1.1))  M&F | School, family, peer, and community connectedness | Needs Satisfaction Scale | 0.74 | NR | NR | NR |
| Nuttman-Shwartz (2019) | Israel | Cross-sectional | Adolescents in continuous traumatic stress situations | 1,290  10-18  M&F | School connectedness | Sense of Belonging Scale | 0.88 | NR | NR | NR |
| Olcon (2017) | USA | Cross-sectional | Students | 2,560  9^th^-12^th^ graders  M&F | School and community connectedness | 6 items | NR | NR | NR | NR |
| Olowokere (2014) | Nigeria | Trial | Vulnerable children and comparison peers | 109  8-13 (12.43 (1.7))  M&F | School, family, peer, and community connectedness | New 8-item Social Connectedness Scale | 0.82 | NR | Content validity | Experts in sociology and nursing reviewed the scale for content validity |
| Omiya (2022) | Japan | Cohort | Students | 166  12-14  M&F | School connectedness | PSSM | 0.79 & 0.89 across time points | NR | Content validity | The authors and teachers reviewed the scale’s contents to ensure their validity |
| Oosterhoff (2021) | USA | Cross-sectional | Students | 213  11-15  M&F | Peer connectedness | 1 item | NR | NR | NR | NR |
| Osborne (2011) | UK | Cross-sectional | Students with Autism | 105  11-16  M&F | School connectedness | PSSM | NR | NR | NR | NR |
| Padilla-Walker (2012) | USA | Cohort | General adolescent population | 453  13-16  M&F | Family connectedness | Warmth/Support subscale od the Parenting Styles and Dimensions Questionnaire – Short Version (PSDQ) | 0.84 for each parent | NR | NR | NR |
| Patte (2021) | Canada | Cohort | Students | 101,046  9^th^-11^th^ graders  M&F | School connectedness | School connectedness scale | NR | NR | NR | NR |
| Pavarini (2022) | UK | Trial |  | 100  16-18  M&F | Peer connectedness | 1 item | NR | NR | NR | NR |
| Perkins (2021) | USA | Cross-sectional | Students | 320  6^th^-12^th^ graders  M&F | School connectedness | School connectedness scale | 0.80 | NR | NR | NR |
| Pittman (2008) | USA | Cohort | Students | 79  18-19  M&F | School connectedness | PSSM | 0.91 & 0.97 across time points | NR | NR | NR |
| Prati (2017) | Italy | Development and validation | Students | 1,800  11-20 (14.56 (2.69))  M&F | School connectedness | Scale of Sense of Community in the School (SoC-S) | *α* of 0.86 and ω of 0.92 for the overall scale; *α* of 0.80 – 0.82 and ω of 0.82 – 0.86 for the subscales | NR | Construct validity  Criterion validity  Convergent and discriminant validity | EFA suggested a 3-factor solution accounting for 67.40% of the total variance; confirmed by CFA (RMSEA = 0.013, CFI = 0.99, TLI = 0.99  The scale and its subscales significantly and moderately correlated with wellbeing  Correlations of ≥ 0.40 between the scale, including subscales and measures of class satisfactions and relationship with teachers. Additionally, there were high intercorrelations between items in the scale as well as items belonging to the same subscale |
| Quinn (2013) | UK | Cross-sectional | Students | 443  9-13 (11.83 (1.23))  M&F | Peer connectedness | New 10-item Belonging Measure adapted from previous studies | 0.86 | NR | NR | NR |
| Rageliene (2020) | Lithuania | Cross-sectional | Students | 278  10.61 (1.11)  M&F | Peer connectedness | Relational Provision Loneliness Questionnaire  (RPLQ) – Peer Group Interaction Subscale | 0.89 | NR | NR | NR |
| Raspberry (2020) | USA | Cross-sectional | Students | 1,077  12-18  M&F | School connectedness | School connectedness scale | NR | NR | NR | NR |
| Raskauskas (2010) | New Zealand | Cross-sectional | Students | 1,168  8-13 (10.6 (1.4))  M&F | School connectedness | Safe Communities-Safe Schools Survey | 0.77 & 0.83 across subscales | NR | NR | NR |
| Ratelle (2021) | Canada | Cohort | Students | 663  13.60 (0.54)  M&F | School relatedness | Need for Relatedness Scale – Intimacy Subscale | NR | NR | NR | NR |
| Raufelder (2021) | Germany | Cohort | Students | 71  14.9 (6.0)  M&F | School connectedness | 3-item School belonging scale from the Programme for International Student Assessment (PISA) | 0.78 at both time points | NR | NR | NR |
| Raymond-Flesch (2021) | USA | Cross-sectional | Latino adolescents | 599  12-15  M&F | School connectedness  Family connectedness  Community connectedness | School connectedness scale  6 items  Collective Efficacy Scale | 0.79  0.79  0.76 | NR  NR  NR | NR  NR  NR | NR  NR  NR |
| Reinboth (2004) | UK | Cross-sectional | Male adolescent athletes | 265  16.44 (1.32)  M | Peer relatedness | Need for Relatedness Scale – Acceptance Subscale | NR | NR | NR | NR |
| Rejaan (2021) | Netherlands | Cross-sectional | Students | 969  10-19 (13.4 (1.6))  M&F | Family connectedness  School connectedness  Peer connectedness  Community connectedness | 4 items  PSSM  Harter’s Perceived Competence Scale for Children  Neighborhood Youth Inventory | 0.81 – 0.90  0.81  0.75  0.80 – 0.91 | NR  NR  NR  NR | NR  NR  NR  NR | NR  NR  NR  NR |
| Ren (2021) | China | Cross-sectional | Migrant adolescents | 484  8-17 (11.65 (1.61))  M&F | School, family, peer, and community relatedness | Basic Psychological Need Satisfaction and Frustration Scale (BPNSFS) | 0.78 & 0.75 for subscales | NR | NR | NR |
| Renshaw (2015) | USA | Development and validation | Students | 1,002  6^th^-8^th^ graders  M&F | School connectedness | Student Subjective  Wellbeing Questionnaire (SSWQ) | 0.88 for the overall scale, 0.72 – 0.78 for the subscales | NR | Substantive validity  Face validity  Content validity  Structural validity  Measurement invariance  Concurrent validity | The nature and scope of the construct to be measured and the scale to be developed was conceptualized through a literature review, qualitative theme analysis, and choice of items based on face and content validity considerations  From the literature review, subconstructs that were thought to be relevant were selected for inclusion in the final scale  Research assistants working with youth critically reviewed the items for clarity and developmental appropriateness which resulted in changes in the wordings of items  EFA yielded a 4-factor structure accounting for 585 of the total variance with excellent model fit to the data (χ2 = 79.09, *df* = 62, *p* = 0.07). CFA suggested the four first-order latent factors structured as effect indicators of a single second order latent factor (i.e., student subjective wellbeing; χ2 = 236.75, *df* = 100, *p* = 0.001, CFI = 0.941, TLI = 0.920, RMSEA = 0 .052)  Configural (χ2 = 345.78, df = 200, TLI = 0.910, CFI = 0.934, RMSEA = 0.039), metric (χ2 = 366.99, df = 212, TLI = 0.910, CFI = 0.930, RMSEA = 0.039), and scalar (χ2 = 398.65, df = 228, TLI = 0.908, CFI = 0.923, RMSEA = 0.040) invariance across gender established  Expected correlations with the Student Prosociality Scale, the Academic Perseverance Scale, and the Youth Risks and Assets Survey |
| Rew (2008) | USA | Cohort | Homeless adolescents | 461  16-23 (19.52 (1.91))  M&F | School, peer, and community connectedness | School Connectedness Scale from the Minnesota Adolescent Health Survey | 0.87 | NR | NR | NR |
| Riggenbach (2021) | Switzerland  France | Cross-sectional | Adolescents with chronic pain | 120  14.52 (2.03)  M&F | School, family, peer, and community relatedness | Basic Psychological Need Satisfaction and Frustration Scale (BPNSFS) – Relatedness subscale | 0.81 | NR | NR | NR |
| Rogers (2021) | USA | Cross-sectional | Adolescent girls | 295  15.1 (1.1)  F | School connectedness | School connectedness scale | NR | NR | NR | NR |
| Ross (2021) | USA | Cross-sectional | Students | 230  10-13  M&F | Peer and school connectedness | Sense of Community in Sports Scale | 0.77 | NR | NR | NR |
| Schiffrin (2021) | USA | Cross-sectional | Students | 288  19.72 (1.77)  M&F | School, family, peer, and community relatedness | Basic Psychological Need Satisfaction and Frustration Scale (BPNSFS) – Relatedness subscale | 0.88 | NR | NR | NR |
| Schmidt (2020) | Germany | Cohort | Students | 317  9-12  M&F | School relatedness | 4 items | Omega = 0.83 – 0.87 | NR | NR | NR |
| Schweder (2021) | Germany | Cohort | Students | 754  13.56 (1.2)  M&F | School relatedness | 4 items | 0.78 | NR | NR | NR |
| Seon (2021) | USA | Cross-sectional | Students | 4,838  15.85 (0.29)  M&F | School connectedness | 6-item School belonging scale from the Programme for International Student Assessment (PISA) | 0.84 | NR | NR | NR |
| Sevil-Gulen (2021) | Turkey | Cross-sectional | Socioeconomically disadvantaged adolescents | 1,312  13-19  M&F | School Connectedness | PSSM | 0.88 | NR | Construct validity | CFA confirmed the single factor structure of the scale (χ2/df = 8.41, GFI = 0.94, SRMR = 0.05, RMSEA = 0.08 [90% CI = 0.07, 0.08], CFI = 0.92, TLI = 0.90) |
| Shah (2021) | USA | Cross-sectional | Immigrant students | 481  13.28 (0.78)  M&F | School connectedness  Family connectedness | PSSM  Family Environment Scale | 0.91  0.83 | NR  NR | NR  NR | NR  NR |
| Sharpe (2021) | Zambia &  Sierra Leone | Cross-sectional | Disabled and disadvantaged children | 468  12-15  M&F | Peer, family, and community connectedness | 3 items | NR | NR | NR | NR |
| Sidze (2015) | Kenya | Cohort | Adolescents living in informal settlements | 689  12-22, majority aged 12-19  M&F | Family connectedness | 4 items | 0.75 | NR | NR | NR |
| Simcock (2021) | Australia | Cross-sectional | Adolescents with mental health problems | 60  12.41 (1.64)  M&F | School, family, peer, and community connectedness | Social Connectedness Scale | NR | NR | NR | NR |
| Single (2020) | India | Trial | Students | 5,539  9^th^-12^th^ graders  M&F | School connectedness | Beyond Blue School Climate Questionnaire (BBSCQ) | 0.91 for the overall scale, 0.82 – 0.89 for subscales | NR | NR | NR |
| Smith (2021) | France  Italy  Lebanon  Norway  Portugal  Russia  Slovenia  Sweden  USA | Cross-sectional | Students | 35,853  Final year secondary school students  M&F | School connectedness | 9 items | 0.82 – 0.89 across countries | NR | NR | NR |
| Spiridon (2020) | UK | Cross-sectional | Students | 90  18-39 (19.5 (3.3))  M&F | School, family, peer, and community connectedness | Sense of Belonging Instrument (SOBI-P) | 0.89 | NR | NR | NR |
| Springer (2016) | Colombia | Cross-sectional | Students | 774  10-19  M&F | School connectedness | Student Perception of School Cohesion (SPSC) Scale | 0.89 for the overall scale, 0.85 & 0.81 for subscales | NR | Construct validity | PCA resulted in a 2-factor structure with eigenvalues greater than 1 |
| Standage (2006) | UK | Cross-sectional | Students | 394  11.97 (0.89)  M&F | School relatedness | Need for Relatedness Scale | 0.89 | NR | NR | NR |
| Stark (2022) | USA | Cross-sectional | Native and foreign-born adolescents | 357  15.65 (1.32)  M&F | School connectedness | PSSM | 0.89 | NR | NR | NR |
| Tobin (2017) | Canada | Cross-sectional | Adolescents in summer programmes | 253  7-14 (10.35 (2.26))  M&F | Peer relatedness | Basic Needs Questionnaire for Children (BNQ-C) – Relatedness Subscale | 0.85 | NR | NR | NR |
| Ullman (2017) | Australia | Cross-sectional | Transgender and gender-diverse students | 704  14-18  M&F | School connectedness | Attitudes Towards School Survey (ATSS) | NR | NR | NR | NR |
| Vandenkerckhove (2021) | Belgium | Cross-sectional | Students | 121  15.81 (1.50)  M&F | School, family, peer, and community relatedness | Basic Psychological Need Satisfaction and Need Frustration Scale (BPNSNFS) | 0.62 – 0.90 | NR | NR | NR |
| Vargas-Madriz (2021) | Canada | Cross-sectional | Students | 238  14.76 (1.32)  M&F | School connectedness | School Climate Questionnaire | 0.69 | NR | NR | NR |
| Wagle (2021) | USA | Cross-sectional | Students | 619  4^th^&5^th^ graders  M&F | School connectedness | PSSM | Omega = 0.82 | NR | NR | NR |
| Waterschoot (2019) | Belgium | Trial | Students | 126  9-12 (10.8)  M&F | School relatedness | 14-item People in My Life Questionnaire | 0.76 | NR | NR | NR |
| Watson (2019) | Canada | Cohort | Sexual minority adolescents | 99,373  14.8 (1.8)  M&F | Family connectedness | 3 items | NR | NR | NR | NR |
| Wilhelm (2021) | USA | Cross-sectional | Somali youth | 82,135  8^th^-11^th^ graders  M&F | Family connectedness | Parent-Family Connectedness Scale | 0.73 | NR | NR | NR |
| Yang (2021) | USA | Cross-sectional | General adolescent population | 517  14.83 (1.93)  M&F | School, family, peer, and community connectedness | Intrinsic Need Satisfaction Scale in Mobile Communication – Relatedness Subscale | 0.83 | NR | NR | NR |
| Yao (2022) | China | Cross-sectional | Students | 741  12-16  M&F | School connectedness | PSSM | 0.79 | NR | NR | NR |
| Yuen (2021) | China | Cross-sectional | Students | 8,673  10-18  M&F | Family, school, and peer connectedness | HMAC | 0.68 – 0.79 across subscales and samples | NR | Construct validity | CFA yielded a 4-factor structure: χ2 = 3198.81, df = 217, p < 0.001, CFI = 0.92, TLI = 0.90, SRMR = 0.045, RMSEA = 0.056 |
| Zeinalipour (2021) | Iran | Cross-sectional | Students | 476  16-17  M&F | School connectedness | Brown and Evans’ School Connectedness Scale | 0.83 | NR | NR | NR |
| Zhang (2021) | China | Cross-sectional | Students | 1,167  11-15  M&F | School connectedness | 1 item | NR | NR | NR | NR |
| Abidin (2021) | Indonesia | Cross-sectional | Students | 1,500  12-15  M&F | School connectedness | School Climate Measure | 0.89 | NR | Construct validity | 10-factor structure with adequate Goodness of Fit statistics (RMSEA = .051; GFI = 0.910; CFI = 0.960; NFI = 0.950; IFI = 0.960) |
| Abrinkova (2021) | Slovakia | Cohort | Students | 425  13.50 (0.67)  M&F | School connectedness | Resilience and Youth Development Module (RYDM) | 0.92 for the overall scale, 0.81 for the school connectedness subscale | NR | NR | NR |
| Adams (2021) | Canada | Cohort | Students | 1,686  18.0 (1.1)  M&F | School connectedness | College Student Wellbeing Scale – School Connectedness Subscale | NR | NR | NR | NR |
| Akindele (2021) | Nigeria | Cross-sectional | Students | 2,071  13.7 (2.1)  M&F | Family connectedness  Religious Connectedness  School Connectedness  Peer connectedness | 9 items  8 items  6 items    6 items | 0.80  0.70  0.59  0.63 | NR  NR  NR  NR | NR  NR  NR  NR | NR  NR  NR  NR |
| Caqueo-Urizar (2021) | Chile | Cross-sectional | Indigenous adolescents | 968  11-18  M&F | Ethnic identity | MEIM-R | α = 0.88; ω = 0.89 | NR | Measurement invariance  Construct validity | Strong metric (p = 0.203, ΔCFI = 0.000) and scalar (p = 0.116, ΔCFI = -0.001) invariance across ethnic groups (Aymara vs non-Aymara) established  CFA with a one-factor model showed good model fit (χ2 = 6.509, df = 2, p = 0.038, CFI = 0.999, TLI = 0.997, RMSEA = 0.068 |
| Hayixibayi (2021) | China | Cross-sectional | Students | 6,552  13.51 (2.93)  M&F | School connectedness | School connectedness scale | 0.80 | NR | NR | NR |
| Arslan (2022) | Turkey | Cross-sectional | Students | 343  10-15 (12.3 (1.3))  M&F | School connectedness scale | School Belongingness Scale | 0.72 – 0.83 | NR | Construct validity | 2 factor structure with excellent data-model fit:χ2 = 62.50, *df* = 34, *p* < 0.05, CFI = 0.97, TLI = 0.96, RMSEA (95% CI) = 0.05 (0.03, 0.06) |
| Benatov (2021) | Israel | Cross-sectional | Students | 5,688  15.58 (1.22)  M&F | School connectedness | Optimal Educational Climate Questionnaire | 0.84 & 0.88 | NR | NR | NR |
| Booker (2021) | USA | Cross-sectional | Students | 244  18.09 (0.29)  M&F | School connectedness | PSSM | Omega = 0.94 –0.96 across time points | NR | NR | NR |
| Borowsky (2013) | USA | Cross-sectional | Youth involved in bullying | 130,908  6^th^, 9^th^ & 12^th^ graders  M&F | Family connectedness | 3 items | 0.61 | NR | NR | NR |
| Brent (2021) | USA | Cohort | Adolescents seeking emergency care | 1,679  12-17  M&F | Family connectedness  School connectedness  Peer connectedness | Parent-Family Connectedness Scale  School Connectedness Scale  HMAC | 0.75  0.79  0.78 | NR  NR  NR | NR  NR  NR | NR  NR  NR |
| Abbott-Chapman (2014) | Australia | Cohort | Students | 5,472  9-15  M&F | School connectedness | School Engagement Index (SEI) | NR | NR | NR | NR |
| Cadman (2021) | UK | Cohort | Students | 944  13-14  M&F | School connectedness | School Experience Questionnaire | NR | NR | NR | NR |
| Caleon (2017) | Singapore | Trial | Students | 103  14.97  M&F | Family, school, and peer relatedness | 4 items | 0.79 & 0.88 across time points | NR | NR | NR |
| Camacho (2014) | USA | Cross-sectional | Immigrant adolescents | 468  11^th^ graders  M&F | School connectedness | 7-item scale adapted from a previous study | 0.88 & 0.90 across grades | NR | NR | NR |
| Cavioni (2021) | Italy | Cross-sectional | Students | 3,895  16.7 (1.5)  M&F | School connectedness | Student’s Sense of Community in School Scale | 0.75 | NR | NR | NR |
| Costigan (2010) | Canada | Cross-sectional | Immigrant adolescents | 95  9-15 (11.95 (1.73))  M&F | Ethnic identity | MEIM | 0.81 & 0.72 for subscales | NR | NR | NR |
| Burgueno (2020) | Spain | Validation | Students | 563  13-17  M&F | School relatedness  School relatedness | Interpersonal Behaviors Questionnaire (IBQ)  Basic Psychological Needs in Physical Education Scale | 0.78 – 0.84 across subscales  NR | 0.81 – 0.89 across subscales  NR | Cross-cultural Adaptation  Content validity  Construct validity  Measurement invariance  Criterion validity  NR | Scale translated to Spanish then back-translated to English, reviewed for equivalence and forwarded to experts for content validity analysis  Content validity index across experts ranged from 0.80 – 0.90 for representativeness, 0.85 – 0.95 for relevance, and 0.80 – 1.00 for clarity  The 24-item six-factor correlated model obtained a good fit: χ2 (237, N = 478) =  553.25, p<.001; χ2 /df = 2.33; CFI =. 093; TLI = 0.92; IFI = 0.93; SRMR = 0.047;  RMSEA = 0.053 (90% CI = 0.047, 0.059)  Measurement invariance across gender and age established  Significant positive correlations with basic need satisfaction and autonomous motivation in physical education, significant negative correlation with amotivation. Need thwarting was also significantly positively correlated with need frustration, amotivation and controlled motivation.  NR |
| Burke (2021) | Australia | Development and validation | Students | 496  11-18 (15.57 (2.43)  M&F | Family connectedness | Parent-Adolescent Relationship Scale (PARS) | H-coefficient = 0.74- 0.94 across subscale | NR | Construct validity  Discriminant validity  Measurement invariance  Concurrent and convergent validity | 3-factor model on CFA with good model fit statistics: ꭓ2 = 174.031; df = 87; *p* = 0.000; TLI = 0.97, CFI = 0.97; SRMR = 0.04, RMSEA = 0.05  Chi-square tests of independence were performed to assess for significant reductions in model fit if the largest correlation between the three factors, Connectedness and Shared Activities (*r* = .67) for the adolescent model were forced to be 1. Results demonstrated a  significant deterioration in fit; the chi-square for the adolescent model increased by 9.269 points and 1 degree of freedom to ꭓ2 = 183.300, df = 88, *p* <.001  Configural invariance: ꭓ2 (174) = 291.564, *p* < 0.001; TLI = 0.96, CFI = 0.96; SRMR = 0.05, RMSEA = 0.04; metric invariance (Δꭓ2 = (12) = 25.851, *p* < 0.011, ΔCFI = 0.004; ΔSRMR = -0.007; ΔRMSEA = -0.001) and scalar invariance (Δꭓ2 = (24) = 97.787, *p* <  0.001, ΔCFI = 0.00; ΔSRMR = -0.005; ΔRMSEA = -0.001) established across age groups (young vs older adolescents)  Significant small to large expected correlations with family environment scale, conflict behavior questionnaire, and adolescent functioning scale |
| Choi (2021) | USA | Validation | Economically Disadvantaged adolescents | 3,444  15  M&F | School connectedness  School connectedness | EPOCH measure of adolescent wellbeing  4 items from a previous study | 0.80  0.73 | NR  NR | Construct validity  Concurrent validity  Content validity  NR | CFA supported the hypothesized first-order 5-factor model (χ2 = 956.50 (df = 160), p < 0.001), RMSEA = 0.04, CFI = 0.94, TLI = 0.93) and second-order 5-factor model (χ2 = 1073.296 (df = 165), p < 0.001, RMSEA = 0.04, CFI = 0.93, TLI = 0.92)  The EPOCH scale, including the connectedness subscale, was significantly associated negatively with adolescents’ depressive symptoms (r = _0.46, _0.39, p < 0.001), anxiety (r = _0.21, _0.23, p < 0.001), behavior problems (r = _0.14, _0.13, p < 0.001), and delinquency (r = _0.11, _0.12, p < 0.001) and positively with school connectedness (r = 0.44, 0.37, p < .001) and social skills (r = 0.37, 0.36, p < .001)  The overall EPOCH measure and the  subscales of perseverance, optimism, connectedness, and happiness were congruent with theoretical expectations  NR |
| de Bruijn (2021) | Netherlands | Cross-sectional | Students in physical education | 2,224  11.8 (0.55)  M7F | School relatedness | Competence Autonomy classmate-Relatedness and teacher-Relatedness scale (CARR) | 0.86 & 0.82 | NR | NR | NR |
| Delker (2020) | Chile | Cross-sectional | Adolescents from working families | 801  14-17 (16.2 (0.2))  M&F | School connectedness | 8-item questionnaire adapted from a previous study | 0.80 | NR | NR | NR |
| Exner-Cortens (2021) | Canada | Cohort | Students | 974  12-18 (14.72 (1.75))  M&F | School, Cultural, and Spiritual connectedness | Child and Youth Resilience Measure (CYRM) | NR | NR | NR | NR |
| Beaudequin (2021) | Australia | Cross-sectional | General adolescent population | 64  12 year olds  M&F | Family, school, peer, and community connectedness | Social Connectedness Scale | NR | NR | NR | NR |
| Doty (2017) | USA | Cross-sectional | Students | 121  14.9 (1.3)  M&F | Family, school, peer, and community connectedness | 4 items | NR | NR | NR | NR |
| Ellinger (2022) | Germany | Cohort | Students | 66  9-11 (10.1)  M&F | School relatedness | Basic Psychological Needs Scale (BPNS) – relatedness subscale | 0.62 & 0.75 for classmate and teacher subscales | NR | NR | NR |
| Encina (2021) | Chile | Cross-sectional | Students | 38,286  14.8 (2.11)  M&F | School connectedness | 3 items | ω **=** 0.81 | NR | NR | NR |
| Eugene (2021) | USA | Cross-sectional | Students | 3,230  15-year-olds  M&F | School connectedness | 4 items | 0.73 | NR | NR | NR |
| Guenole (2021) | France  Belgium  Switzerland | Cross-sectional | Adolescent girls with borderline personality disorder | 104  16.6 (1.3)  F | School, family, community, peer and, self-connectedness | Depressive Experience Questionnaire (DEQ) | NR | NR | NR | NR |
| Fentahun (2014) | Ethiopia | Cross-sectional | Students | 517  18.6 (1.6)  M&F | Family connectedness | 10 items | NR | NR | NR | NR |
| Gaete (2016) | Chile | Cross-sectional | Early adolescents | 560  11.5 (12)  M&F | School connectedness | PSSM | 0.84 | NR | NR | NR |
| Gao (2022) | China | Cross-sectional | Students | 1,766  10-18 (13.33 (1.94))  M&F | School, family, peer, and community relatedness | Basic Psychological Need Satisfaction Scale (BPNS) | 0.84 | NR | NR | NR |
| Garcia-Moya (2017) | UK | Cross sectional | Students | 2,927  11-15  M&F | School connectedness | 3 items | NR | NR | NR | NR |
| Garcia-Crespo (2021) | 23 Countries^4^ | Cross-sectional | Students | 117,539  4^th^ graders  M&F | School connectedness | 5 items | NR | NR | NR | NR |
| Garcia Saiz (2021) | USA | Cohort | Gender minority adolescents | 30  13-17  M&F | Community connectedness | Gender Minority Stress and Resilience Measure | 0.81 | NR | NR | NR |
| Garwood (2020) | USA | Cross-sectional | Youth with severe emotional disturbance | 17  15.06 (1.78)  M&F | School connectedness | PSSM | 0.91 | NR | NR | NR |
| Gempp (2021) | Chile | Cross-sectional | Early adolescents | 5,619  10-12  M&F | Peer relatedness | New 5-item Peer Relatedness Scale | ρ = 0.86 | NR | Construct validity | One-factor model with factor loadings of 0.61 – 0.84 with excellent model fit (RMSEA = 0.01) |
| Guignard (2021) | France | Cross-sectional | Intellectually gifted students | 492  6^th^&10^th^ graders  M&F | Family, peer, and community connectedness | Integration Feeling Questionnaire | 0.83 – 0.89 | NR | NR | NR |
| Hatala (2021) | Canada | Cross-sectional | Students | 4,751  12-17  M&F | School, family, community, spiritual, peer, and self-connectedness | 8 items | 0.82 – 0.87 across subscales | NR | Construct validity | Factor analysis yielded a 4-factor structure with acceptable model fit statistics (χ2 =272.18; P = 0.0001; RMSEA = 0.05; AGFI = 0.97; CFI = 0.99 |
| Hautala (2021) | Finland | Cross-sectional | Students | 114,528  8^th^&9^th^ graders  M&F | School connectedness | 1 item | NR | NR | NR | NR |
| Hertz (2021) | USA | Cross-sectional | Students | 567  13-19  M&F | School connectedness  Family connectedness | School connectedness scale  5 items | 0.89  0.70 | NR  NR | NR  NR | NR  NR |
| Holzer (2021) | Austria  Germany | Cross-sectional | Students | 19,967  10-21 (14.56 (2.49))  M&F | School, family, peer, and community relatedness | 3 items adapted from the EPOCH Measure of Adolescent Wellbeing | Composite reliability of 0.83 | NR | Construct validity  Measurement invariance | CFA models revealed excellent fit  indices for all scales for both Austria, χ2 (120) = 4059.23, *p<0*.001, CFI = 0.973, TLI = 0.965, RMSEA = 0.041, SRMR = 0.032, and Germany, χ2 (120) = 244.99, *p<0*.001,  CFI = 0.975, TLI = 0.968, RMSEA = 0.041, SRMR = 0.039  Scalar invariance model had the best  Fit across countries (χ2 (60) = 725.460, *p<0*.001, CFI = 0.988, RMSEA = 0.033, BIC = 435*,* 905*.*452) |
| Huang (2018) | Taiwan | Cross-sectional | Students | 377  13-16 (13.97 (0.84))  M&F | School connectedness | School Life Characteristic Questionnaire | 0.61 | 0.90 | Content validity | The content validity of each scale ranged from0.90 to 1.00, indicating acceptable content validity. A sample of middle school students further evaluated the scale and concluded that the scale was understandable to middle school adolescents. |
| Ingoglia (2021) | Italy | Cross-sectional | Students | 190  16.47 (1.41)  M&F | Family, school, peer, and community relatedness | Basic Psychological Need Satisfaction and Frustration Scale (BPNSFS) | Composite reliability of 0.81 | NR | Construct validity | A 2-factor model showed god fit to the data : SBχ2 (19) = 17.33, *p* = 0.57, robust CFI = 1.00, RMSEA = 0 |
| Inna (2006) | USA | Cohort | Low-income students | 139  8^th^ graders  M&F | Racial-ethnic identity | 4-item Ethnic Identity-Oyserman | 0.51 – 0.88 across time points and subscales | NR | NR | NR |
| Irvin (2011) | USA | Cross-sectional | Rural youth | 6,247  9^th^-12^th^ graders  M&F | School connectedness | PSSM-Brief | 0.89 | NR | Construct validity | A final unidimensional 10-item version showed good model fit on CFA: CFI = 0.96, RMSEA = 0.07 |
| Carney (2019) | USA | Cross-sectional | Rural students | 207  3^rd^-6^th^ graders  M&F | School connectedness | The Community and Youth Collaborative Institute-School Experiences Survey (CAYCI-SES) | 0.81 | NR | NR | NR |
| Chamorro (2021) | Spain | Cross-sectional | Male football players | 478  17.43 (0.71)  M | Peer relatedness | Need for Relatedness Scale | 0.90 | NR | NR | NR |
| Katapally (2018) | Canada | Cohort | Students | 44,861  13-18  M&F | School connectedness | 6 items | NR | NR | NR | NR |
| Kumi-Kyereme (2007) | Ghana | Cross-sectional | General adolescent population | 4,430  12-19  M&F | Family, community, school, peer, and religious connectedness | 5 items | NR | NR | Cross-cultural adaptation | Items translated to target local languages and back-translated to English |
| Kipp (2020) | USA | Cross-sectional | Youth soccer players | 387  8-13 (10.7 (1.2))  M&F | Peer relatedness | Basic Psychological Needs Scale (BPNS) | 0.74 – 0.85 | NR | NR | NR |
| Hawkins (2021) | USA | Cohort | Students | 3,265  14-19  M&F | School connectedness | 4 items from the panel study of income dynamics | 0.73 | NR | NR | NR |
| Huhtiniemi (2019) | Finland | Cross-sectional | Students | 502  11.86 (0.24)  M&F | Peer relatedness | Basic Psychological Needs in Physical Education Scale | 0.74 & 0.81 across grades | NR | Cross-cultural adaptation  Construct validity | Scale forward translated to Finnish and back-translated to English, then reviewed and harmonized by experts  A 3-factor structure fitted the data well in both grades |
| Ammar (2021) | Egypt | Development and validation |  | 774  13-19  M&F | School, family, religious, national, and cultural connectedness | Sense of Belonging Scale for Adolescents (SOBS) | Cronbach's alpha and McDonald's  omega for the entire scale were both 0.916,  composite reliability (CR) for the entire scale was  0.922; alpha of 0.69 – 0.90, omega of 0.72 – 0.91 and CR of 0.75 – 0.91 for subscales | 0.70 – 0.90 across subscales | Content validity  Construct validity  Convergent and divergent validity | An expert panel established content validity (overall content validity index of 0.96)  EFA established a 5-factor structure accounting for 43.06% of the total variance. Confirmed by CFA (x2=800.741, CFI=0.81, RMSEA (95% CI) = 0.090 (0.084-0.097)  SOBS was positively correlated with the Psychological Wellbeing, positive affect, and self-esteem while it was negatively correlated with negative affect and depression |
| 1 – Argentina, Albania, Australia, Austria, Belgium, Brazil, Bulgaria, Chile, Czech Republic, Denmark, Finland, France, Germany, Greece, Hong Kong-China, Hungary, Iceland, Indonesia, Ireland, Israel, Italy, Korea, Latvia, Liechtenstein, Luxembourg, FYR Macedonia, Mexico, Netherlands, New Zealand, Norway, Peru, Poland, Portugal, Romania, Russian Federation, Spain, Sweden, Switzerland, Thailand, the United Kingdom, and the United States  2 – Albania, Austria, Belgium, Bulgaria, Croatia, Cyprus, Czech Republic, Faroe Islands, France, Macedonia, Georgia, Germany, Greece, Hungary, Iceland, Ireland, Italy, Latvia, Liechtenstein, Lithuania, Malta, Moldova, Montenegro, Netherlands, Poland, Romania, Slovenia and Ukraine  3 – Belgium, Germany, Greece, Hungary, Italy, Jamaica, Latvia, Lithuania, Malta, Puerto Rico, Russia, Slovakia, and the United States  4 – Austria, Belgium, Bulgaria, Czech Republic, Denmark, England, Finland, France, Germany, Hungary, Ireland, Italy, Latvia, Lithuania, Malta, Netherlands, Northern Ireland, Poland, Portugal, Slovak Republic, Slovenia, Spain, Sweden  CFA-Confirmatory factor analysis; EFA -Exploratory factor analysis; PCA-Principal component analysis, PSSM-Psychological Sense of School Membership Scale; MEIM-Multigroup Ethnic Identity Measure; MEIM-R-Multigroup Ethnic Identity Measure-Revised; FACES-II-Family Adaptability and Cohesion Evaluation Scale-II; HMAC-Hemingway’s Measure of Adolescent Connectedness; EPOCH-Engagement, Perseverance, Optimism, Connectedness, and Happiness Measure of Adolescent Wellbeing; CFI-Comparative fit index; DIF-Differential item functioning; GFI-Goodness of fit index; AGFI-Adjusted goodness of fit index; AIC-Akaike information criterion; χ2-Chi-square; df-Degree of freedom; RMSEA-Root mean square of approximation; SRMR-Standardized root mean square residual; TLI-Tucker-Lewis Index; a- Cronbach’s alpha; ICC-Intraclass correlation; r-Pearson correlation coefficient; KMO-Kaiser-Meyer-Olkin Measure; FGDs-Focused group discussions; NR-Not Reported; USA-United States of America; UK-United Kingdom | | | | | | | | | | |
