# Supplementary material for: Adolescent Connectedness: A Scoping Review of Available Measures and Their Psychometric Properties
Source: Front Psychol. 2022 May 18;13:856621. doi: 10.3389/fpsyg.2022.856621 (PMC9159472; doi:10.3389/fpsyg.2022.856621)
Supplement: Supplementary file 1 [file Data_Sheet_1.zip › Supplementary File 3_Total Measures.docx]

**Supplementary File 3: Measures of Adolescent Connectedness**

| School Connectedness Scale |
| --- |
| Georgia School Climate Survey |
| Classroom Peer Context Questionnaire (CPCQ) |
| Parent-Adolescent Communication Scale (PACS) |
| Self-Description Questionnaire (SDQ-II) |
| Sociotropy-Achievement Scale for Children (SASC) – Connectedness Subscale |
| British Columbia Adolescent Health Survey (BCAHS) |
| Peer Subscale of the Strengths and Difficulties Questionnaire (SDQ) |
| Needs Satisfaction Scale |
| Warmth/Support Subscale of the Parenting Styles and Dimensions Questionnaire – Short Version (PSDQ) |
| Scale of Sense of Community in the School (SoC-S) |
| New 10-item Belonging Measure |
| Harter’s Perceived Competence Scale for Children |
| School Connectedness Scale from the Minnesota Adolescent Health Survey |
| Sense of Community in Sports Scale |
| Family Environment Scale |
| Basic Needs Questionnaire for Children (BNQ-C) – Relatedness Subscale |
| School Climate Questionnaire |
| People in My Life Questionnaire |
| Intrinsic Need Satisfaction Scale in Mobile Communication – Relatedness Subscale |
| Brown and Evans’ School Connectedness Scale |
| Resilience and Youth Development Module (RYDM) |
| College Student Wellbeing Scale – School Connectedness Subscale |
| Optimal Educational Climate Questionnaire |
| School Engagement Index (SEI) |
| School Experience Questionnaire |
| Student’s Sense of Community in School Scale |
| Interpersonal Behaviors Questionnaire (IBQ) |
| Parent-Adolescent Relationship Scale (PARS) |
| Competence Autonomy Classmate-Relatedness and Teacher-Relatedness Scale (CARR) |
| Child and Youth Resilience Measure (CYRM) |
| Depressive Experiences Questionnaire (DEQ) |
| Gender Minority Stress and Resilience Measure |
| New 5-item Peer Relatedness Scale |
| Integration Feeling Questionnaire |
| School Life Characteristic Questionnaire |
| 4-item Ethnic Identity-Oyserman |
| School Connection Measure |
| Sense of Belonging Scale for Adolescents (SOBS) |
| Bao’s School Connectedness Scale |
| Chicago Public Schools Student Connection Survey |
| Teacher Academic Support in the Classroom Life Measure |
| 51-item Parker’s School Connectedness Scale |
| Simple School Belonging Scale (SSBS) |
| Basic Psychological Needs Satisfaction and Frustration Scale |
| Health Behavior in School Children’s (HBSC) Survey |
| UCLA Loneliness Scale |
| Social Connectedness Scale |
| Hemingway’s Measure of Adolescent Connectedness (HMAC) |
| What is Happening in This School (WHITS) Questionnaire |
| Quality of Students Life Questionnaire (QSLQ) |
| Interpersonal Needs Questionnaire (INQ) |
| The Engagement, Perseverance, Optimism, Connectedness, and Happiness (EPOCH) Measure of Adolescents Wellbeing |
| Contextualized Assessment Tool for Risk and Protection Management (CATPRM) |
| Middle Years Development Instrument (MDI) |
| Milwaukee Youth Belongingness Scale (MYBS) |
| The Sense of Belonging Instrument – Psychological (SOBI-P) |
| Relatedness Questionnaire |
| The Parental Bonding Instrument |
| Relatedness Scale |
| SAMHSA Government Performance and Results Act (GPRA) Participant Outcome Measures for Discretionary Programs |
| Basic Psychological Needs in Exercise Scale |
| The High School Questionnaire (HSQ) |
| Need for Relatedness Scale |
| Motivational Climate in Physical Education Scale (MCPES) |
| Peer Motivational Climate in Youth Sport Questionnaire (PeerMCYSQ) |
| The Resiliency Scales for Children and Adolescents (RSCA) |
| Youth Asset Survey |
| School Belonging Scale from the Programme for International Student Assessment (PISA) |
| Multigenerational Interconnectedness Scale (MIS) |
| Sense of Belonging to School Scale (SEBES) |
| School Belongingness Scale |
| Student Subjective Wellbeing Questionnaire (SSWQ) |
| Gottfredson’s Effective School Battery (ESB) |
| Need to Belong Scale |
| Psychological Sense of School Membership Scale (PSSM) |
| Multigroup Ethnic Identity Measure (MEIM) |
| Scale measuring Sense of Belonging to the European Union |
| Sense of Community Scale for Adolescents (SOC-A) |
| Brief Scale of Sense of Community in Adolescents |
| Anderson-Butcher & Conroy’s 5-item Belonging Scale |
| Neighborhood Youth Inventory |
| Perceived School Experiences Scale (PSES) |
| Neighborhood Cohesion Instrument |
| Family Attachment Scale |
| Multidimensional Scale of Perceived Social Support |
| Family Adaptation and Cohesion Evaluation Scale (FACES II) |
| The Differentiation in the Family System Scale |
| Relationship-with-Mother-Father Questionnaire |
| Parent Relatedness Scale |
| Family Connectedness Scale |
| Social Identity Scale |
| Chestnut Lodge Adolescent Interaction Scale |
| The Child Loneliness Scale |
| Social Isolation Questionnaire for Adolescents (CSIQ-A) |
| Add Health Measure of Peer Connectedness |
| Add Health Measure of Community Connectedness |
| Adolescent Personality Style Inventory |
| New 8-item School Connectedness Scale |
| Dimensions of Identity Development Scale |
| Fisher’s Spiritual Wellbeing Scale |
| Health Behavior Questionnaire |
| Exeter Identity Transition Scale (EXITS) |
| Family Affluence Scale |
| Self in a Social Context – Social Connectedness Scale |
| US Department of Education 3-Factor Model of School Climate |
| Beyond Blue School Climate Questionnaire (BBSCQ) |
| Teacher and Classmate Support Scale |
| Student-Teacher Relationship Scale |
| Inventory of School Climate-Student Scale |
| The Inventory of Teacher-Student Relatedness |
| Pre-Adolescent Civil Engagement Scale (PACES) |
| General Belongingness Scale |
| Community Connectedness Scale |
| Psychological Sense of Community |
| Racial Identity Attitude Scale |
| Interpersonal Support Evaluation List (ISEL) |
| Social Context Virtual Subscale (SSC-VC) |
| The Mannheim Individuation Questionnaire |
| Inventory of Parent and Peer Attachment |
| Balanced Relatedness Scale |
| Subjective Family Picture |
| Family Implicit Rules Profile (FIRP) |
| Scale from the Minnesota Adolescent Health Survey and Youth Risk Behavior Survey |
| Classmate Social Isolation Questionnaire (CISQ-A) |
| The Loneliness and Social Dissatisfaction Questionnaire |
| New 7-item Peer Connectedness Scale |
| Youth Connections Scale |
| Psychological Needs Satisfaction in Education Scale |
| General Connectedness Scale for Children |
| The Adolescent Resilience Questionnaire |
| Awareness of Connectedness Scale (ACS) |
| Interview Schedule for Social Interaction |
| Children’s Intrinsic Needs Satisfaction Scale |
| Scale of Satisfaction with Life Support (SSSS) – Satisfaction with Social Support (SSS) and Need for Activities Connected to Social Support (NASS) Dimensions |
| Marjoribank’s School Capital Scale |
| Community and Youth Collaborative Institute School Experience Survey |
| California Health Kids Survey |
| The School Connection Scale |
| Quality of School Life Scale |
| Social Questionnaire for Secondary Students (SQSS) |
| Child- Adolescent Social Support Scale |
| School Sense of Belonging |
| Sense of Belonging Questionnaire |
| The Brief Implicit Association Test |
| Social Bonding Scale |
| Social Comparison Scale |
| Cultural Connectivity Scale – California |
| Inclusion of Community in the Self Scale |
| Family Assessment Measure–3rd edition (FAM-III) |
| National Survey of Child Health |
| Family Relatedness Scale |
| Parent-Family Connectedness Scale |
| Positive Youth Development Measure |
| Sense of Community Membership |
| Family Assessment Clinician Interview (FACI) |
| Parenting Style Index |
| 36 items of the Egna Minnen Betraffande Uppfostran for Adolescents (EMBU- A) |
| Sense of Community Scale |
| School Adaptation Scale |
| Autonomy–Connectedness Scale |
| The Peer Involvement in Delinquent Acts Scale (PIDAS) |
| Youth Risk Behavior Survey |
| Group Belonging Scale |
| Peer Scale of the Self-Description Questionnaire |
| Millon Adolescent Clinical Inventory |
| Interpersonal Relationship Quality Scale |
| Motivation to Remain Friends Questionnaire |
| Social Bond Measure |
| Resilience Scale |
| Vaux Social Support Record |
| Australian Community Participation Questionnaire |
| Social Relatedness Scale |
| Developmental Assets Profile |
| New 20-item School Belonging Scale |
| The Community and Youth Collaborative Institute-School Experiences Survey (CAYCI-SES) |
| School Bonding Scale |
| School as Caring Community Profile II |
| Youth Risk Behavior Survey |
| Classroom Belonging and Support Scale |
| The Spanish version of the US-based Maryland Safe and Supportive Schools School Climate Survey |
| Social Support Rate Scale |
| Personal Wellbeing Index |
| Inclusion of Others in Self |
| Family Sense of Belonging Scale |
| Perceived Emotional / Personal Support Scale |
| Teen Supplemental Survey – Connectedness with Caregivers Subsection |
| The Modiﬁed Friendship Scale (MFQS) |
| The Family Belonging Scale-Revised (FBS-R) |
| Child Report of Parental Behavior Inventory |
| Relational Provision Loneliness Questionnaire (RPLQ) |
| Measure of Peer Affiliation |
| Balanced Relatedness Scale |
| Religious Collective Self-Esteem Scale (RCSES) |
| Depressive Experiences Questionnaire for Adolescents (DEQ-A) |
| Classroom Sense of Community Scale (SoC-C) |
| Social Safeness and Pleasure Scale |
| Existence, Relatedness and Growth Scale |
| The Quality of Life Profile – Adolescent Version |
| The Child Rating Scale (CRS) |
| The Dormitory Belonging Scale |
| Sense of Belonging Scale |
| Collective Efficacy Scale (CES) |
| Basic Need Satisfaction at Work Scale |
| Social Disconnection Scale |
| Sense of Community Index |
| Sense of Community Index-Primary (SCI-P) |
| Acculturation, Habits and Interests Multicultural Scale for Adolescents (AHIMSA) |
| Hawaiian Culture Scale |
| Neighbors and Sense of Community Subscales |
| Youth Risk Behavior Survey |
| Youth Relationship with Parent Index |
| Father-Son Closeness and Connectedness Scale |
| Family Climate Inventory |
| Attitudes toward Accompanied Driving Scale (ATADS) |
| Peer Support Scale |
| Sense of Belonging to Group Checklist |
| State of Victoria’s (Australia) Department of Education, Employment and Training Secondary School Questionnaire |
| Social and Academic Fit Scale |
| School Climate Measure |
| School Belonging Scale |
| School Belongingness Scale |
| Sense of Belonging at School Measure |
| School Attachment Scale |
| School Engagement Scale |
| Health Pathway Child Report |
| Student Teacher Connectedness Measure |
| Religious/Spiritual Connectedness Scale |
| Brief Multidimensional Measure of Religiousness/Spirituality |
| 15-item Spiritual Connectedness Scale |
| Measure of Attitudes Toward Social Networking Sites (MATS) |
| State Social Disconnection Scale |
| Ethnic Identity Scale |
| Ethnic and Moral Identity Scale |
| Neighborhood Intergenerational Closure scale |
| Psychological Acculturation Scale |
| Neighborhood Environment Scale |
| School Belonging Measure |
| Rochester Assessment Package for Schools (RAPS) |
| Network of Relationships Inventory (NRI) |
| Family Resilience Assessment Scale |
| Adolescent Promoting and Relatedness Scale |
| Parental Behavior Measure |
| Parent-Child Relationship Quality Scale |
| Subjective Family Image Test |
| Voice of Connecticut Youth Survey |
| Multitrait-Multimethod Model |
| Loneliness and Social Dissatisfaction Questionnaire for Young Children |
| Social Questionnaire for Secondary students |
| The Peer Bond Scale |
| Classroom Life Measure |
| Youth Connection Scale – Child (YCS-C) |
| Social Capital Questionnaire for Adolescent Students |
| Community and Youth Collaborative Institute School Experience Scale |
| Perceived Cohesiveness scale |
| Needs Satisfaction in the Workplace Questionnaire |
| Sense of School Belonging Scale |
| Loneliness Scale |
| School Wellbeing Profile |
| Child and Adolescent Social Support Scale |
| Connected Classroom Climate Inventory |
| Personal Experience Inventory |
| Sense of Belonging Instrument |
| Active and Engaged Citizenship Scale |
| Alaska Native Cultural Identification (ANCI) |
| Navajo Cultural Identity Measure (NCIM) |
| Multidimensional Inventory of Black Identity–Teen (MIBI-T) |
| Cultural Connectedness Scale |
| No Mobile Phone Questionnaire (NMP-Q) |
| Hofer Connectedness Scale |
| Relationship Problem Inventory |
| Parent-Child Relationship Scale |
| Attitudinal Familism Scale |
| Autonomy and Relatedness Coding System |
| Eco-Cultural Family Interview |
| Tyler’s Work on Institutional Engagement |
| School Adaptation Scale |
| School Connectedness Questionnaire |
| Attitudes Toward School Survey (ATSS) |
| Scale of Caring Adult Relationships in School |
| The Community and Youth Collaborative School Experience Scale (CAYCI-SES) |
| Basic Psychological Needs in Physical Education Scale |
| School Climate Survey |
| ELS:2002 Base Year Student Questionnaire |
| Basic Needs Satisfaction in Sport Scale (BNSSS) |
| Neighborhood Connection Scale |
| Me and My Neighborhood Questionnaire |
| Neighborhood Cohesion Scale |
| Teacher Bonding Scale |
| Social and Academic Fit scale |
| Healthy Kids Resilience Measure of School Connectedness |
| School Climate and Function Scale |
| School Climate and School Identification Measure – Student (SCAISM-st) |
| School Climate Scale |
| Community Scale of the Relational Health Indices for Youth Measure |
| School Belonging Inventory |
| Social Questionnaire for Secondary students |
| School Liking and Avoidance Questionnaire |
| Survey of Children’s Social Support |
| Class Connectedness Scale |
| New 18-item School Connectedness Scale |
| New 15-item School Connectedness Scale |
| Teacher Involvement, Structure, and Autonomy Support Scale |
| Social Competence Rating Scale for Children (SCRSC) |
| Learning Climate Questionnaire |
| Perceived Cohesion Scale |
| School Engagement Subscale of the Drug Free Schools (DFSCA) Outcome Study Questions |
| Academic Belonging Scale |
| Student Perception of School Cohesion (SPSC) Scale |
| Student Assessment of Teachers Scale |
| Classroom Life Measure |
| Jenkins School Bonding (JSB) |
| Brief survey of School Bonding (BSSB) |
| Adolescent Family and Social Life Questionnaire (AFSLQ) |
| Teacher as Social Context Questionnaire |
| Safe Communities-Safe Schools Survey |
| Scale Developed by Developmental Studies Center |
| Adolescent Student’s Basic Psychological Needs at School Scale |
| Student Assessment of Teacher Scale |
| CDC Classroom Climate Scale |
| Student Engagement Scale |
| Belonging Scale |
| California School Climate and Safety Survey |
| New 3-item Teacher-Students Relationship Scale |
| Peer Relationships Scale |
| School Bonds Scale |
| Residence Culture Identity Measure (RCIM) |
| New 3-item Peer Relationships Scale |
| New 4-item Family Connectedness Scale |
| New 10-item Family Connectedness Scale |
| New 11-item Family Connectedness Scale |
| School Bonding Scale from the National Comorbidity Survey |
| Multi-Religion Identity Measure (MRIM) |
| New 6-item Scale measuring Religious Identity |
| 6-item Cultural Connectedness Measure adapted from the MEIM |
