# Supplementary material for: Adolescent Connectedness: A Scoping Review of Available Measures and Their Psychometric Properties
Source: Front Psychol. 2022 May 18;13:856621. doi: 10.3389/fpsyg.2022.856621 (PMC9159472; doi:10.3389/fpsyg.2022.856621)
Supplement: Supplementary file 1 [file Data_Sheet_1.zip › Supplementary File 4-Measures per domain of Connectedness.docx]

**Supplementary File 4a: Measures of adolescent school connectedness and their frequency of use**

| **Measure** | **Frequency of use** |
| --- | --- |
| Psychological Sense of School Membership Scale (PSSM) | 109 |
| School Connectedness Scale | 84 |
| Basic Psychological Needs Satisfaction Scale (BPNS) | 21 |
| School Belonging Scale | 8 |
| School Belongingness Scale | 7 |
| Need for Relatedness Scale | 7 |
| School Belonging Scale from the Programme for International Student Assessment (PISA) | 5 |
| Hemingway’s Measure of Adolescent Connectedness (HMAC) | 5 |
| School Engagement Scale | 5 |
| School Climate Measure | 5 |
| California Health Kids Survey | 4 |
| The Community and Youth Collaborative Institute-School Experiences Survey (CAYCI-SES) | 4 |
| Gottfredson’s Effective School Battery | 4 |
| Student Assessment of Teacher Scale | 3 |
| Beyond Blue School Climate Questionnaire (BBSCQ) | 3 |
| Perceived Cohesion Scale | 2 |
| Student-Teacher Relationship Scale | 2 |
| Health Behavior in School Children’s (HBSC) Survey Questionnaire | 2 |
| School Bonding Scale | 2 |
| School Attachment Scale | 2 |
| The High School Questionnaire (HSQ) | 2 |
| Quality of School Life Scale | 2 |
| Student Subjective Wellbeing Questionnaire (SSWQ) | 2 |
| Need to Belong Scale | 2 |
| What’s Happening In This School (WHITS) Questionnaire | 2 |
| School as Caring Community Profile II | 2 |
| Classroom Belonging and Support Scale | 2 |
| Social and Academic Fit Scale | 2 |
| Anderson-Butcher & Conroy’s 5-item Belonging Scale | 2 |
| Classroom Peer Context Questionnaire (CPCQ) | 2 |
| Bao’s School Connectdness Scale | 2 |
| US Department of Education 3-factor model of school climate | 2 |
| Sense of Belonging Scale | 2 |
| The Engagement, Perseverance, Optimism, Connectedness, and Happiness (EPOCH) Measure of Adolescent Wellbeing | 2 |
| Basic Psychological Needs in Physical Education Scale | 2 |
| Student Perception of School Cohesion (SPSC) Scale | 2 |
| Safe Communities-Safe Schools Survey | 2 |
| Youth Asset Survey | 2 |
| Perceived School Experiences Scale (PSES) | 2 |
| Georgia School Climate Survey | 1 |
| Scale of Sense of Community in the School (SoC-S) | 1 |
| School Climate Questionnaire | 1 |
| People in My Life Questionnaire | 1 |
| Brown and Evans’ School Connectedness Scale | 1 |
| Resilience and Youth Development Module (RYDM) | 1 |
| College Student Wellbeing Scale – School Connectedness Subscale | 1 |
| Optimal Educational Climate Questionnaire | 1 |
| School Engagement Index (SEI) | 1 |
| School Experience Questionnaire | 1 |
| Student’s Sense of Community in School Scale | 1 |
| Competence Autonomy Classmate-Relatedness and Teacher-Relatedness Scale (CARR) | 1 |
| School Connection Measure | 1 |
| Simple School Belonging Scale (SSBS) | 1 |
| Chicago Public Schools Student Connection Survey | 1 |
| Teacher Academic Support in the Classroom Life Measure | 1 |
| School Connectedness Questionnaire | 1 |
| School Sense of Belonging | 1 |
| Teacher and Classmate Support Scale | 1 |
| Inventory of School Climate-Student Scale | 1 |
| The Inventory of Teacher-Student Relatedness | 1 |
| New 3-item Teacher-Students Relationship Scale | 1 |
| Pre-adolescent Civil Engagement scale (PACES) | 1 |
| Marjoribanks School Capital Scale | 1 |
| 51-item Parker’s School Connectedness Scale | 1 |
| Community and Youth Collaborative Institute school Experience Survey | 1 |
| The School Connection Scale | 1 |
| Social Questionnaire for Secondary Students (SQSS) | 1 |
| Child- Adolescent Social Support Scale | 1 |
| Peer network section of Substance Abuse and Mental Health Services Administration (SAMHSA) study | 1 |
| Developmental Assets Profile | 1 |
| New 20-item School Belonging Scale | 1 |
| Sense of Belonging to School Scale (SEBES) | 1 |
| Youth Risk Behavior Survey | 1 |
| The Spanish version of the US-based Maryland Safe and Supportive Schools School Climate Survey | 1 |
| The Child Rating Scale (CRS) | 1 |
| Middle Year Development Instrument (MDI) | 1 |
| The Dormitory Belonging Scale | 1 |
| Basic Psychological Needs in Exercise Scale | 1 |
| Sense of Belonging at School Measure | 1 |
| School Bonds Scale | 1 |
| Health Pathway Child Report | 1 |
| Student Teacher Connectedness Measure | 1 |
| Social Capital Questionnaire for Adolescent Students | 1 |
| Community and Youth Collaborative Institute School Experience Scale | 1 |
| Perceived Cohesiveness Scale | 1 |
| Needs Satisfaction in the Workplace Questionnaire | 1 |
| Sense of School Belonging Scale | 1 |
| Loneliness Scale | 1 |
| School Wellbeing Profile | 1 |
| Child and Adolescent Social Support Scale | 1 |
| Connected Classroom Climate Inventory | 1 |
| Tyler’s Work on Institutional Engagement | 1 |
| School Adaptation scale | 1 |
| Attitudes Towards School Survey (ATSS) | 1 |
| Scale of Caring Adult Relationships in School | 1 |
| School Climate Survey | 1 |
| ELS:2002 Base Year Student Questionnaire | 1 |
| Basic Needs Satisfaction in Sport Scale (BNSSS) | 1 |
| Teacher Bonding Scale | 1 |
| Healthy Kids Resilience Measure of School Connectedness | 1 |
| School Climate and Function Scale | 1 |
| School Climate and School Identification Measure-Student (SCAISM-st) | 1 |
| School Climate Scale | 1 |
| Community Scale of the Relational Health Indices for Youth Measure | 1 |
| School Belonging Inventory | 1 |
| Social Questionnaire for Secondary students | 1 |
| School Liking and Avoidance Questionnaire | 1 |
| Survey of Children’s Social Support | 1 |
| Sense of Belonging Instrument | 1 |
| Class Connectedness Scale | 1 |
| New 18-item School Connectedness Scale | 1 |
| New 15-item School Connectedness Scale | 1 |
| Teacher Involvement, Structure, and Autonomy Support Scale | 1 |
| Social Competence Rating Scale for Children (SCRSC) | 1 |
| Learning Climate Questionnaire | 1 |
| School Engagement Subscale of the Drug Free Schools (DFSCA) Outcome Study Questions | 1 |
| Academic Belonging Scale | 1 |
| Classroom Life Measure | 1 |
| Jenkins School Bonding Scale (JSB) | 1 |
| Brief Survey of School Bonding (BSSB) | 1 |
| Adolescent Family and Social Life Questionnaire (AFSLQ) | 1 |
| Teacher as Social Context Questionnaire | 1 |
| Scale developed by Developmental Studies Center | 1 |
| Adolescent Student’s Basic Psychological Needs at School Scale | 1 |
| Relatedness scale | 1 |
| CDC Classroom Climate Scale | 1 |
| Student Engagement Scale | 1 |
| California School Climate and Safety Survey | 1 |
| New 8-item School Connectedness Scale | 1 |
| School Bonding Scale from the National Comorbidity Survey | 1 |
| Interpersonal Behaviors Questionnaire (IBQ) | 1 |
| School Life Characteristic Questionnaire | 1 |

**Supplementary File 4b: Measures of adolescent family connectedness and their frequency of use**

| **Measure** | **Frequency of use** |
| --- | --- |
| Parent-Family Connectedness Scale | 24 |
| Family Adaptation and Cohesion Evaluation Scale (FACES II) | 7 |
| Multigenerational Interconnectedness Scale (MIS) | 6 |
| The Parental Bonding Instrument | 6 |
| Social Connectedness Scale | 6 |
| Basic Psychological Needs Satisfaction Scale (BPNS) | 3 |
| Relationships-With-Mother-Father Questionnaire | 2 |
| Hemingway’s Measure of Adolescent Connectedness (HMAC) | 2 |
| Inventory of Parent and Peer Attachment | 2 |
| Relatedness questionnaire | 2 |
| Child Report of Parental Behavior Inventory | 2 |
| Network of Relationships Inventory (NRI) | 2 |
| Parent-Adolescent Communication Scale (PACS) | 1 |
| Warmth/Support Subscale of the Parenting Styles and Dimensions Questionnaire – Short Version (PSDQ) | 1 |
| Family Environment Scale | 1 |
| Parent-Adolescent Relationship Scale (PARS) | 1 |
| Relatedness Scale of the Rochester Assessment Package for Schools | 1 |
| Family Connectedness Scale | 1 |
| Family Attachment Scale | 1 |
| Multidimensional Scale of Perceived Social Support | 1 |
| The Differentiation in the Family System Scale | 1 |
| Social Bond Measure | 1 |
| Parent Relatedness Scale | 1 |
| Family Affluence Scale | 1 |
| The Mannheim Individuation Questionnaire | 1 |
| Balanced Relatedness Scale | 1 |
| Subjective Family Picture | 1 |
| The Engagement, Perseverance, Optimism, Connectedness, and Happiness (EPOCH) measure of Adolescents Wellbeing | 1 |
| Family Implicit Rules Profile (FIRP) | 1 |
| Scale from the Minnesota Adolescent Health Survey and Youth Risk Behavior Survey | 1 |
| Family Assessment Measure–3rd edition (FAM-III) | 1 |
| National Survey of Child Health | 1 |
| Family Relatedness Scale | 1 |
| Positive Youth Development Measure | 1 |
| Family Assessment Clinician Interview (FACI) | 1 |
| Parenting Style Index | 1 |
| 36 items of the Egna Minnen Betraffande Uppfostran for Adolescents (EMBU- A) | 1 |
| Family Sense of Belonging Scale | 1 |
| Teen Supplemental Survey-Connectedness with Caregivers Subsection | 1 |
| The Modiﬁed Friendship Scale (MFQS) | 1 |
| The Family Belonging Scale-Revised (FBS-R) | 1 |
| Youth Risk Behavior Survey | 1 |
| Youth Relationship with Parent Index | 1 |
| Father-Son Closeness and Connectedness Scale | 1 |
| Family Climate Inventory | 1 |
| Attitudes toward Accompanied Driving Scale (ATADS) | 1 |
| Health Behavior in School Children’s (HBSC) Survey Questionnaire | 1 |
| Youth Asset Survey | 1 |
| Family Resilience Assessment Scale | 1 |
| Adolescent Promoting and Relatedness Scale | 1 |
| Parental Behavior Measure | 1 |
| Child Intrinsic Needs Satisfaction Scale | 1 |
| Parent-Child Relationship Quality Scale | 1 |
| Subjective Family Image Test | 1 |
| Voice of Connecticut Youth Survey | 1 |
| Multitrait-Multimethod Model | 1 |
| Hofer Connectedness Scale | 1 |
| Relationship Problem Inventory | 1 |
| Parent-Child Relationship Scale | 1 |
| Inclusion of Others in Self | 1 |
| Attitudinal Familism Scale | 1 |
| UCLA Loneliness Scale | 1 |
| Autonomy and Relatedness Coding System | 1 |
| Relatedness Scale | 1 |
| Eco-Cultural Family Interview | 1 |
| Basic Psychological Needs in Exercise Scale | 1 |
| Interpersonal Needs Questionnaire (INQ) | 1 |
| New 4-item Family Connectedness Scale | 1 |
| New 10-item Family Connectedness Scale | 1 |
| New 11-item Family Connectedness Scale | 1 |

**Supplementary File 4c: Measures of adolescent community connectedness and their frequency of use**

| **Measure** | **Frequency of use** |
| --- | --- |
| Sense of Belonging Instrument – Psychological Subscale (SOBI-P) | 5 |
| Community Connectedness Scale | 3 |
| Neighborhood Youth Inventory (NYI) | 3 |
| Sense of Community Index | 2 |
| Sense of Community Scale for Adolescents (SOC-A) | 2 |
| Neighborhood Intergenerational Closure scale | 2 |
| Collective Efficacy Scale (CES) | 2 |
| Hemingway’s Measure of Adolescent Connectedness (HMAC) | 1 |
| Scale measuring Sense of Belonging to the European Union | 1 |
| Sense of Community Index-Primary (SCI-P) | 1 |
| Belonging Scale | 1 |
| Neighborhood Cohesion Instrument | 1 |
| Psychological Sense of Community | 1 |
| Sense of Community Membership | 1 |
| Social Context Virtual Subscale (SSC-VC) | 1 |
| Middle Years Development Instrument (MDI) | 1 |
| Sense of Belonging Questionnaire | 1 |
| The Brief Implicit Association Test | 1 |
| Inclusion of Community in the Self Scale | 1 |
| Social Support Rate Scale | 1 |
| Personal Wellbeing Index | 1 |
| Inclusion of Others in Self | 1 |
| Community Connectedness Measure from the Add Health Study | 1 |
| Basic Need Satisfaction at Work Scale | 1 |
| Social Disconnection Scale | 1 |
| Neighbors and Sense of Community Subscales | 1 |
| Brief Scale of Sense of Community in Adolescents | 1 |
| Need for Relatedness Scale | 1 |
| Psychological Acculturation Scale | 1 |
| Neighborhood Environment Scale | 1 |
| School Belonging Measure | 1 |
| Active and Engaged Citizenship Scale | 1 |
| School Connectedness Scale | 1 |
| No Mobile Phone Questionnaire (NMP-Q) | 1 |
| Neighborhood Connection Scale | 1 |
| Me and My Neighborhood Questionnaire | 1 |
| Neighborhood Cohesion Scale | 1 |
| Need to Belong Scale | 1 |
| British Columbia Adolescent Health Survey (BCAHS) | 1 |
| Gender Minority Stress and Resilience Measure | 1 |

**Supplementary File 4d: Measures of adolescent peer connectedness and their frequency of use**

| **Measure** | **Frequency of use** |
| --- | --- |
| Hemingway’s Measure of Adolescent Connectedness (HMAC) | 6 |
| Need for Relatedness Scale | 6 |
| Basic Psychological Needs Satisfaction Scale (BPNS) | 5 |
| Basic Psychological Needs in Exercise Scale | 3 |
| Peer Connectedness Measure from the Add Health Study | 2 |
| Peer Motivational Climate in Youth Sport Questionnaire (PeerMCYSQ) | 2 |
| Relational Provision Loneliness Questionnaire (RPLQ) | 2 |
| Social Identity Scale | 1 |
| Chestnut Lodge Adolescent Interaction Scale | 1 |
| The Child Loneliness Scale | 1 |
| Social Isolation Questionnaire for Adolescents (CSIQ-A) | 1 |
| Sense of Community Scale for Adolescents (SOC-A) | 1 |
| The Engagement, Perseverance, Optimism, Connectedness, and Happiness (EPOCH) Measure of Adolescents Wellbeing | 1 |
| Health Behavior in School Children’s (HBSC) Survey Questionnaire | 1 |
| Interpersonal Support Evaluation List, College Version (ISEL2 ) | 1 |
| Neediness and Relatedness subscales of the Depressive Experiences Questionnaire for Adolescents (DEQ–A) Interpersonal Concerns factor | 1 |
| Classmate Social isolation Questionnaire CISQ-A | 1 |
| The Loneliness and Social Dissatisfaction Questionnaire | 1 |
| New 7-item Peer Connectedness Scale | 1 |
| Youth Connections Scale | 1 |
| Psychological Needs Satisfaction in Education Scale | 1 |
| Sense of Community Scale | 1 |
| School Adaptation Scale | 1 |
| Autonomy–Connectedness Scale | 1 |
| The Peer Involvement in Delinquent Acts Scale (PIDAS) | 1 |
| Youth Risk Behavior Survey | 1 |
| Group Belonging Scale | 1 |
| Neighborhood Youth Inventory (NYI) | 1 |
| Peer Scale of the Self-Description Questionnaire | 1 |
| The High School Questionnaire (HSQ) | 1 |
| Measure of Peer Affiliation | 1 |
| Quality of Student Life Questionnaire | 1 |
| Motivational Climate in Physical Education Scale | 1 |
| Balanced Relatedness scale | 1 |
| Inclusion of Others in Self | 1 |
| Peer Support Scale | 1 |
| Relatedness Scale | 1 |
| Child and Adolescent Social Support Scale | 1 |
| UCLA Loneliness Scale-Revised | 1 |
| Interpersonal Needs Questionnaire (INQ) | 1 |
| Loneliness and Social Dissatisfaction Questionnaire for Young Children | 1 |
| Social Questionnaire for Secondary students | 1 |
| The Peer Bond Scale | 1 |
| Classroom Life Measure | 1 |
| Youth Connection Scale – Child (YCS-C) | 1 |
| New 3-item Peer Relationships Scale | 1 |
| New 5-item Peer Relatedness Scale | 1 |
| Basic Psychological Needs in Physical Education Scale | 1 |
| Basic Needs Satisfaction in Sport Scale (BNSSS) | 1 |
| Self-Description Questionnaire (SDQ-II) | 1 |
| Peer subscale of the Strengths and Difficulties Questionnaire (SDQ) | 1 |
| New 10-item Belonging Measure | 1 |
| Harter’s Perceived Competence Scale for Children | 1 |
| Basic Needs Questionnaire for Children (BNQ-C) – Relatedness Subscale | 1 |

**Supplementary File 4e: General measures of adolescent connectedness and their frequency of use**

| **Measure** | **Frequency of use** |
| --- | --- |
| Social Connectedness Scale | 37 |
| Hemmingway’s Measure of Adolescent Connectedness (HMAC) | 16 |
| Basic Psychological Needs Satisfaction Scale (BPNS) | 15 |
| Interpersonal Needs Questionnaire (INQ) | 7 |
| The Engagement, Perseverance, Optimism, Connectedness, and Happiness (EPOCH) Measure of Adolescent Wellbeing | 6 |
| Resiliency Scales for Children and Adolescents | 3 |
| Health Behavior in School Children’s (HBSC) Survey Questionnaire | 3 |
| General Belongingness Scale | 3 |
| Children’s Intrinsic Needs Satisfaction Scale | 2 |
| Multigroup Ethnic Identity Measure (MEIM) | 2 |
| Quality of Students Life Questionnaire (QSLQ) | 2 |
| Relatedness Scale | 2 |
| Middle Years Development Instrument (MDI) | 2 |
| UCLA Loneliness Scale | 2 |
| Self in a Social Context – Social Connectedness Scale | 2 |
| Awareness of Connectedness Scale (ACS) | 2 |
| Milwaukee Youth Belongingness Scale (MYBS) | 2 |
| Motivational Climate in Physical Education Scale (MCPES) | 1 |
| Exeter Identity Transition Scale (EXITS) | 1 |
| Ethnic Identity Scale | 1 |
| State Social Disconnection Scale | 1 |
| The Adolescent Resilience Questionnaire | 1 |
| Interview Schedule for Social Interaction | 1 |
| Need to Belong Scale | 1 |
| Scale of Satisfaction with Life Support (SSSS) – Satisfaction with Social Support (SSS) and Need for Activities Connected to Social Support (NASS) Dimensions | 1 |
| Millon Adolescent Clinical Inventory | 1 |
| Fisher’s Spiritual Wellbeing Scale | 1 |
| Interpersonal Relationship Quality Scale | 1 |
| Motivation to Remain Friends Questionnaire | 1 |
| Resilience Scale | 1 |
| Vaux Social Support Record | 1 |
| Australian Community Participation Questionnaire | 1 |
| Interpersonal Support Evaluation List (ISEL) | 1 |
| Social Bonding Scale | 1 |
| Social Comparison Scale | 1 |
| Measure of Attitudes Toward Social Networking Sites (MATS) | 1 |
| Social Relatedness Scale | 1 |
| Depressive Experiences Questionnaire for Adolescents (DEQ-A) | 1 |
| Classroom Sense of Community Scale (SoC-C) | 1 |
| Social Safeness and Pleasure Scale | 1 |
| Existence, Relatedness and Growth Scale | 1 |
| Perceived Emotional / Personal Support Scale | 1 |
| The Quality of Life Profile – Adolescent Version | 1 |
| What is Happening in This School (WHITS) Questionnaire | 1 |
| Sense of Belonging to Group Checklist | 1 |
| State of Victoria’s (Australia) Department of Education, Employment and Training Secondary School Questionnaire | 1 |
| General Connectedness Scale for Children | 1 |
| Contextualized Assessment Tool for Risk and Protection Management (CATPRM) | 1 |
| SAMHSA Government Performance and Results Act (GPRA) Participant Outcome Measures for Discretionary Programs | 1 |
| School, Peer, Family, and Community Subscales from the Indian Urban Youth Survey | 1 |
| New 8-item Social Connectedness Scale | 1 |
| Multidimensional Scale of Perceived Social Support | 1 |
| Sociotropy-Achievement Scale for Children (SASC) – Connectedness Subscale | 1 |
| Needs Satisfaction Scale | 1 |
| School Connectedness Scale from the Minnesota Adolescent Health Survey | 1 |
| Sense of Community in Sports Scale | 1 |
| Sense of Belonging Instrument – Psychological Subscale (SOBI-P) | 1 |
| Child and Youth Resilience Measure (CYRM) | 1 |
| Intrinsic Need Satisfaction Scale in Mobile Communication – Relatedness Subscale | 1 |
| Depressive Experiences Questionnaire (DEQ) | 1 |
| Integration Feeling Questionnaire | 1 |
| 4-item Ethnic Identity-Oyserman | 1 |
| Sense of Belonging Scale for Adolescents (SOBS) | 1 |
